# Mannich-type modifications of (−)-cannabidiol and (−)-cannabigerol leading to new, bioactive derivatives

**DOI:** 10.1038/s41598-023-45565-7

**Published:** 2023-11-10

**Authors:** Eszter Boglárka Lőrincz, Gergely Tóth, Júlia Spolárics, Mihály Herczeg, Jan Hodek, István Zupkó, Renáta Minorics, Dorottya Ádám, Attila Oláh, Christos C. Zouboulis, Jan Weber, Lajos Nagy, Eszter Ostorházi, Ildikó Bácskay, Anikó Borbás, Pál Herczegh, Ilona Bereczki

**Affiliations:** 1 Department of Pharmaceutical Chemistry, University of Debrecen, Debrecen, 4032 Hungary; 2 Doctoral School of Pharmaceutical Sciences, University of Debrecen, Debrecen, 4032 Hungary; 3 Institute of Organic Chemistry and Biochemistry of the Czech Academy of Science, Prague, 166 10 Czech Republic; 4 Institute of Pharmacodynamics and Biopharmacy, University of Szeged, Szeged, 6720 Hungary; 5 Department of Physiology, Faculty of Medicine, University of Debrecen, Debrecen, 4032 Hungary; 6 Doctoral School of Molecular Medicine, University of Debrecen, Debrecen, 4032 Hungary; 7 Departments of Dermatology, Venereology, Allergology and Immunology, Staedtisches Klinikum Dessau, Brandenburg Medical School Theodor Fontane and Faculty of Health Sciences Brandenburg, 06847 Dessau, Germany; 8 Department of Applied Chemistry, University of Debrecen, Debrecen, 4032 Hungary; 9 Department of Medical Microbiology, Semmelweis University, Budapest, 1089 Hungary; 10 Department of Pharmaceutical Technology, University of Debrecen, Debrecen, 4032 Hungary; 11 National Laboratory of Virology, Szentágothai Research Centre, Pécs, 7624 Hungary; 12 HUN-REN–UD Molecular Recognition and Interaction Research Group, University of Debrecen, Debrecen, 4032 Hungary; 13 HUN-REN–UD Pharmamodul Research Group, University of Debrecen, Debrecen, 4032 Hungary

**Keywords:** Chemical biology, Drug discovery, Chemistry

## Abstract

(−)-Cannabidiol (CBD) and (−)-cannabigerol (CBG) are two major non-psychotropic phytocannabinoids that have many beneficial biological properties. However, due to their low water solubility and prominent first-pass metabolism, their oral bioavailability is moderate, which is unfavorable for medicinal use. Therefore, there is a great need for appropriate chemical modifications to improve their physicochemical and biological properties. In this study, Mannich-type reaction was used for the synthetic modification of CBD and CBG for the first time, and thus fifteen new cannabinoid derivatives containing one or two tertiary amino groups were prepared. Thereafter the antiviral, antiproliferative and antibacterial properties of the derivatives and their effects on certain skin cells were investigated. Some modified CBD derivatives showed remarkable antiviral activity against SARS-CoV-2 without cytotoxic effect, while synthetic modifications on CBG resulted in a significant increase in antiproliferative activity in some cases compared to the parent compound.

## Introduction

(−)-Cannabidiol (CBD) (**1**) and (−)-cannabigerol (CBG) (**2**) are among the most studied non-psychotropic phytocannabinoids present in *Cannabis sativa* L^1,2^. CBD is the second main active component of the plant after (−)-*trans*-Δ^9^-tetrahydrocannabinol (THC). It is approved as a drug (Epidiolex^®^)^3^ in the US as well as in certain European countries for the treatment of two rare types of epilepsy, Dravet syndrome (DS) and Lennox–Gastaut syndrome and for the treatment of tuberous sclerosis complex (TSC). Sativex^®^^4^, containing CBD and THC in 1:1 ratio, is also approved in several countries against multiple sclerosis-associated spasticity. Furthermore, several other beneficial effects are attributed to CBD. CBG is often referred to as “the mother” of all phytocannabinoids, because other phytocannabinoids are derived from cannabigerolic acid, a carboxylated form of CBG. CBG has no medical application yet, but it is used as supplement and its pharmacological effects are studied intensively^5^.

CBD and CBG are known to concentration-dependently affect several receptors in the human body, among others cannabinoid receptors CB_1_ and CB_2_, *α-*2 adrenoceptor, certain serotonin (5-HT) receptors, peroxisome proliferator-activated receptors (PPARs) and multiple ligand-gated ion channels^5,6^. CBD and CBG are proven to have analgesic, neuroprotective, anti‐inflammatory, metabolic, anti‐tumor, gastrointestinal and cardiovascular effects by the activation of PPARs^7^ that are nuclear hormone receptors regulating energy homeostasis, lipid and carbohydrate metabolism, as well as inflammation. These receptors have important roles in diabetes, adipocyte differentiation, cancer, lung diseases, neurodegenerative disorders, fertility or reproduction, pain, and obesity^8^.

Phytocannabinoids are widely used for palliative treatment of cancer patients to relieve pain and nausea and to stimulate appetite. Moreover, CBD and CBG were shown to exert promising antitumor effects in several model systems, among others, in the case of brain tumors^9,10^ and human breast cancer types^11^, but unfortunately protumorigenic activity of natural cannabinoids has also been reported^12,13^, and their (most likely detrimental) effect on the antitumor immune response must also be investigated^14^.

Regulation of endocannabinoid system in the skin has an important role in the maintenance of cutaneous homeostasis, barrier formation and regeneration. Several lines of evidence argue that CBD, CBG, as well as other cannabinoids may have the potential to treat various skin diseases^15–17^. Indeed, without being exhaustive, cannabinoids have been shown to exert promising effects against acne vulgaris, allergic contact dermatitis, asteatotic dermatitis, atopic dermatitis, hidradenitis suppurativa, Kaposi sarcoma, pruritus, psoriasis, certain skin cancers, and the cutaneous manifestations of systemic sclerosis^15,16,18^. Moreover, topically applied synthetic CBD exerted clinically relevant anti-acne effects in a placebo-controlled phase 2 clinical trial (ClinicalTrials.gov ID: NCT03573518), and positive effects were reported in rosacea (phase 1b/2)^19^; whereas it was found not to be superior as compared to placebo in atopic dermatitis in a phase 2 study (ClinicalTrials.gov ID: NCT03824405).

Furthermore, recently anti-SARS-CoV-2^20^ and antibacterial properties of CBD^21^ have also been published, and CBD was reported to be efficient in achieving nasal decolonization of *Staphylococcus aureu*s in a small phase 2a study (ClinicalTrials.gov ID: NCT03824405).

**Figure 1:**
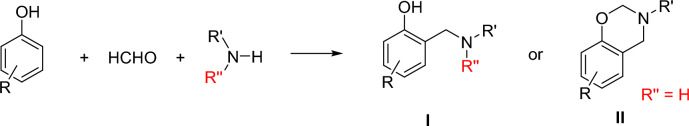
Mannich-type reaction of phenols.

In a chemical approach, synthetic modifications of CBD and CBG have not been throroughly investigated. Only a few types of systematic synthetic modifications of CBD and CBG can be found in the scientific literature, such as oxidative modification of the aromatic ring^22,23^, ether formation on the phenolic OH by Mitsunobu reaction^24^ and the synthesis of carbamate derivatives^25^. We therefore set out to chemically modify CBD and CBG to alter and (hopefully) improve their selected biological activities (e.g., effects on skin cells, anticancer, antiviral and antibacterial properties) and possibly their water solubility. The resorcinol structure of CBD and CBG gives opportunities for different chemical modifications, including Mannich-type reactions, by which an aminomethyl side chain can be introduced into the ortho-position of the phenolic moiety (Fig. 1). Introducing an N-containing functionality into cannabinoid backbones can be beneficial as nitrogen-containing compounds have long been a valuable source of therapeutic agents in medicinal chemistry^26,27^. The nitrogen atom is capable of forming Coulomb interactions, hydrogen bonds and weak interactions including van der Waals forces and dipole–dipole interactions, which enable amino-containing compounds to bind with high affinity to various enzymes and receptors as biological targets. Moreover, the amino group introduced into the molecule may increase the water solubility of the derivatives by salt formation, which can result in better bioavailability compared to the parent compounds. It is important to note that CBD and CBG are sensitive to oxidation^28,29^ for which the resorcinol part is mainly responsible; we assumed that the planned modification of this part can help to increase the stability of the compounds against oxidation.

The classical Mannich reaction is a three-component amino alkylation reaction with a formaldehyde and a primary or secondary amine or ammonia on a carbon atom containing acidic proton next to a carbonyl functional group^30^. Mannich-type reaction of phenols is less known. Aminomethylation of the carbon atom next to the phenolic OH group takes place with secondary or primary amines and formaldehyde (I, Fig. 1). However, when primary amines are used, ring closure with another formaldehyde molecule and the phenolic OH resulting in an oxazine-ring product was observed (II, Fig. 1)^31–34^. This kind of Mannich reaction with β-naphtols is also known as Betti reaction^35,36^.

Considering the above, and the fact that no Mannich-type reactions have been performed on cannabinoids, we intended to study the Mannich-type reactions of CBD and CBG introducing amino side chains next to the phenolic OH groups, and we planned to investigate the biological effects of the produced derivatives. Different, commercially available, simple primary and secondary amines were used to modify CBD and CBG and we found that reactions with primary amines produced exclusively oxazine derivatives (type II, Fig. 1), whereas open-chain products (type I, Fig. 1) were formed when secondary amines were used.

The new semi-synthetic CBD and CBG derivatives obtained were tested for anti-cancer, antibacterial and antiviral activity and we also studied their effects on certain skin cells.

## Results

### Synthesis

**Figure 2:**
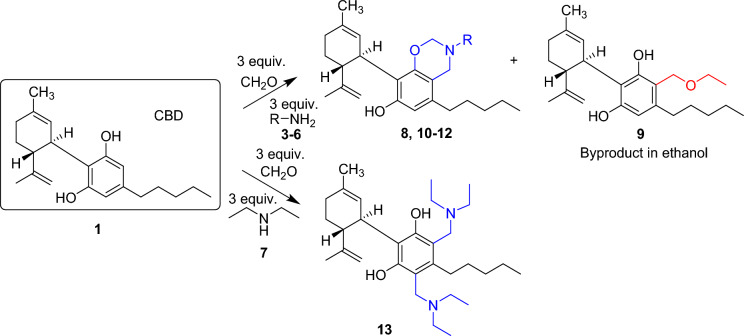
Reaction of cannabidiol (CBD) with amines and formaldehyde in Mannich-type reaction (reaction conditions and yields are shown in Table 1).

First, the Mannich-type reaction of CBD (Fig. 2) was studied using different amines. Since this type of cannabinoid derivatives had not yet been prepared, we selected a wide variety of simple amines, such as primary and secondary, aliphatic and aromatic, saturated and unsaturated amines, as well as amines containing other functional groups, and used them in Mannich-type reactions of cannabinoids. We used *n*-butylamine **3** and benzylamine **4** as normal and aryl-containing alkylamines with similar lipophilicity, and heterobifunctional alkylamines such as propargylamine **5** and γ-aminobutiric acid (GABA) **6.** The carbon–carbon triple bond of **5** enables further derivatization by alkyne-azide click reaction, while the carboxyl group of GABA provides the possibility for various transformations of the Mannich-type product by ester, amide and salt formation. Next, diethylamine **7** was chosen as the secondary amine reagent because its lipophilicity is similar to that of primary amine **3**.

**Table 1:** Reaction conditions and yields of Mannich-type reactions of CBD with amines and formaldehyde.

Reaction	Amine	Solvent	Reaction time	Temp	Product	Yield
*A1*		MeOH	7 days	rt		89%
*A2*		EtOH	20 h	Reflux		**8**: 64% **9**: 21%
*A3*		EtOH	6 h 18 h	Reflux 45 °C		100%
*A4*		Dioxane	6 h 18 h	Reflux 50 °C		60%
*B1*		Dioxane	6 h 18 h	Reflux 70 °C		48%
*B2*		Dioxane	9 days	50 °C		94%
*C1*	5 equiv. CH_2_O 5 equiv. amine	Dioxane	2 days 3 days	Reflux 80 °C		46%
*C2*		Dioxane	9 days	50 °C		94%
*D*		Dioxane	4 days	Reflux at day and 70 °C at night		32%
*E*		Dioxane	6 h 18 h	Reflux 70 °C		67%

Treatment of CBD with 3 equivalents of formaldehyde and 3 equivalents of *n*-butylamine at room temperature provided exclusively the oxazine-containing product **8** with high yield. However, the reaction was very sluggish: its completion required 7 days^37^. To accelerate the reaction, the solvent was changed to ethanol and the reaction was heated overnight at reflux temperature. In this way, the reaction went to completion in 20 h, but a significant amount of side product (**9**)^37^ was observed in the reaction of CBD with ethanol and formaldehyde. To avoid the side reaction, dioxane was chosen as solvent in the next reaction, which was carried out under reflux for 6 h and 18-h heating at 50 °C, but the yield was not as good as in the first reaction in methanol. Since alcohols seemed to be better solvents, the reaction was repeated in ethanol under reflux for 6 h followed by heating at 45 °C for 18 h, and in this case, the yield reached ~ 100%.

The reaction of CBD with benzylamine (**4**) or propargylamine (**5**) was carried out in dioxane (to avoid the side reaction with alcohol) by heating at different temperatures (50 °C to reflux); the lower temperature with a longer reaction time (9 days) again proved to be better resulting in higher yields.

The reaction of CBD with γ-aminobutyric acid (**6**) was less effective, the reaction mixture was heated and monitored by TLC for 4–5 days, but unfortunately the reaction was very slow and the yield was moderate.

In the case of the secondary diethylamine (**7**), the previously optimized reaction conditions were used. In dioxane, after 6 h under reflux and 18 h of heating at 70 °C a disubstituted product (**13**)^37^ was formed surprisingly, which was not observed in the reactions with primary amines.

**Figure 3:**
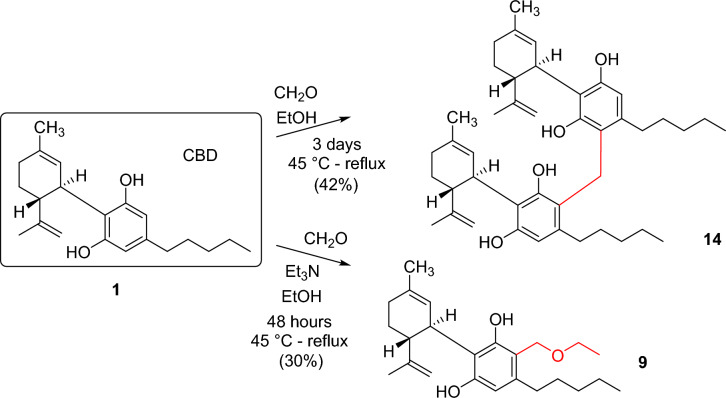
Investigation of the production of the byproduct **9** bearing an ethoxymethyl side chain.

In order to investigate the side reaction of CBD mentioned earlier, the reaction in ethanol was repeated without butylamine in the presence of formaldehyde (Fig. 3). The reaction mixture was heated for 3 days and continuously monitored by TLC. Surprisingly, the expected compound **9** was not formed in the reaction. The isolated product was identified by mass and NMR spectroscopic analysis as a dimeric compound (**14**) formed from two CBDs and one formaldehyde molecule. This reaction is similar to the formation of calixarenes^38^. Calixarenes are cyclic oligomers composed of methylene linked phenols generally produced from phenols and formaldehyde under alkaline conditions in a condensation reaction. However, we used neutral conditions under which, the formation of the methylene-bridged compound **14** is surprising and worthy of further investigation. On the other hand, the experiment performed under neutral conditions proved that the formation of the ethoxymethyl-substituted derivative **9** requires basic conditions. To ensure this we used triethylamine, which, being a tertiary amine, cannot participate in Mannich-type reaction. In this way, although full conversion could not be achieved, the desired CBD derivative **9** containing an ethoxymethyl side chain was isolated with 30% yield. This type of reaction, when resorcinol-type compounds react with alcohols and aldehydes, especially formaldehyde, are also known from the literature^39–41^, typically using iminodiacetic acid as the amine reagent. Triethylamine was also used by Hidalgo et al.^42^, but they observed very low yields (3–14%).

**Figure 4:**
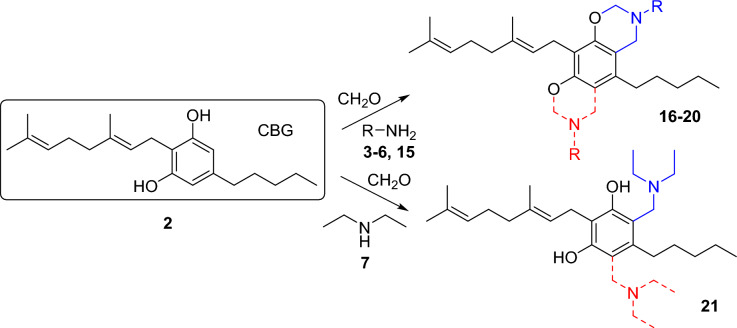
Reaction of CBG with amines and formaldehyde in Mannich-type reaction. (Reaction conditions and yields are shown in Table 2).

CBG was also reacted with the previously used different types of primary and secondary amines including *n*-butylamine **3**, benzylamine **4**, propargylamine **5**, γ-aminobutyric acid **6** and diethyalamine **7** in Mannich-type reactions (Fig. 4. Table 2). Moreover, as the reaction with propargylamine proceeded with low efficiency (vide infra), an additional heterobifunctional amine, allylamine **15** was used that also gives the opportunity to further derivatisations of the Mannich-type product by the thiol-ene click reaction^43^.

**Table 2:** Reaction conditions and yields of Mannich-type reactions of CBG with amines and formaldehyde.

Reaction	R-NH_2_	Solvent	Reaction time	Temp.	Product	Yield
*F1*	20 equiv. (CH_2_O)_n_ 6 equiv. amine	EtOH	3 h	Reflux		10%
*F2*	5 equiv. CH_2_O 5 equiv. amine	EtOH	20 h	Reflux		67% impure
*F3*		Dioxane	48 h	Reflux at day and 70 °C at night		52%
*G*	5 equiv. benzylamine 5 equiv. CH_2_O	Dioxane	8 h 18 h	Reflux 70 °C		64%
						35%
*H*	2 equiv. propargylamine 2 equiv. CH_2_O	Dioxane	48 h 48 h	rt 70 °C		19%
*I*	2 equiv. allyl amine 2 equiv. CH_2_O	Dioxane	6 days	45 °C		66%
						27%
*J*	2 equiv. GABA 2 equiv. CH_2_O	Dioxane/water	7 days	rt		71%
*K1*	3 equiv. amine 3 equiv. CH_2_O	EtOH	6 h 18 h	Reflux 70 °C		67%
*K2*		Dioxane	48 h	Reflux at day and 70 °C at night		61%

When ethanol was used as solvent in the reaction with *n*-butylamine, after heating at reflux temperature for 3 h, only the monosubstituted derivative **16a** was detected in a poor yield, while after boiling for 20 h, mainly the disubstituted derivative **16b** was yielded. Unfortunately, the product was not pure after column chromatography, in the NMR spectra a by-product containing ethoxymethyl side chain was also detected. Changing the solvent to dioxane resulted in a lower yield and a longer reaction time; nevertheless, the further reactions were performed in dioxane to avoid possible side reactions with the solvent ethanol. In the case of benzylamine (**4**) and allylamine (**15**), monosubstituted derivatives (**17a** and **19a**) as well as disubstituted products (**17b** and **19b**) were also detected, while in the reaction with propargylamine (**5**) and GABA (**6**) only monosubstituted derivatives **18** and **20** were obtained. The reaction with diethylamine (**7**) resulted in monosubstituted **21a** derivative when the reaction was heated at reflux temperature for 6 h and at 70 °C for 18 h and disubstituted **21b** derivative was produced with longer reaction time.

In order to model the salt preparation from the synthesized derivatives, compound **8** was transformed into a HCl salt in anhydrous diethyl ether with HCl gas derived from the reaction of NaCl and sulfuric acid to yield the HCl salt **8s**.

### Antiproliferative actions

**Figure 5:**
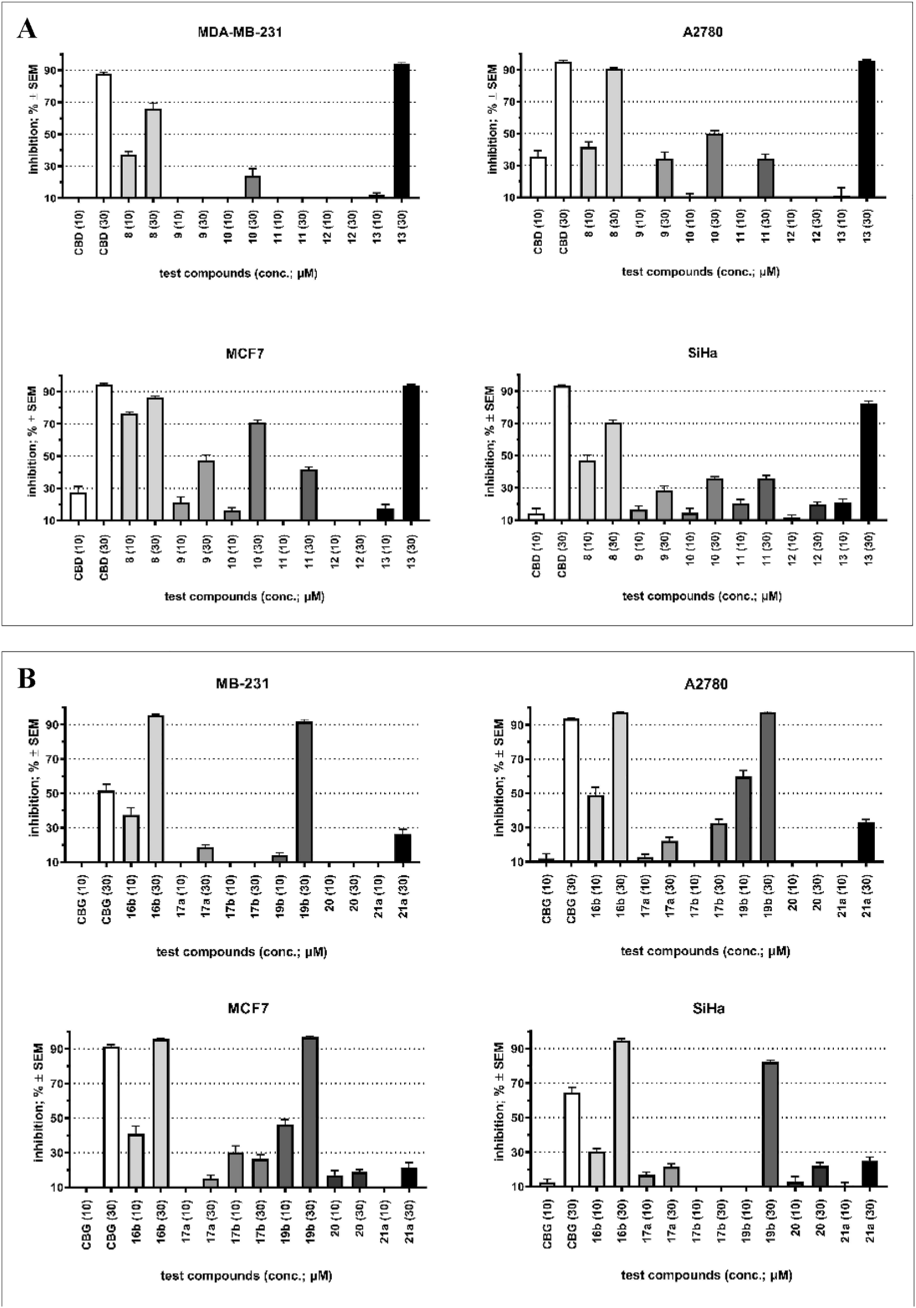
(**A**) Antiproliferative properties of CBD and its tested analogs (10 and 30 μM). (**B**) Antiproliferative properties of CBG and its tested analogs (10 and 30 μM). Inhibition values less than 10% are considered negligible and not presented for clarity.

The antiproliferative properties of natural cannabinoids and their derivatives are thoroughly investigated, therefore we examined CBD, CBG and their derivatives (CBD derivatives: **8**, **9**, **10**, **11**, **12**, **13**; CBG derivatives: **16b**, **17a**, **17b**, **19b**, **20**, **21a**) against four different cancer cell lines (MDA-MB-231: a highly aggressive breast cancer cell line; A2780: epithelial ovarian cancer cell line; MCF7: a slow-growing breast cancer cell line; SiHa: derived from squamous cervix carcinoma) in two concentrations (10 and 30 μM). CBD exerted substantial (i.e., ~ 90%) inhibition of the proliferation of the used cancer cell lines at 30 μM. Still, its effect at 10 μM was modest (on A2780 and MCF7 cells) or negligible (on MDA-MB-231 and SiHa cells, Fig. 5A). Compound **8** exerted an effect similar to CBD’s; moreover, MCF7 cells proved to be more sensitive to the modified derivative **8**. Compound **10** elicited a considerable growth inhibition of MCF7 cells at a higher concentration. Analog **13** was similarly effective than CBD at 30 μM, but no relevant action was obtained at 10 μM. None of the other tested CBD analogs (**9**, **11**, and **12**) exhibited substantial antiproliferative activity in the used concentrations. On the other hand, CBG exerted less pronounced action than CBD; higher than 90% growth inhibition was observed against A2780 and MCF7 cells (Fig. 5B). Remarkably, two tricyclic analogs (**16b** and **19b**) proved to be more active than CBG against all four cancer cell lines. All other presented compounds exerted negligible antiproliferative properties.

The antiproliferative effect of salt derivative **8s** was also investigated compared to the free base derivative **8**, but no significant difference was detected (Supplementary Information Fig. S1).

### Investigations on skin cells

As mentioned above, phytocannabinoids were shown to greatly influence biological activity of various skin cells, including epidermal keratinocytes and sebocytes^16,44–47^. Thus, we decided to investigate two lead compounds in each group (CBD derivatives: **8** and **9**; CBG derivatives: **16a** and **21b**) along with the respective mother molecules **1** and **2** by using HaCaT keratinocytes as well as SZ95 sebocytes (widely used human, immortalized keratinocyte and sebocyte model systems, respectively)^48–50^.

**Figure 6:**
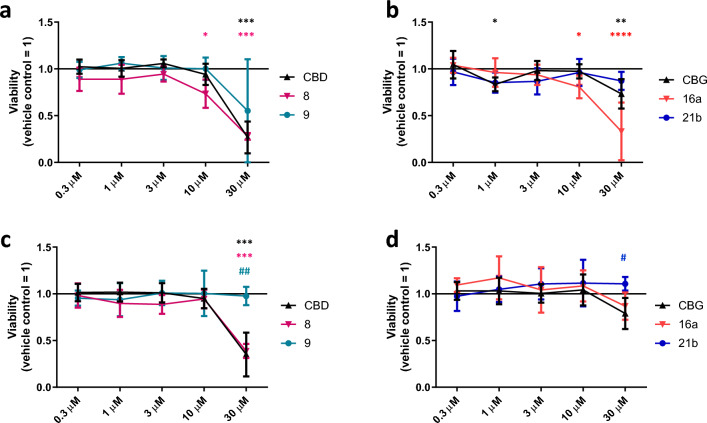
Effects of CBD, CBG, as well as of their selected derivatives on human epidermal HaCaT keratinocytes (**a**,**b**) and human SZ95 sebocytes (**c**,**d**). MTT-assay. Cells were treated as indicated for 48 h. Data are presented following their normalization to the mean of the vehicle-treated control group (regarded as 1) as mean ± SD of N = 8 biological replicates. *, **, ***, and ****P < 0.05, 0.01, 0.001, and 0.0001 compared to the vehicle-treated control. ^#^ and ^##^*P* < 0.05 and 0.01 compared to the group treated with identical CBD or CBG concentration. Color of the symbols indicates the compound they correspond to. CBD: (−)-cannabidiol; CBG: (−)-cannabigerol.

First, we compared the effects of the said compounds on the viability of both cell types. As shown on Fig. 6, despite of some subtle alterations, no significant differences were observed between the mother compounds and the respective derivatives in case of HaCaT keratinocytes (48-h treatments; Fig. 6a,b). Intriguingly, however, in case of SZ95 sebocytes, both the CBD derivative **9** and the CBG derived **21b** were found to behave significantly differently as compared to the respective mother compounds (Fig. 6c,d). Indeed, when applied at 30 μM, both CBD and **8** were found to exert cytotoxic effects (marked decrease in MTT signal intensity), whereas 30 μM of **9** had no such effect on the viability (Fig. 6c). Likewise, when applied at 30 μM, CBG and compound **16a** tended to suppress viability of SZ95 sebocytes, whereas **21b** rather increased MTT signal intensity (Fig. 6d).

**Figure 7:**
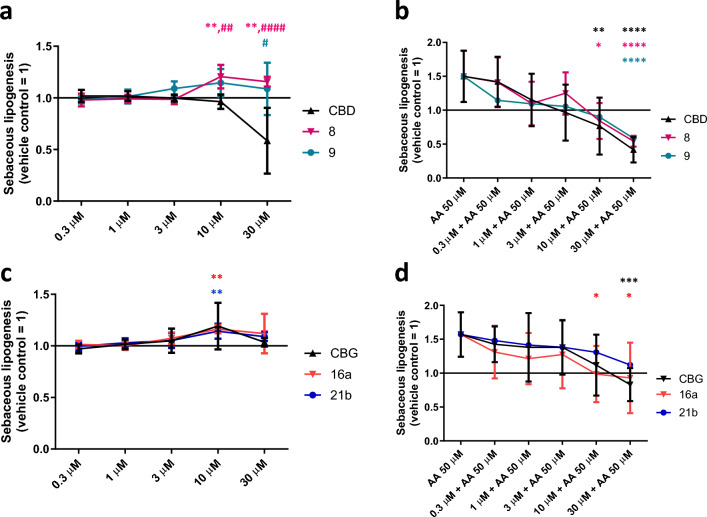
Effects of CBD, CBG, as well as of their selected derivatives on human SZ95 sebocytes alone (**a**,**c**) or in the presence of 50 μM arachidonic acid (AA) (**b**,**d**). Nile Red-assay. Cells were treated as indicated for 48 h. Data are presented following their normalization to the mean of the vehicle-treated control group (regarded as 1) as mean ± SD of N = 8 biological replicates. *, **, and ****P < 0.05, 0.01, and 0.0001 compared to the AA-treated control (**a**,**c**) or to the AA-treated group (**b**,**d**). ^#^, ^##^, and ^####^*P* < 0.05, 0.01, and 0.0001 compared to the group treated with identical CBD or CBG concentration. Color of the symbols indicates the compound they correspond to. AA: arachidonic acid; CBD: (−)-cannabidiol; CBG: (−)-cannabigerol.

These data indicated that, albeit being similar in structure, the new compounds may have different cellular targets in human skin-derived cells. Thus, to further dissect these putative functional differences between them, we also assessed their effects on sebaceous lipogenesis (Fig. 7). In line with our previous findings^44,45^ CBD (≤ 10 μM) had no effect on the spontaneous, homeostatic sebaceous lipogenesis (Fig. 7a), while at the highest test concentration (30 μM), we observed a significant decrease in the lipid synthesis (Fig. 7a). This was most likely due to the aforementioned cytotoxic effect (Fig. 6c). Notably, in a perfect agreement with our previously reported data^44^, CBD could completely prevent the lipogenic effect of the “acne-mimicking” pro-inflammatory lipid mediator arachidonic acid (AA)^48,51^ (Fig. 7b). Importantly, both compounds **8** and **9** could mimic this lipostatic effect (Fig. 7b). However, when applied alone, both CBD derivatives were found to rather promote spontaneous, homeostatic sebaceous lipogenesis, and this effect was statistically significant in case of compound **8** (10 and 30 μM) (Fig. 7a).

On the other hand, when applied alone, compounds **16a** and **21b** mimicked the previously reported weak lipogenic effect of CBG^45^ and could also suppress the effect of AA (Fig. 7c,d), i.e., the modifications did not have any major impact on their biological activity on human sebocytes.

### Antiviral evaluations (SARS-CoV-2)

CBD and CBG derivatives were further tested for their ability to inhibit SARS-CoV-2 replication in Vero E6 cells. Initially, the inhibition of virus-induced cytopathic effect was measured by addition of twofold serial dilutions of each derivative to the cells, followed by the infection with SARS-CoV-2 and determination of the cell viability 3 days post-infection by XTT assay. Concurrently, the cytotoxicity of each compound was assessed from identical dilutions in uninfected Vero E6 cells. To confirm anti-SARS-CoV-2 activity in the CPE inhibition assay, selected compounds were tested in immunofluorescence assay, where inhibition of SARS-CoV-2 replication was monitored as reduction in nucleoprotein expression using fluorescence microscope. While CBD and CBG were proven to be quite cytotoxic with good antiviral activities, the synthetically modified CBD derivatives, except the disubstituted dietylaminomethyl derivative **13**, had low or no cytotoxic effects, and they showed good activities against SARS-CoV-2 (Table 3). Among the CBG derivatives, we can find quite cytotoxic and non-cytotoxic derivatives as well. While compound **17a** and **20** are non-toxic and they are totally inactive against SARS-CoV-2, the also non-toxic **21b** has good activity against SARS-CoV-2.

**Table 3:** Cytotoxicity and anti-SARS-CoV-2 effect of CBD, CBG, and their derivatives.

Compound	EC_50_ CPE [µM]	EC_50_ IF [µM]	CC_50_ [µM]
**1** (CBD)	5.1 ± 0.99	8.3 ± 0.28	*12.1* ± *1.2*
**8**	**21 ± 3.7**	31 ± 1.1	> 100
**9**	**22 ± 3.0**	~ 94^a^	> 100
**10**	**9.0 ± 0.51**	~ 42^a^	> 100
**11**	**17 ± 1.5**	33 ± 1.4	77 ± 2.6
**12**	**11 ± 1.2**	39 ± 1.4	~ 56^a^
**13**	> 12	n.d.	*12* ± *0.28*
**2** (CBG)	> 11	n.d.	~ *11*^*a*^
**16b**	> 7.7	n.d.	*7.7* ± *3.2*
**17a**	> 100	n.d.	> 100
**18**	15 ± 0.94	> 28	~ *28*^*a*^
**20**	> 100	n.d.	> 100
**21b**	**12 ± 0.99**	> 100	> 100
Remdesivir	2.8 ± 0.18	1.2 ± 0.03	> 100

*n.d.* not determined, *CPE* cytopathic effect-based assay, *IF* immunofluorescence assay.

Values with bold: significant antiviral effect with no or low cytotoxicity. Values with italics: high cytotoxicity.

^a^Approximate value, 95% CI was too wide; therefore, standard error was not computed.

### Antibacterial evaluation

The antibacterial activity of the synthesized compounds against a panel of Gram-positive and Gram-negative bacteria using CBD and CBG as reference was also evaluated. CBD and CBG showed good antibacterial activity with 2–8 μg/ml minimum inhibitory concentration values against Gram-positive strains such as *Bacillus subtilis*, methicillin sensitive *Staphylococcus aureus*, methicillin resistant *Staphylococcus aureus*, biofilm forming *Staphylococcus epidermidis*, mecA gene expressing *Staphylococcus epidermidis* and *Enterococcus faecalis* strains (Supplementary Information, Table S1), whereas most of the synthetically modified derivatives were totally inactive against the investigated bacteria. Only CBD derivatives **8** and **9** have good activity against two *Enterococcus faecalis* strains with 2–8 μg/ml minimum inhibitory concentration values. Compound **8s** has similar activities than its free base derivative **8**. All of the semisynthetic derivatives and the mother compounds CBD and CBG were ineffective against Gram-negative bacteria, no antibacterial effect was observed against *Escherichia coli, Pseudomonas aeruginosa,* multidrug-resistant *Acinetobacter baumannii* and *Klebsiella pneumoniae* carbapenemase-producing bacteria up to 256 μg/ml.

## Discussion

CBD and CBG have poor oral bioavailability because of their low water solubility and extensive first-pass metabolism^52^. By applying appropriate chemical modifications, these disadvantages can be overcome and the biological activities can be increased. In this work, Mannich-type reactions were performed to modify the structure of CBD and CBG using different types of commercially available simple primary and secondary amines and formaldehyde as reagents to examine this type of modifications of cannabinoids for the first time. The reaction of cannabinoids and formaldehyde with primary amines yielded 1,3-benzoxazine derivatives via a Mannich-type aminomethylation followed by cyclization, in line with literature results on Mannich-type reactions of phenols^32–34,53^. After optimization, these cyclocondensation derivatives (**8**–**11**) were formed from CBD with excellent yields (94–100%), only the synthesis of the GABA adduct **12** was moderately efficient. In the case of CBG, the formation of bicyclic benzodioxazines was observed with variable yields (10–71% for **16a**–**19a** and **20**), since mostly a second aminomethylation-cyclocondensation sequence also occurred, resulting in the formation of tricyclic benzodioxazines as main (**16b**) or side products (**17b**–**19b**). Mannich-type reaction with the secondary amine **6** provided mono- and bis-diethylaminomethyl substituted derivatives (**13, 21a** and **21b**) with high yields. From compound **8**, a HCl salt was also produced.

Antineoplastic properties of the produced derivatives along with the parent CBD and CBG were investigated on four human tumorous cell lines including an ovarian cancer (A2780) a cervix carcinoma (SiHA) and two types of breast cancer (MDA-MB-231 and MCF7) cell lines. Both CBD and CBG showed significant antiproliferative activity at 30 μM concentration, although CBD had greater activities, reaching an inhibitory effect of approximately 90% in all 4 cell lines. Mannich-type modifications on CBD significantly reduced the antitumor effect, the only exception was the N-butyl benzoxazine derivative (**8**), which was much more potent than the parent CBD against MCF7 breast cancer cells in lower concentration. However, importantly, two tricyclic CBG derivatives, N-butyl benzodioxazine **16b** and N-allyl benzodioxazine **19b**, showed significant antiproliferative properties against cancer cell lines of breast, ovarian and uterine origin. The data clearly show that the tricyclic benzodioxazine framework is essential for the effect, since the bicyclic and the diethylaminomethylated CBGs, similarly to the analogous CBD derivatives, were completely ineffective. At the same time, the ineffectiveness of the N-benzylated tricyclic CBG derivative **17b** shows that the substituent of the amino groups is also important for antiproliferative activity, and that alkyl substituents are more favorable than aryl-containing ones. Considering the highly promising antiproliferative effect of **16b** and **19b** against all tested cell lines, cannabinoid-based benzodioxazines as a new potential antitumor pharmacophores are worthy of further investigation. In this context, we plan to produce analogous tricyclic derivatives from CBD to clarify whether the CBG backbone itself, i.e. the geranyl side chain, is essential for the effect.

As mentioned above, topically applied synthetic CBD showed promising efficiency in a placebo-controlled phase 2 clinical trial in moderate to severe acne (ClinicalTrials.gov ID: NCT03573518), and is about to be assessed in a placebo-controlled phase 3 clinical trial^19^. Among the synthetically modified cannabinoids, two CBD (**8** and **9**) and two CBG (**16a** and **21b**) derivatives were selected for anti-acne testing. Obviously, within the confines of the current study, we could not perform complete “acne-relevant” profiling of the test compounds (e.g., assessment of the effects on the proliferation as well as on the immune phenotype and the qualitative lipidome of the sebocytes). However, because unlike the respective concentrations of CBD, 10–30 μM of compounds **8** and **9** increased (or at least did not decrease) sebaceous lipogenesis, while they preserved CBD’s ability to suppress AA-induced, “acne-mimicking” lipid synthesis (Fig. 7a,b), the new compounds may become particularly interesting anti-acne drug candidates, although further studies are needed.

**Figure 8:**
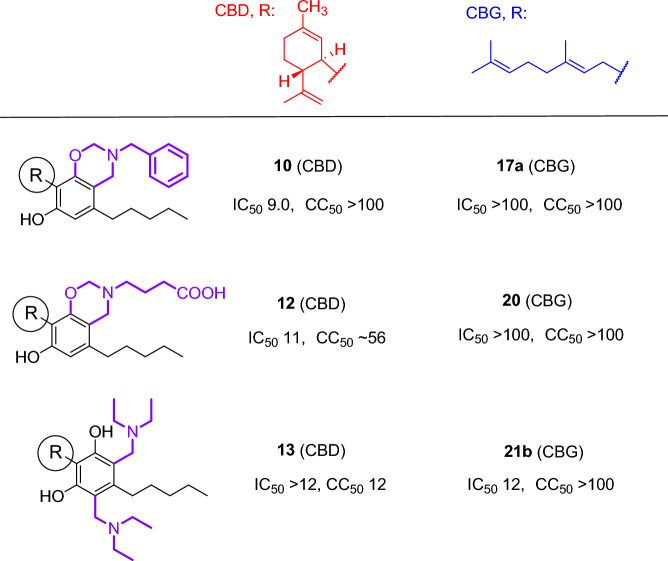
Anti-SARS-CoV-2 activity and citotoxicity of equally modified CBD and CBG pairs.

Recently, it was published that CBD can reduce severe acute respiratory syndrome coronavirus-2 (SARS-CoV-2) viral infection either by downregulating ACE2 transcript levels^54^ or by blocking the viral main protease (Mpro)^55^. According to our results, CBD and CBG are indeed active against the new coronavirus; however, in the active concentration range they also exert significant cytotoxic effect. Remarkably, all of the 1,3-oxazine-condensed CBDs **8**, **10**, **11** and **12,** independently of the substituent of the amino group, were highly active against SARS-CoV-2 with low or no cytotoxicity (Table 3). It indicates that the Mannich-type modification of CBD with primary amines was highly beneficial to the selective antiviral activity. Among the modified CBD derivatives, only the bis-diethyalminomethyl derivative **13** did not show anti-SARS-CoV-2 activity. In contrast, among the CBG derivatives, only the bis-diethyalminomethyl derivative **21b** displayed a strong and selective antiviral effect (Fig. 8). These data demonstrate that although CBD is much more promising as a potential antiviral pharmacophore, an active derivative can also be obtained from CBG by appropriate modification. To compare the contribution of the cannabinoid backbone and the substituents to the anti-SARS-CoV-2 effect, the antiviral assay data of the identically modified CBD and CBG pairs are summarized in Fig. 8. The selective effect of CBD and CBG derivatives against SARS-CoV-2 is quite remarkable and worthy of further investigation. In addition, CBD and its derivatives may have other properties that can help in the treatment of COVID-19 such as anti-inflammatory activity^56^, and ability to induce the host innate immune responses^57^, making this class of compounds attractive for further antiviral development.

The antibacterial properties of the new derivatives were also evaluated using CBD and CBG as reference compounds. CBD and CBG have good activities against the investigated bacterial strains, while the derivatives (except for **8**, **8s** and **9** against two types of *Enterococcus faecalis*) completely lost the antibacterial activity due to the synthetic modification.

As a conclusion, using Mannich-type reaction, we prepared a small compound library of amine-containing derivatives of CBD and CBG. After optimizing the reaction conditions, completely new bi- and tricyclic cannabinoids with benzoxazine and benzodioxazine rings and mono- and bis-aminomethyl-substituted derivatives were produced in good yields. For the biological profiling of the new amine-containing cannabinoids, in vitro antibacterial, anti-acne, anti-cancer and anti-viral tests were performed, which yielded mixed results. The modifications impaired the antibacterial effect of the parent CBD and CBG, while not significantly altering the anti-acne activity. At the same time, two tricyclic benzodioxazine CBG derivatives (**16b** and **19b**) showed strong antiproliferative effects on all four tested human tumor cell lines, thus the tricyclic cannabinoid structure was identified as a very promising new antitumor pharmacophore. Our most important finding is the remarkable anti-SARS-CoV-2 activity of benzoxazine CBD derivatives. The modified derivatives inhibited the replication of SARS-CoV-2 with an EC_50_ value similar to that of CBD, while, importantly, their cytotoxicity significantly decreased compared to the parent compound. Furthermore, we demonstrated that the preparation of HCl salt from compound **8** did not affect the biological effect of the basic skeleton, but it may play a significant role in improving the in vivo bioavailability. However the number of the produced derivatives does not allow us to establish precise structure–activity relationships, some promising structures and modification directions have been identified. Benzoxazine CBDs as potential antiviral agents and benzodioxazine tricyclic cannabinoids as potential antitumor agents deserve further investigation, but additional derivatives with additional different amines should be prepared for more detailed studies.

## Methods

### Cell culturing

Human immortalized HaCaT keratinocytes^49^ were purchased from Antibody Research Corporation (Saint Charles, MO, USA), and were cultured in Dulbecco’s Modified Eagle Medium (DMEM; Cat. No. 31966-021; Gibco-Thermo Fisher Scientific) supplemented with 10% heat inactivated fetal bovine serum (FBS; Cat. No. 10500-064; Gibco-Thermo Fisher Scientific), and antibiotics (MycoZap™ Plus-CL in 1:500; Cat. No. VZA-2011; Lonza, Basel, Switzerland) at 37 °C in a 5% CO_2_-containing humidified atmosphere. The medium was changed every other day, and cells were sub-cultured at 70–80% confluence.

Human immortalized SZ95 sebocytes, originated from human facial sebaceous glands^50^ were provided by prof. Christos C. Zouboulis, and were cultured in Sebomed^®^ Basal Medium (Cat. No. F8205; Sigma-Aldrich) supplemented with 10% FBS (Gibco-Thermo Fisher Scientific), 1 mM CaCl_2_ (Cat. No. C7902; Sigma-Aldrich), 5 ng/ml human epidermal growth factor (Cat. No. E9644; Sigma-Aldrich), and MycoZap™ Plus-CL (1:500; Lonza). The medium was changed every other day, and cells were subcultured at 60–70% confluence.

Both HaCaT keratinocytes and SZ95 sebocytes were regularly checked for *Mycoplasma* contamination by using MycoAlert™ PLUS Mycoplasma Detection Kit (Cat. No. LT07-710; Lonza), and every assessment yielded negative result.

Human adherent cancer cell lines A2780, MDA-MB-231 and MCF-7 were obtained from ECACC (European Collection of Cell Cultures, Salisbury, UK; Cat. No.93112519, 92020424, 86012803, respectively). SiHa cell line was obtained from ATCC (American Tissue Culture Collection, Manassas, Virginia, USA; Cat. No. HTB-35). All cell lines used for antiproliferative assay were maintained in Eagle's Minimum Essential Medium (EMEM) completed with 10% fetal bovine serum (FBS), 1% non-essential amino acid solution, and 1% penicillin, streptomycin, and amphotericin B mixture under cell culture conditions described before. All the utilized media and supplements were purchased from Lonza Group Ltd. (Basel, Switzerland).

### Determination of intracellular lipids^58^

For quantitative measurement of sebaceous (neutral) lipid content, SZ95 sebocytes (20,000 cells/well) were cultured in 96-well “black well/clear bottom” plates (Cat. No. 655090; Greiner Bio-One, Frickenhausen, Germany), and were treated with compounds in the presence or absence of arachidonic acid (AA; Cat. No. 90010; Cayman Chemical Company, Ann Arbor, MI, USA) or its vehicle (absolute ethanol) as indicated. Subsequently, supernatants were discarded, cells were washed twice with phosphate-buffered saline (PBS; 115 mM NaCl, 20 mM Na_2_HPO_4_, pH 7.4; all from Sigma-Aldrich), and 100 µl of a 1 µg/ml Nile Red (Cat. No. N3013; Sigma-Aldrich) solution in PBS was added to each well. The plates were then incubated at 37 °C for 30 min, and fluorescence was measured on FlexStation 3 multimode microplate reader (Molecular Devices, San Francisco, CA, USA). Results, measured in relative fluorescence units, are expressed as percentage of the vehicle (absolute ethanol; Cat. No. 20821.296*;* VWR International)-treated control regarded as 100%, using 485 nm excitation and 565 nm emission wavelengths.

### Determination of cellular viability

The viability of HaCaT keratinocytes and SZ95 sebocytes was determined by MTT-assay^58^ (Cat. No. M5655; Sigma-Aldrich) measuring the conversion of the tetrazolium salt to formazan by mitochondrial dehydrogenases. Cells were plated in 96-well plates (20,000 cells/well), and were treated as indicated. Cells were then incubated with 0.5 mg/ml MTT reagent for 3 h, and concentration of formazan crystals (as an indicator of number of viable cells) was determined colorimetrically at 565 nm by using FlexStation 3 multi-mode microplate reader (Molecular Devices). Results were expressed as percentage of vehicle-treated control regarded as 100%.

The antiproliferative actions of the prepared molecules were determined by the same assay against a panel of adherent human cancer cell lines containing cervical (SiHa), ovarian (A2780) and breast (MCF7, MDA-MB-231) cell lines. Cells were plated onto 96-well microplates (5000 cells/well) and after an overnight incubation treated with two concentrations (10 or 30 μM) of the test substances for 72 h under cell culture conditions. Subsequently, MTT (5 mg/ml in phosphate buffer) was added to the wells, followed by incubation for 4 h. The supernatant was removed, the formazan crystals were then dissolved in 100 µl DMSO. Finally, absorbance values were determined by a microplate reader (BMG Labtech, Ortenberg, Germany) at 545 nm. Two independent measurements were performed with five parallel wells. Results were expressed as percentage of cell proliferation inhibition compared to non-treated control samples regarded as 0%.

### Statistical analysis in case of MTT and Nile Red assays

Data were analyzed by GraphPad Prism 9.5.0 (730) (GraphPad Software, LLC, San Diego, CA, USA). Depending on the sample size, Gaussian distribution was tested by D’Agostino & Pearson test. In case of Gaussian distribution, one-way ANOVA followed by Šídák’s multiple comparisons test was used, whereas in case of non-Gaussian distribution, Kruskal–Wallis test followed by Dunn’s multiple comparisons test was used, and *P* < 0.05 values were regarded as significant differences. Graphs were plotted using GraphPad Prism 9.5.0 (730) (GraphPad Software, LLC).

### Anti-SARS-CoV-2 testing

The anti-SARS-CoV-2 activity was measured by determining the extent to which the test compounds inhibited virus-induced cytopathic effect (CPE) and SARS-CoV-2 replication detected by immunofluorescence staining of SARS-CoV-2 nucleoprotein expression in Vero E6 (ATCC, cat. no. CRL-1586). For CPE-based assay, two-fold fold serial dilutions of compounds from 100 µM were added in triplicate in a 384-well plate with 5000 Vero E6 cells in DMEM medium with 2% FBS, 100 U of penicillin/ml and 100 µg of streptomycin/ml (all Merck). After 1 h incubation SARS-CoV-2 (strain hCoV-19/Czech Republic/NRL_6632_2/2020) was added at multiplicity of infection 0.1 IU/ml. Following three days incubation at 37 °C in 5% CO_2_ the cell viability was determined by addition of XTT solution (Sigma-Aldrich) for 4 h and the absorbance was measured using EnVision plate reader (Perkin Elmer). Drug concentrations required to reduce viral cytopathic effect by 50% (EC_50_) were calculated using nonlinear regression from plots of percentage cell viability versus log10 drug concentration using GraphPad Prism (version 9.0.0, GraphPad Software, LLC). For immunofluorescence assay, two-fold fold serial dilutions of compounds from 100 µM were added in triplicate in 96-well plate with 15,000 Vero E6 cells plated day before in the same medium as above. After 1 h incubation, SARS-CoV-2 was added at multiplicity of infection 0.05 IU/cell. After three days of incubation, cells were fixed with 4% paraformaldehyde, permeabilized with 0.2% Triton X100 (both Merck), washed, incubated with anti-SARS-CoV-2antibody (mouse monoclonal antibody detecting SARS-CoV-2 nucleoprotein) for 2 h at room temperature. Followed by 1.5 h incubation with Cy3-labeled donkey anti-mouse IgG (Jackson ImmunoResearch Europe) and documented using fluorescence microscope with camera (Olympus). Images were processed in ImageJ software (NIH) and compound concentrations required to reduce fluorescence by 50% (EC_50_s) were calculated from plots of percentage of fluorescent cells versus log_10_ drug concentration using nonlinear regression analysis as above. Cytotoxicity was evaluated by incubating with the same two-fold serial dilutions of each compound as above with Vero E6 cells in 384-well plate. Following three days incubation at 37 °C in 5% CO_2_, the cell viability was determined by addition of XTT solution, and the compound concentrations resulting in 50% reduction of absorbance i.e., 50% reduction of cell viability (CC_50_), were calculated as above in the CPE-based assay.

### Antibacterial efficacy assay^59^

According to CLSI guideline the Minimal Inhibitory Concentrations (MICs) of the prepared compounds were determined with the broth micro dilution method. After an overnight growth on 5% bovine blood agar plates at 35 °C bacterial strains were suspended in physiological saline in order to reach the density of 0.5 McFarland for inoculation. Stock solutions containing different concentrations of the substances were prepared in DMSO and H_2_O (1:1). These were twofold serially diluted from 256 to 0.5 mg/l in Mueller–Hinton broth, and 100 ml of each dilution was inoculated with 10 ml of each bacterial suspension. Incubation was performed at 35 °C for 18 h and determination of MIC was made with the naked eyes on a dark background.

### Chemical synthesis

#### General information

CBD and CBG was purchased from CBDepot.eu. Compound **8** (*Reaction route A1*) and **13** (*Reaction route E*) were described by Szőke et al.^37^.

Preparation of TLC, column chromatography, recording of NMR spectra and MS measurements were carried out based on the previously described methods^60^. TLC was performed on Kieselgel 60 F254 (Merck) with detection by immersing into ammonium molybdate-sulfuric acid solution followed by heating. Flash column chromatography was performed using Silica gel 60 (Merck 0.040–0.063 mm).

^1^H NMR (500 and 400 MHz), ^13^C NMR (125 and 100 MHz) and 2D NMR spectra were recorded with a Bruker DRX-400 and Bruker Avance II 500 spectrometer at 298 K, 300 K or 305 K. Chemical shifts are referenced to Me_4_Si (0.00 ppm for 1H) and to the solvent residual signals. Numbering of CBD, CBG and their derivatives can be found in Supplementary Information (Figs. S2 and S3). NMR spectra and data of compound **8**, **9** and **13** were described by Szőke et al.^37^.

ESI-QTOF MS measurements were carried out by two different instruments. In the case of maXis II UHR ESI-QTOF MS instrument (Bruker), the following parameters were applied for the electrospray ion source in positive ionization mode: capillary voltage: 3.5 kV; end plate offset: 500 V; nebulizer pressure: 0.8 bar; dry gas temperature: 200 °C and dry gas flow rate: 4.5 l/min. Constant background correction was applied for each spectrum, the background was recorded before each sample by injecting the blank sample matrix (solvent). Na-formate calibrant was injected after each sample, which enabled internal calibration during data evaluation. Mass spectra were recorded by otofControl version 4.1 (build: 3.5, Bruker) and processed by Compass DataAnalysis version 4.4 (build: 200.55.2969). ESI-QqTOF MS spectra were recorded by a microTOF-Q type QqTOFMS mass spectrometer (Bruker, Germany) equipped with an electrospray ion source in negative or positive ion mode using MeOH as solvent. The mass spectrometer was operated in positive ion mode with a capillary voltage of 3.5 kV, an endplate offset of − 500 V, nebulizer pressure of 1.8 bar, and N_2_ as drying gas with a flow rate of 9.0 l/min at 200 °C. The mass spectra were recorded by means of a digitizer at a sampling rate of 2 GHz. The mass spectra were calibrated externally using the exact masses of clusters [(NaTFA)_n_ + TFA]^+^ from the solution of sodium trifluoroacetate (NaTFA). The spectra were evaluated with the DataAnalysis 3.4 software from Bruker. MALDI-TOF MS measurement was carried out with a Bruker Autoflex Speed mass spectrometer equipped with a time-of-flight (TOF) mass analyzer. In this case, 19 kV as ion source voltage 1 and 16.65 kV as ion source voltage 2 were used. For reflectron mode, 21 kV and 9.55 kV were applied as reflector voltage 1 and reflector voltage 2, respectively. A solid phase laser (355 nm, ≥ 100 μJ/pulse) operating at 500 Hz was applied to produce laser desorption and 3000 shots were summed. 2,5-Dihydroxybenzoic acid (DHB) was used as matrix and F_3_CCOONa as cationising agent in DMF.

#### Compound **8**

Formaldehyde (36% in water, 125 μl, 1.5 mmol) and *n*-butylamine (**3**) (148 μl, 1.5 mmol) were dissolved in a solvent (10 ml). The mixture was stirred for 20 min then cannabidiol (157 mg, 0.5 mmol) was added. The mixture was stirred for a specified time at a given temperature, then the solvent was evaporated and the residue was purified by flash column chromatography (hexane/acetone 98:2).

***Reaction route A1:***^37^ The solvent was methanol. The prepared mixture was stirred at room temperature for 7 days. Compound **8** was yielded (188 mg, 89%) as a yellowish syrup.

***Reaction route A2:*** The solvent was ethanol. The reaction mixture was stirred at reflux temperature for 20 h. Compound **8** was yielded (132 mg, 64%) as a yellowish syrup and compound **9** was yielded as a byproduct (39 mg, 21%) as a yellowish syrup.

***Reaction route A3:*** The solvent was ethanol. The reaction mixture was stirred at reflux temperature for 6 h and then 45 °C for 18 h. Compound **8** was yielded (205 mg, 100%) as a yellowish syrup.

***Reaction route A4:*** The solvent was dioxane. The reaction mixture was stirred at reflux temperature for 6 h and then 50 °C for 18 h. Compound **8** was yielded (123 mg, 60%) as a yellowish syrup.

*R*_f_ = 0.47 (hexane/acetone 95:5); ^1^H NMR (500 MHz, CDCl_3_): *δ* (ppm) 6.25 (s, 1H, aromatic C*H*), 5.90 (s, 1H, O*H*), 5.58 (s, 1H, C-2 C*H*), 4.76 (d, 1H, *J* = 9.6 Hz, H-B C*H*_2_a), 4.66 (dd, 1H, *J* = 9.6 and 1.2 Hz, H-B C*H*_2_b), 4.47 (dd, 1H, *J* = 2.5 and 1.4 Hz, H-9 C*H*_2_a), 4.33 (d, 1H, *J* = 2.3 Hz, H-9 C*H*_2_b), 3.97–3.90 (m, 2H, H-3 C*H* and H-A C*H*_2_a), 3.81–3.74 (m, 1H, H-A C*H*_2_b), 2.71–2.57 (m, 2H, butyl C*H*_2_), 2.43–2.30 (m, 3H, H-1″ C*H*_2_ and H-4 C*H*), 2.27–2.17 (m, 1H, H-6 C*H*_2_a), 2.11–2.03 (m, 1H, H-6 C*H*_2_b), 1.81–1.72 (m, 5H, H-5 C*H*_2_ and H-7 C*H*_3_), 1.66 (s, 3H, H-10 C*H*_3_), 1.56–1.47 (m, 4H, H-2″ and butyl C*H*_2_), 1.40–1.28 (m, 6H, H-3″, H-4″ and butyl C*H*_2_), 0.93 (t, 3H,* J* = 7.3 Hz, H-5″ C*H*_3_), 0.91–0.86 (m, 3H, butyl C*H*_3_); ^13^C NMR (125 MHz, CDCl_3_): *δ* (ppm) 154.0, 152.5, 147.5, 139.7, 139.6 (5C, quat.), 124.7 (1C, C-2 *C*H), 114.4 (1C, quat.), 111.2 (1C, C-9 *C*H_2_), 109.4 (1C, quat.), 109.2 (1C, aromatic *C*H), 81.6 (1C, C-B *C*H_2_), 51.2 (1C, butyl *C*H_2_), 48.6 (1C, C-A *C*H_2_), 47.0 (1C, C-4 *C*H), 35.3 (1C, C-3, *C*H), 31.90, 39.88, 30.6, 30.4, 29.8 (5C, C-1″, C-2″, C-6 and 2xbutyl *C*H_2_), 28.3 (1C, C-5 *C*H_2_), 23.8 (1C, C-7 *C*H_3_), 22.7, 20.7 (2C, C-4″ and butyl *C*H_2_), 18.8 (1C, C-10 *C*H_3_), 14.2 (2C, C-5″ and butyl *C*H_3_); ESI-QqTOF MS: *m/z* calcd for C_27_H_41_NO_2_Na^+^: 434.303 [M+Na]^+^; found: 434.300.

#### Salt formation of compound **8** (**8s**)

Compound **8** (205 mg, 0.5 mmol) was dissolved in anhydrous 1,4-dioxane (10 ml) and the solvent was cooled in icebath. Concentrated H_2_SO_4_ was added dropwise to solid NaCl, and the produced HCl gas was introduced into the prepared solution of compound **8**. The corresponding HCl salt was precipitated from the solution, the precipitate was filtered off, washed with cold dioxane and dried under vacuo to yield **8s** (223 mg, 100%) as a white powder.

#### Compound **9**

Cannabidiol (157 mg, 0.5 mmol) was dissolved in ethanol (10 ml) then formaldehyde (36% in water, 125 μl, 1.5 mmol) and triethylamine (209 μl, 1.5 mmol) were added. The mixture was stirred for 48 h at reflux temperature during the days (2 × 8 h) and 45 °C for overnights (2 × 16 h). The solvent was evaporated and the residue was purified by flash column chromatography (hexane/acetone 99:1) to yield **9** (55 mg, 30%) as a yellowish syrup.

*R*_f_ = 0.69 (hexane/acetone 9:1); ^1^H NMR (400 MHz, CDCl_3_): *δ* (ppm) 8.02 (s, 1H, O*H*), 6.20 (s, 1H, aromatic C*H*), 6.00 (s, 1H, O*H*), 5.59 (s, 1H, H-2 C*H*), 4.72–4.58 (m, 2H, H-A C*H*_2_), 4.51 (s, 1H, H-9 C*H*_2_a), 4.40 (s, 1H, H-9 C*H*_2_b), 4.09–3.98 (m, 1H, H-3 C*H*), 3.57–3.43 (m, 2H, ethyl C*H*_2_), 2.36–2.53 (m, 3H, H-4 C*H* and H-1″ C*H*_2_), 2.29–2.16 (m, 1H, H-6 C*H*_2_a), 2.12–2.02 (m, 1H, H-6 C*H*_2_b), 1.84–1.74 (m, 5H, H-5 C*H*_2_ and H-7 C*H*_3_), 1.69 (s, 3H, H-10 C*H*_3_), 1.51–1.41 (m, 2H, H-2″ C*H*_2_), 1.37–1.27 (m, 4H, H-3″ and H-4″ C*H*_2_), 1.23 (t, 3H,* J* = 7.0 Hz, ethyl C*H*_3_), 0.92–0.84 (m, 3H, H-5″ C*H*_3_); ^13^C NMR (100 MHz, CDCl_3_): *δ* (ppm) 155.9, 155.7, 147.9, 140.0 (5C, quat.), 124.7 (1C, C-2 *C*H), 115.1 (1C, quat.), 111.0 (1C, C-9 *C*H_2_), 109.3 (1C, aromatic *C*H), 108.1 (1C, quat), 67.8 (1C, C-B *C*H_2_), 65.3 (2C, ethyl *C*H_2_), 46.7 (1C, C-4 *C*H), 35.8 (1C, C-3, *C*H), 33.3 (1C, C-1″ *C*H_2_), 31.8 (1C, C-3″ *C*H_2_), 31.2 (1C, C-2″ *C*H_2_), 30.4 (1C, C-6, *C*H_2_), 28.1 (1C, C-5 *C*H_2_), 23.8 (1C, C-7, *C*H_3_), 22.7 (1C, C-4″ *C*H_2_), 19.3 (1C, C-10 *C*H_3_), 15.2 (1C, ethyl *C*H_3_), 14.2 (1C, C-5″ *C*H_3_); ESI-QqTOF MS: *m/z* calcd for C_24_H_35_O_3_^−^: 371.258 [M−H]^−^; found: 371.255.

#### Compound **10**

Formaldehyde (36% in water, 250 μl, 3 mmol) and benzylamine (**4**) (328 μl, 3 mmol) were dissolved in dioxane (10 ml). The mixture was stirred for 20 min then cannabidiol (314 mg, 1 mmol) was added. In ***reaction route B1*** the mixture was stirred at reflux temperature for 6 h and then 70 °C for 18 h. The solvent was evaporated and the residue was purified by flash column chromatography (hexane/acetone 99:1) to yield **10** (214 mg, 48%) as a yellowish syrup. In ***reaction route B2*** the mixture was stirred at 50 °C for 9 days. The solvent was evaporated and the residue was purified by flash column chromatography (hexane/acetone 99:1) to yield **10** (418 mg, 94%) as a yellowish syrup.

*R*_f_ = 0.42 (hexane/acetone 98:2); ^1^H NMR (400 MHz, CDCl_3_): *δ* (ppm) 7.38–7.22 (m, 5H, benzyl C*H*), 6.28 (s, 1H, H-4′ aromatic C*H*), 5.94 (s, 1H, O*H*), 5.61 (s, 1H, H-2 C*H*), 4.80 (d, 1H, *J* = 9.6 Hz, H-B C*H*_2_a), 4.68 (dd, 1H, *J* = 9.7 and 1.2 Hz, H-B C*H*_2_b), 4.56 (dt, 1H, *J* = 2.5 and 1.4 Hz, H-9 C*H*_2_a), 4.39 (d, 1H, *J* = 2.4 Hz, H-9 C*H*_2_b), 4.01–3.93 (m, 1H, H-3 C*H*), 3.93–3.68 (m, 4H, H-A and benzyl C*H*_2_), 2.48–2.38 (m, 1H, H-4 C*H*), 2.31–2.18 (m, 3H, H-6 C*H*_2_a and H-1″ C*H*_2_), 2.13–2.03 (m, 1H, H-6 C*H*_2_b), 1.83–1.73 (m, 5H, H-5 C*H*_2_ and H-7 C*H*_3_), 1.71 (s, 3H, H-10 C*H*_3_), 1.49–1.40 (m, 2H, H-2″ C*H*_2_), 1.32–1.21 (m, 4H, H-3″ and H-4″ C*H*_2_), 0.91–0.82 (m, 3H, H-5″ C*H*_3_); ^13^C NMR (100 MHz, CDCl_3_): *δ* (ppm) 154.2, 152.3, 147.6, 139.9, 139.6, 138.7 (6C, quat.), 129.1, 128.5, 127.4 (5C, 5xbenzyl *C*H), 124.6 (1C, C-2 *C*H), 114.5 (1C, quat.), 111.2 (1C, C-9 *C*H_2_), 109.5 (1C, C-4′ *C*H), 109.1 (1C, quat.), 81.8(1C, C-B, *C*H_2_), 55.5, 47.7 (2C, C-A and benzyl *C*H_2_), 47.0 (1C, C-4 *C*H), 35.4 (1C, C-3 *C*H), 31.8 (2C, C-3″ and C-1″ *C*H_2_), 30.5 (1C, C-6 *C*H_2_), 29.7 (1C, C-2″ *C*H_2_), 28.3 (1C, C-5 *C*H_2_), 23.8 (1C, C-7 *C*H_3_), 22.7 (1C, C-4″ *C*H_2_), 19.0 (1C, C-10 *C*H_3_), 14.1 (1C, C-5″ *C*H_3_); UHR ESI-QTOF MS: *m/z* calcd for C_30_H_39_NO_2_H^+^: 446.3054 [M+H]^+^; found: 446.3054.

#### Compound **11**

Formaldehyde (36% in water, 250 μl, 5 mmol) and propargylamine (**5**) (328 μl, 5 mmol) were dissolved in dioxane (10 ml). The mixture was stirred for 20 min then cannabidiol (314 mg, 1 mmol) was added. In ***reaction route C1*** the mixture was stirred at reflux temperature for 48 h and at 80 °C for 3 days, then the solvent was evaporated and the residue was purified by flash column chromatography (hexane/acetone 99:1) to yield **11** (205 mg, 46%) as a yellowish syrup. In ***reaction route C2*** the mixture was stirred at 50 °C for 9 days, then the solvent was evaporated and the residue was purified by flash column chromatography (hexane/acetone 99:1) to yield **11** (418 mg, 94%) as a yellowish syrup.

*R*_f_ = 0.52 (hexane/acetone 8:2); ^1^H NMR (400 MHz, CDCl_3_): *δ* (ppm) 6.27 (s, 1H, aromatic C*H*), 5.96 (s, 1H, O*H*), 5.57 (s, 1H, H-2 C*H*), 4.82–4.73 (m, 2H, H-B C*H*_2_), 4.49 (t, 1H, *J* = 2.0 Hz, H-9 C*H*_2_a), 4.30 (s, 1H, H-9 C*H*_2_b), 4.02 (d, 1H, *J* = 16.3 Hz, H-A C*H*_*2*_a), 3.98–3.86 (m, 2H, H-3 C*H* and H-A C*H*_*2*_b), 3.55–3.39 (m, 2H, propargyl C*H*_2_), 2.31–2.43 (m, 3H, H-1″ C*H*_2_ and H-4 C*H*), 2.28 (t, 1H, *J* = 2.5 Hz, propargyl C*H*), 2.26–2.17 (m, 1H, H-6 C*H*_2_a), 2.11–2.02 (m, 1H, H-6 C*H*_2_b), 1.83–1.72 (m, 5H, H-5 C*H*_2_ and H-7 C*H*_3_), 1.68 (s, 3H, H-10 C*H*_3_), 1.58–1.45 (m, 2H, H-2″ C*H*_3_), 1.38–1.25 (m, 4H, H-3″ and H-4″ C*H*_2_), 0.94–0.84 (m, 3H, H-5″ C*H*_3_); ^13^C NMR (100 MHz, CDCl_3_): *δ* (ppm) 154.1, 151.7, 147.4, 139.7, 139.6 (5C, quat.), 124.4 (1C, C-2 *C*H), 114.4 (1C, quat.), 111.1 (1C, C-9 *C*H_2_), 109.6 (1C, aromatic *C*H), 108.2 (1C, quat.), 80.6 (1C, C-B *C*H_2_), 80.2 (1C, propargyl quat), 72.6 (1C, propargyl *C*H), 47.9 (1C, C-A *C*H_2_), 47.0 (1C, C-4 *C*H), 40.6 (1C, propargyl *C*H_2_), 35.2 (1C, C-3 *C*H), 31.8 (2C, C-1″ and C-3″ *C*H_2_), 30.4 (1C, C-6 *C*H_2_), 29.6 (1C, C-2″ *C*H_2_), 28.1 (2C, C-5 *C*H_2_), 23.7 (1C, C-7 *C*H_3_), 23.0 (1C, C-4″ *C*H_2_), 18.5 (1C, C-10 *C*H_3_), 14.1 (1C, C-5″ *C*H_3_); UHR ESI-QTOF MS: *m/z* calcd for C_26_H_35_NO_2_Na^+^: 416.2560 [M+Na]^+^; found: 416.2557.

#### Compund **12**

Formaldehyde (36% in water, 250 μl, 3 mmol) and gamma-aminobutyric acid (**6**) (309 mg, 3 mmol) was dissolved in dioxane (10 ml) and water (1 ml). The mixture was stirred for 20 min then cannabidiol (314 mg, 1 mmol) was added. The mixture was stirred for 4 days at reflux temperature during the days and 70 °C at nights, then the solvent was evaporated and the residue was purified by flash column chromatography (hexane/acetone 8:2) to yield **12** (141 mg, 32%) as a yellowish syrup.

*R*_f_ = 0.24 (hexane/acetone 9:3); ^1^H NMR (400 MHz, CDCl_3_): *δ* (ppm) 6.27 (s, 1H, aromatic C*H*), 5.57 (s, 1H, H-2 C*H*), 4.74 (d, 1H, *J* = 9.7 Hz, H-B C*H*_2_a), 4.65 (d, 1H, *J* = 9.8 Hz, H-B C*H*_2_b), 4.46 (s, 1H, H-9 C*H*_2_a), 4.30 (s, 1H, H-9 C*H*_2_b), 3.98–3.87 (m, 2H, H-3 C*H* and H-A C*H*_2_a), 3.81 (d, 1H,* J* = 16.3 Hz, H-A C*H*_2_b), 2.81–2.67 (m, 2H, GABA C*H*_2_), 2.48–2.29 (m, 5H, H-1″ C*H*_2_, H-4 C*H* and GABA C*H*_2_), 2.29–2.16 (m, 1H, H-6 C*H*_2_a), 2.13–2.02 (m, 1H, H-6 C*H*_2_b), 1.88 (p, 2H, *J* = 7.1 Hz, GABA C*H*_2_), 1.83–1.72 (m, 5H, H-5 C*H*_2_ and H-7 C*H*_3_), 1.65 (s, 3H, H-10 C*H*_3_), 1.56–1.45 (m, 2H, H-2″ C*H*_2_), 1.39–1.29 (m, 4H, H-3″ and H-4″ C*H*_2_), 0.94–0.82 (m, 3H, H-5″ C*H*_3_); ^13^C NMR (100 MHz, CDCl_3_): *δ* (ppm) 177.7 (1C, *C* = O), 154.2, 152.0, 147.3, 139.7 (4C, quat.), 124.4 (1C, C-2 *C*H), 114.5 (1C, quat.), 111.2 (1C, C-9 *C*H_2_), 109.6 (1C, aromatic *C*H), 108.5 (1C, quat.), 81.0 (1C, C-B *C*H_2_), 50.7 (1C, GABA *C*H_2_), 48.0 (1C, C-A, *C*H_2_), 46.9 (1C, C-4 *C*H), 35.3 (1C, C-3 *C*H), 32.6 (1C, GABA *C*H_2_), 31.80, 31.76 (2C, C-1″ and C-3″ *C*H_2_) 30.5 (1C, C-6 *C*H_2_), 29.6 (1C, C-2″ *C*H_2_), 28.1 (1C, C-5 *C*H_2_), 23.8 (1C, C-7 *C*H_3_), 22.7, 22.6 (2C, C-4″ and GABA *C*H_2_), 18.7 (1C, C-10 *C*H_3_), 14.1 (1C, C-5″ *C*H_3_); UHR ESI-QTOF MS: *m/z* calcd for C_27_H_39_NO_4_H^+^: 442.2952 [M+H]^+^; found: 442.2951.

#### Compound **13**

Formaldehyde (36% in water, 250 μl, 5 mmol) and diethylamine (**7**) (310 μl, 3 mmol) was dissolved in dioxane (10 ml). The mixture was stirred for 20 min then cannabidiol (314 mg, 1 mmol) was added. The mixture was stirred at reflux temperature for 6 h and then at 70 °C for 18 h. The solvent was evaporated and the residue was purified by flash column chromatography (hexane/ethyl acetate 9:1) to yield **13** (325 mg, 67%) as a yellowish syrup.

*R*_f_ = 0.56 (hexane/acetone 9:1); ^1^H NMR (500 MHz, CDCl_3_): *δ* (ppm) 5.36 (s, 1H, H-2 C*H*), 4.52 (d, 1H, *J* = 2.9 Hz, H-9 C*H*_2_a), 4.42–4.39 (m, 1H, H-9 C*H*_2_b), 4.06–3.96 (m, 1H, H-3 C*H*), 3.75–3.58 (m, 4H, H-A C*H*_2_), 3.20–3.12 (m, 1H, H-4 C*H*), 2.68–2.45 (m, 8H, ethyl C*H*_2_), 2.44–2.38 (m, 2H, H-1″ C*H*_2_), 2.32–2.22 (m, 1H, H-6 C*H*_2_a), 2.01–1.94 (m, 1H, H-6 C*H*_2_b), 1.73–1.80 (m, 2H, H-5 C*H*_2_), 1.68 (s, 3H, H-7 C*H*_3_), 1.63 (s, 3H, H-10 C*H*_3_), 1.38–1.30 (m, 6H, H-2″, H-3″ and H-4″ C*H*_2_), 1.08 (t, 12H,* J* = 7.2 Hz, ethyl C*H*_3_), 0.94–0.89 (m, 3H, H-5″ C*H*_3_); ^13^C NMR (125 MHz, CDCl_3_): *δ* (ppm) 150.6, 136.6, 130.8, 116.5, 110.3 (8C, quat.), 127.1 (1C, C-2 *C*H), 109.0 (1C, C-9 *C*H_2_), 52.9 (2C, 2 × C-B *C*H_2_), 45.9, 45.8, 45.7 (4C, 4 × ethyl *C*H_2_), 44.4 (1C, C-4 *C*H), 36.6 (1C, C-3 *C*H), 32.2, 30.9, 30.6, 30.1, 29.0, (5C, C-1″, C-6, C-5, C-2″ and C-3″ *C*H_2_), 23.6 (1C, C-7 *C*H_3_), 22.6 (1C, C-4″ *C*H_2_), 19.5 (1C, C-10 *C*H_3_), 14.2 (1C, C-5″ *C*H_3_), 11.6, 11.4, 11.4, 11.3 (4C, 4 × ethyl *C*H_3_). ESI-QqTOF MS: *m/z* calcd for C_31_H_51_N_2_O_2_^−^: 483.396 [M−H]^−^; found: 483.396.

#### Compound **14**

Cannabidiol (157 mg, 0.5 mmol) was dissolved in ethanol (10 ml) and formaldehyde (36% in water, 125 μl, 1.5 mmol) was added. The mixture was stirred for 3 days at reflux temperature during the days (3 × 8 h) and 45 °C for overnights (3 × 16 h). The solvent was evaporated and the residue was purified by flash column chromatography (hexane/acetone 99:1) to yield **14** (67 mg, 42%) as a yellowish syrup.

*R*_f_ = 0.35 (hexane/acetone 9:1); ^1^H NMR (500 MHz, MeOD): *δ* (ppm) 6.14 (s, 2H, aromatic C*H*), 5.41 (s, 2H, H-2 C*H*), 4.40 (d, 4H, *J* = 11.8 Hz, H-9 C*H*_2_), 3.97 (d, 2H,* J* = 10.2 Hz, H-3 C*H*), 3.85 (s, 2H, methylene C*H*_2_), 2.74–2.59 (m, 2H, H-4 C*H*), 2.49–2.31 (m, 4H, H-1″ C*H*_2_), 2.29–2.15 (m, 2H, H-6 C*H*_2_a), 2.04 (d, 1H, *J* = 17.4 Hz, H-6 C*H*_2_b), 1.79–1.68 (m, 10H, H-5 C*H*_2_ and H-7 C*H*_3_), 1.61 (s, 3H, H-10 C*H*_3_), 1.39–1.18 (m, 12H, H-2″, H-3″ and H-4″ C*H*_2_), 0.86 (t, 6H, *J* = 6.9 Hz, H-5″ C*H*_3_); ^13^C NMR (125 MHz, MeOD): *δ* (ppm) 155.0 (quat.), 126.6 (2C, C-2 *C*H), 111.3 (2C, C-9 CH), 109.7 (2C, Aromatic *C*H from HSQC), 47.0 (2C, C-4 CH), 37.7 (2C, C-3 *C*H), 34.3 (2C, C-1″ *C*H_2_), 33.2, 31.9 (4C, C-2″ and C-3″ *C*H_2_), 31.6 (2C, C-6 *C*H_2_), 30.2 (2C, C-5 *C*H_2_), 23.8, (2C, C-7, *C*H_3_), 23.7 (2C, C-4″ *C*H_2_), 22.9 (1C, Methylene *C*H_2_ from HSQC), 19.6 (2C, C-10, *C*H_3_), 14.5 (2C, C-5″ *C*H_3_); MALDI-TOF MS: *m/z* calcd for C_43_H_60_O_4_Na^+^: 663.4384 [M+H]^+^; found: 663.4385.

#### Compound **16a**

Paraformaldehyde (300 mg, 10 mmol) and *n*-butylamine (**3**) (300 μl, 3 mmol) was dissolved in ethanol (10 ml) then the mixture was stirred for 30 min and cannabigerol (158 mg, 0.5 mmol) was added. The reaction mixture was stirred at reflux temperature for 3 h. The solvent was evaporated and the residue was purified by flash column chromatography (hexane/acetone 95:5) to yield **16a** (25 mg, 10%) as a yellowish syrup.

*R*_f_ = 0.76 (hexane/acetone 8:2); ^1^H NMR (500 MHz, CDCl_3_): *δ* (ppm) 6.27 (s, 1H, aromatic C*H*), 5.31 (bs, 1H, OH), 5.28–5.23 (m, 1H, H-2′ C*H*_2_), 5.09–5.03 (m, 1H, H-6′ C*H*_2_), 4.80 (s, 2H, H-B C*H*_2_), 3.89 (s, 2H, H-A C*H*_2_), 3.34 (d, 2H, *J* = 7.0 Hz, H-1′ C*H*_2_), 2.73–2.67 (m, 2H, Bu-C*H*_2_), 2.39–2.33 (m, 2H, H-1″ C*H*_2_), 2.12–2.05 (m, 2H, H-5′ C*H*_2_), 2.05–2.00 (m, 2H, H-4′ C*H*_2_), 1.79 (s, 3H, H-9′ C*H*_3_), 1.67, 1.58 (2xs, 6H, H-8′ and H-10′ C*H*_3_), 1.57–1.47 (m, 4H, H-2″ and Bu C*H*_2_), 1.40–1.30 (m, 6H, H-3″, H-4″ and Bu C*H*_2_), 0.96–0.86 (m, 6H, H-5″ and Bu C*H*_3_); ^13^C NMR (125 MHz, CDCl_3_): *δ* (ppm) 153.8, 152.2, 139.4, 138.0, 131.9, (5C, quat.), 124.1 (1C, C-6′ *C*H), 122.3 (1C, C-2′ *C*H), 111.8, 110.3 (2C, quat.), 108.8 (1C, aromatic *C*H), 82.2 (1C, C-B *C*H_2_), 51.4 (1C, Bu *C*H_2_) 48.4 (1C, C-A *C*H_2_), 39.9 (1C, C-4′ *C*H_2_), 32.0, 31.9 (2C, C-1″ and C-3″ *C*H_2_), 30.5, 30.0 (2C, C-2″ and Bu *C*H_2_), 26.7 (1C, C-5′ *C*H_2_), 25.8 (1C, C-8′ or C-10′ *C*H_3_), 22.7, 22.1, 20.6 (3C, C-1′, C-4″ and Bu *C*H_2_), 17.8 (1C, C-8′ or C-10′ *C*H_3_), 16.3 (1C, C-9′ *C*H_3_), 14.2 (2C, C-5″ and Bu *C*H_3_); ESI-QqTOF MS: *m/z* calcd for C_27_H_42_NO_2_^−^: 412.322 [M-H]^−^; found: 412.322.

#### Compound **16b**

***Reaction route F2:*** Formaldehyde (36% in water, 209 μl, 2.5 mmol) and *n*-butylamine (**3**) (247 μl, 2.5 mmol) was dissolved in ethanol (10 ml), and the mixture was stirred for 30 min, then cannabigerol (158 mg, 0.5 mmol) was added. The reaction mixture was stirred at reflux temperature for 20 h, then the solvent was evaporated and the residue was purified by flash column chromatography (hexane/acetone 99:1) to yield **16b** (173 mg, 67%, impure, contains a by-product equipped with ethoxymethyl side chain according to NMR spectra) as a yellowish syrup.

***Reaction route F3:*** Reaction was performed on the basis of the ***reaction route F2***. The solvent was 1,4-dioxane (10 ml) The reaction mixture was stirred for 48 h at reflux temperature during the days (2 × 8 h) and 70 °C for overnights (2 × 16 h). Column chromatography (hexane/acetone 99:1) yielded **16b** (134 mg, 52%) as a yellowish syrup.

*R*_f_ = 0.76 (hexane/acetone 8:2); ^1^H NMR (500 MHz, CDCl_3_): *δ* (ppm) 5.25–5.19 (m, 1H, H-2′ C*H*), 5.11–5.05 (m, 1H, H-6′ C*H*), 4.79 (s, 4H, H-B C*H*_2_), 3.90 (s, 4H, H-A C*H*_2_), 3.24 (d, 2H, *J* = 7.1 Hz, H-1′ C*H*_2_), 2.73–2.67 (m, 4H, butyl C*H*_2_), 2.30–2.24 (m, 2H, H-1″ C*H*_2_), 2.07–2.01 (m, 2H, H-5′ C*H*_2_), 1.96–1.91 (m, 2H, H-4′ C*H*_2_), 1.74 (s, 3H, H-9′ C*H*_3_), 1.64 (s, 3H, H-8′ or H-10′ C*H*_3_), 1.57 (s, 3H, H-8′ or H-10′ C*H*_3_), 1.56–1.50 (m, 4H, butyl C*H*_2_), 1.41–1.31 (m, 10H, 2xbutyl C*H*_2_, H-2″, H-3″ and H-4″ C*H*_2_), 0.95–0.88 (m, 9H, H-5″ and 2xbutyl C*H*_3_); ^13^C NMR (125 MHz, CDCl_3_): *δ* (ppm) 151.1, 135.5, 134.5, 131.1 (5C, quat.), 124.8 (1C, C-6′ *C*H), 123.0 (1C, C-2′ *C*H), 114.1, 110.4 (3C, quat.), 81.8 (2C, C-B *C*H_2_), 51.3 (2C, butyl *C*H_2_), 48.5 (2C, C-A *C*H_2_), 40.0 (1C, C-4′ *C*H_2_), 32.4 (1C, *C*H_2_), 30.5 (2C, butyl *C*H_2_), 29.3 (1C, *C*H_2_), 27.6 (1C, C-1″ *C*H_2_), 26.9 (1C, C-5′ *C*H_2_), 25.8 (1C, *C*H_3_), 22.6 (1C, *C*H_2_), 21.6 (1C, C-1′ *C*H_2_), 20.6 (2C, butyl *C*H_2_), 17.8 (1C, *C*H_3_), 16.2 (1C, C-9′ *C*H_3_), 14.1 (3C, C-5″ and 2xbutyl *C*H_3_); UHR ESI-QTOF MS: *m/z* calcd for C_33_H_55_N_2_O_2_^+^: 511.4258 [M+H]^+^; found: 511.4259.

#### Compounds **17a** and **17b**

Formaldehyde (36% in water, 417 μl, 5 mmol) and benzylamine (**4**) (1.83 ml, 5 mmol) was dissolved in dioxane (10 ml) and it was stirred for 30 min, then cannabigerol (316 mg, 1 mmol) was added. The mixture was stirred at reflux temperature for 8 h and 70 °C for 18 h. Then the solvent was evaporated, the residue was dissolved in dichloromethane (200 ml) and it was washed with water 3 times (3 × 10 ml). The organic phase was dried over Na_2_SO_4_, then filtered and the solvent was evaporated in vacuum. The residue was purified by flash column chromatography (hexane/acetone 98:2) to yield **17a** (370 mg, 64%) and **17b** (161 mg, 35%) as yellowish syrups.

**17a**: *R*_f_ = 0.46 (hexane/acetone 8:2); ^1^H NMR (500 MHz, CDCl_3_): *δ* (ppm) 7.41–7.23 (m, 5H, benzyl C*H*), 6.27 (s, 1H, aromatic C*H*), 5.45 (bs, 1H, OH), 5.33–5.26 (m, 1H, H-2′ C*H*), 5.11–5.04 (m, 1H, H-6′ C*H*), 4.83 (s, 2H, H-B C*H*_2_), 3.88 (m, 4H, H-A and benzyl C*H*_2_), 3.38 (d, 2H, *J* = 7.1 Hz, H-1′ C*H*_2_), 2.32–2.23 (m, 2H, H-1″ C*H*_2_), 2.14–2.07 (m, 2H, H-5′ C*H*_2_), 2.07–2.01 (m, 4H, H-4′ C*H*_2_), 1.80 (s, 3H, H-9′ C*H*_3_), 1.67 (s, 3H, H-8′ or H-10′ C*H*_3_), 1.59 (s, 3H, H-8′ or H-10′ C*H*_3_), 1.49–1.40 (m, 2H, H-2″ C*H*_2_), 1.32–1.21 (m, 4H, H-3″ and H-4″ C*H*_2_), 0.89–0.82 (m, 3H, H-5″ C*H*_3_); ^13^C NMR (125 MHz, CDCl_3_): *δ* (ppm) 153.8, 152.2, 139.5, 138.4, 137.7, 131.8 (6C, quat.), 129.2, 128.5, 127.4 (5C, benzyl *C*H), 124.2 (1C, C-6′ *C*H), 122.4 (1C, C-2′ *C*H), 112.0, 109.9 (2C, quat.) 109.0 (1C, aromatic *C*H), 81.9 (1C, C-B *C*H_2_), 55.8 and 47.8 (2C, C-A and benzyl *C*H_2_), 39.9 (1C, C-4′ *C*H_2_), 31.87 and 31.85 (2C, C-1″ and C-3″*C*H_2_), 29.9 (1C, C-2″ *C*H_2_), 26.7 (1C, C-5′ *C*H_2_), 25.7 (1C, *C*H_3_), 22.7 (1C, C-4″ *C*H_2_), 22.1 (1C, C-1′ *C*H_2_), 17.8 (1C, *C*H_3_), 16.3 (1C, C-9′ *C*H_3_), 14.1 (1C, C-5″ *C*H_3_); UHR ESI-QTOF MS: *m/z* calcd for C_30_H_42_NO_2_^+^: 448.3210 [M+H]^+^; found: 448.3209.

**17b**: *R*_f_ = 0.69 (hexane/acetone 8:2); ^1^H NMR (400 MHz, CDCl_3_): *δ* (ppm) 7.40–7.21 (m, 10H, benzyl C*H*), 5.37–5.29 (m, 1H, H-2′ C*H*), 5.15–5.08 (m, 1H, H-6′ C*H*), 4.82 (s, 4H, H-B C*H*_2_), 3.88 (s, 8H, H-A and benzyl C*H*_2_), 3.33 (d, 2H, *J* = 7.1 Hz, H-1′ C*H*_2_), 2.16–2.06 (m, 4H, H-1″ and H-5′ C*H*_2_), 2.05–1.98 (m, 2H, H-4′ C*H*_2_), 1.79 (s, 3H, H-9′ C*H*_3_), 1.65 (s, 3H, H-8′ or H-10′ C*H*_3_), 1.58 (s, 3H, H-8′ or H-10′ C*H*_3_), 1.29–1.13 (m, 6H, H-2″, H-3″ and H-4″ C*H*_2_), 0.83–0.76 (m, 3H, H-5″ C*H*_3_); ^13^C NMR (100 MHz, CDCl_3_): *δ* (ppm) 151.0, 138.5, 136.0, 134.6, 131.1 (7C, quat.), 129.2, 128.4, 127.4 (10C, aromatic *C*H), 124.7 (1C, C-2′ *C*H), 123.0 (1C, C-6′ *C*H) 114.1, 110.2 (3C, quat.), 81.5 (2C, C-B *C*H_2_), 55.7, 47.9 (4C, C-A and benzyl *C*H_2_), 40.0 (1C, C-4′ *C*H_2_), 32.2 (1C, C-3″ *C*H_2_), 29.2 (1C, C-2″ *C*H_2_), 27.5, 27.0 (2C, C-1″ and C-5′ *C*H_2_), 25.8 (1C, *C*H_3_), 22.5 (1C, C-4″ *C*H_2_), 21.7 (1C, C-1′ *C*H_2_), 17.8 (1C, *C*H_3_), 16.2 (1C, C-9′ *C*H_3_), 14.0 (1C, C-5″ *C*H_3_); ESI-QqTOF MS: *m/z* calcd for C_39_H_50_N_2_O_2_Na^+^: 601.376; found: 601.375.

#### Compound **18**

Formaldehyde (36% in water 167 μl, 2 mmol) and propargylamine (**5**) (128 μl, 2 mmol) was dissolved in dioxane (10 ml). The mixture was stirred for 30 min then cannabigerol (316 mg, 1 mmol) was added. The mixture was stirred at room temperature for 48 h and then 70 °C for additional 48 h. Then the solvent was evaporated, the residue was dissolved in dichloromethane (200 ml) and it was washed with water 3 times (3 × 10 ml). The organic phase was dried over Na_2_SO_4_, then filtered and the solvent was evaporated in vacuum. The residue was purified by flash column chromatography (hexane/acetone 98:2) to yield **18** (74 mg, 19%) as a yellowish syrup.

*R*_f_ = 0.56 (hexane/acetone 8:2); ^1^H NMR (400 MHz, CDCl_3_): *δ* (ppm) 6.30 (s, 1H, aromatic C*H*), 5.50 (bs, 1H, OH), 5.23 (t, 1H, *J* = 7.2 Hz, H-2′ C*H*), 5.05 (t, 1H, *J* = 6.8 Hz, H-6′ C*H*), 4.86 (s, 2H, H-B C*H*_2_), 4.01 (s, 2H, H-A C*H*_2_), 3.56 (d, 2H, *J* = 2.5 Hz, propargyl C*H*_2_), 3.34 (d, 2H, *J* = 7.1 Hz, H-1′ C*H*_2_), 2.40–2.33 (m, 2H, H-1″ C*H*_2_), 2.31–2.27 (m, 1H, propargyl C*H*), 1.99–2.13 (m, 4H, H-4′ and H-5′ C*H*_2_), 1.78 (s, 3H, H-9′ C*H*_3_), 1.67 (s, 3H, H-8′ or H-10′ C*H*_3_), 1.58 (s, 3H, H-8′ or H-10′ C*H*_3_), 1.56–1.46 (m, 2H, H-2″ C*H*_2_), 1.39–1.29 (m, 4H, H-3″ and H-4″ C*H*_2_), 0.85–0.93 (m, 3H, H-5″ C*H*_3_); ^13^C NMR (100 MHz, CDCl_3_): *δ* (ppm) 153.9, 151.6, 139.5, 137.9, 131.9 (6C, quat.), 124.1 (1C, C-6′ *C*H), 122.2 (1C, C-2′ *C*H), 112.1 (2C, quat.), 109.3 (1C, aromatic *C*H), 81.1 (1C, C-B *C*H_2_), 80.0 (1C, propargyl quat.), 72.9 (1C, propargyl *C*H) 47.8 (1C, C-A *C*H_2_), 41.1 (1C, propargyl *C*H_2_), 39.8 (1C, C-4′ *C*H_2_), 31.89 and 31.86 (2C, C-1″ and C-3″ *C*H_2_), 29.9 (1C, C-2″ *C*H_2_), 26.6 (1C, C-5′ *C*H_2_), 25.8 (1C, *C*H_3_), 22.7 (1C, C-4″ *C*H_2_), 22.0 (1C, C-1′ *C*H_2_), 17.8 (1C, *C*H_3_), 16.2 (1C, C-9′ *C*H_3_), 14.1 (1C, C-5″ *C*H_3_); UHR ESI-QTOF MS: *m/z* calcd for C_26_H_38_NO_2_^+^: 396.2897 [M+H]^+^; found: 396.2897.

#### Compounds **19a** and **19b**

Formaldehyde (36% in water 167 μl, 2 mmol) and allyl amine (**15**) (150 μl, 2 mmol) was dissolved in dioxane (10 ml). The mixture was stirred for 30 min and after cannabigerol (316 mg, 1 mmol) was added. The mixture was stirred at 45 °C for 6 days, then the solvent was evaporated, the residue was dissolved in dichloromethane (200 ml) and it was washed with water 3 times (3 × 10 ml). The organic phase was dried over Na_2_SO_4_, then filtered and the solvent was evaporated in vacuum. The residue was purified by flash column chromatography (hexane/acetone 98:2) to yield **19a** (262 mg, 66%) and **19b** (132 mg, 27%) as yellowish syrups, respectively.

**19a**: *R*_f_ = 0.31 (hexane/acetone 8:2); ^1^H NMR (400 MHz, CDCl_3_): *δ* (ppm) 6.26 (s, 1H, aromatic C*H*), 5.99–5.85 (m, 1H, allyl C*H*), 5.29–5.14 (m, 3H, H-2′ C*H* and allyl C*H*_2_), 5.10–5.02 (m, 1H, H-6′ C*H*), 4.82 (s, 2H, H-B C*H*_2_), 3.89 (s, 2H, H-A C*H*_2_), 3.38–3.31 (m, 4H, H-1′ and allyl C*H*_2_), 2.38–2.29 (m, 2H, H-1″ C*H*_2_), 2.14–2.05 (m, 2H, H-5′ C*H*_2_), 2.05–1.98 (m, 2H, H-4′ C*H*_2_), 1.78 (s, 3H, H-9′ C*H*_3_), 1.66 (s, 3H, H-8′ or H-10′ C*H*_3_), 1.58 (s, 3H, H-8′ or H-10′ C*H*_3_), 1.54–1.44 (m, 2H, H-2″ C*H*_2_), 1.37–1.29 (m, 4H, H-3″ and H-4″ C*H*_2_), 0.93–0.85 (m, 3H, H-5″ C*H*_3_); ^13^C NMR (100 MHz, CDCl_3_): *δ* (ppm) 153.7, 152.1, 139.4, 137.5 (4C, quat.), 135.3 (1C, allyl *C*H), 131.8 (1C, quat.), 124.1 (1C, C-6′ *C*H), 122.3 (1C, C-2′ *C*H), 118.4 (1C, allyl *C*H_2_), 112.1, 109.7 (2C, quat.), 108.9 (1C, aromatic *C*H), 81.7 (1C, C-B *C*H_2_), 54.8 (1C, allyl *C*H_2_), 47.5 (1C, C-A *C*H_2_), 39.9 (1C, C-4′ *C*H_2_), 31.9 (2C, C-1″ and C-3″ *C*H_2_), 30.0 (1C, C-2″ *C*H_2_), 26.6 (1C, C-5′ *C*H_2_), 25.8 (1C, *C*H_3_), 22.7 (1C, C-4″ *C*H_2_), 22.0 (1C, C-1′ *C*H_2_), 17.8 (1C, *C*H_3_), 16.2 (1C, C-9′ *C*H_3_), 14.1 (1C, C-5″ *C*H_3_; ESI-QqTOF MS: *m/z* calcd for C_26_H_39_NO_2_Na^+^: 420.287 [M+Na]^+^; found: 420.286.

**19b**: *R*_f_ = 0.48 (hexane/acetone 8:2); ^1^H NMR (400 MHz, CDCl_3_): *δ* (ppm) 6.01–5.89 (m, 2H, allyl C*H*), 5.29–5.18 (m, 5H, H-2′ C*H* and 2xallyl C*H*_2_), 5.08–5.15 (m, 1H, H-6′ C*H*), 4.85 (s, 4H, H-B C*H*_2_), 3.94 (s, 4H, H-A C*H*_2_), 3.39 (dd, 4H, *J* = 6.5, 1.5 Hz, allyl C*H*_2_), 3.28 (d, 2H, *J* = 7.1 Hz H-1′ C*H*_2_), 2.30 (m, 2H, H-1″ C*H*_2_), 2.13–2.04 (m, 2H, H-5′ C*H*_2_), 2.02–1.95 (m, 2H, H-4′ C*H*_2_), 1.78 (s, 3H, H-9′ C*H*_3_), 1.68 (s, 3H, H-8′ or H-10′ C*H*_3_), 1.61 (s, 3H, H-8′ or H-10′ C*H*_3_), 1.43–1.31 (m, 6H, H-2″,H-3″ and H-4″ C*H*_2_), 0.97–0.89 (m, 3H, H-5″ C*H*_3_); ^13^C NMR (100 MHz, CDCl_3_): *δ* (ppm) 150.9, 135.7 (3C, quat.), 135.5 (2C, allyl *C*H), 134.5, 131.0 (2C, quat.), 124.6 (1C, C-6′ *C*H), 122.8 (1C, C-2′ *C*H), 118.1 (2C, allyl *C*H_2_), 114.0, 110.1 (3C, quat.), 81.5 (2C, C-B *C*H_2_), 54.7 (2C, allyl *C*H_2_), 47.5 (2C, C-A *C*H_2_), 39.9 (1C, C-4′ *C*H_2_), 32.3, 29.2, 27.5, 26.8 (4C, C-3″, C-1″, C-2′ and C5′ *C*H_2_), 25.7 (1C, *C*H_3_), 22.5, 21.5 (2C, C-4″ and C-1′ *C*H_2_), 17.7 (1C, *C*H_3_), 16.1 (1C, C-9′ *C*H_3_), 14.0 (1C, C-5″ *C*H_3_); ESI-QqTOF MS: *m/z* calcd for C_31_H_46_N_2_O_2_Na^+^: 501.345 [M+Na]^+^; found: 501.345.

#### Compound **20**

Formaldehyde (36% in water, 167 μl, 2 mmol) and γ-aminobutyric acid (**6**) (206 mg, 2 mmol) was dissolved in the mixture of dioxane (10 ml) and water (1 ml). It was stirred for 30 min, then cannabigerol (316 mg, 1 mmol) was added. The mixture was stirred at room temperature for 7 days. The solvent was evaporated and the product was purified by flash column chromatography (hexane/acetone 95:5) to yield **20** (317 mg, 71%) as a colourless syrup.

*R*_f_ = 0.58 (hexane/acetone 8:2); ^1^H NMR (400 MHz, CDCl_3_): *δ* (ppm) 8.02 (bs, 2H, OH, COOH), 6.30 (s, 1H, aromatic C*H*) 5.30–5.19 (m, 1H, H-2′ C*H*), 5.11–5.00 (m, 1H, H-6′ C*H*), 4.79 (s, 2H, H-B C*H*_2_), 3.90 (s, 2H, H-B C*H*_2_), 3.33 (d, 2H, *J* = 7.1 Hz, H-1′ C*H*_2_), 2.80 (t, 2H, *J* = 6.8 Hz, GABA C*H*_2_), 2.46 (t, 2H, *J* = 7.0 Hz, H-1″ C*H*_2_), 2.39–2.30 (m, 2H, GABA C*H*_2_), 2.13–1.97 (4H, H-4′ and H-5′ C*H*_2_), 1.89 (q, 2H, *J* = 6.9 Hz, GABA C*H*_2_), 1.77 (s, 3H, H-9′ C*H*_3_), 1.66 (s, 3H, H-8′ or H-10′ C*H*_3_), 1.58 (s, 3H, H-8′ or H-10′ C*H*_3_), 1.54–1.44 (m, 2H, H-2″ C*H*_2_), 1.40–1.28 (m, 4H, H-3″ and H-4″ C*H*_2_), 0.95–0.82 (m, 3H, H-5″ C*H*_3_); ^13^C NMR (100 MHz, CDCl_3_): *δ* (ppm) 177.9 (1C, *C* = O), 153.9, 151.8, 139.4, 137.6, 131.8 (5C, quat.), 124.1 (1C, C-6′ *C*H), 122.2 (1C, C-2′ *C*H), 112.2, 109.2 (2C, quat.), 109.2 (1C, C-4, aromatic *C*H), 81.5 (1C, C-B *C*H_2_), 51.0 (1C, GABA *C*H_2_), 48.0 (1C, C-A *C*H_2_), 39.8 (1C, C-4′ *C*H_2_), 32.7 (1C, C-1″ *C*H_2_), 31.9, 31.8 (2C, C-3″ and GABA *C*H_2_), 29.8 (1C, C-2″ *C*H_2_), 26.6 (1C, C-5′ *C*H_2_), 25.8 (1C, *C*H_3_), 22.8 (1C, GABA *C*H_2_), 22.7 (1C, C-4″ *C*H_2_), 22.0 (1C, C-1′ *C*H_2_), 17.8 (1C, *C*H_3_), 16.2 (1C, C-9′ *C*H_3_), 14.1 (1C, C-5″ *C*H_3_); UHR ESI-QTOF MS: *m/z* calcd for C_27_H_41_NO_4_Na^+^: 466.2928 [M+Na]^+^; found: 466.2925.

#### Compound **21a**

Formaldehyde (36% in water, 125 μl, 1.5 mmol) and diethylamine (**7**) (155 μl, 1.5 mmol) was dissolved in abs. ethanol (5 ml) and it was stirred for 20 min, then cannabigerol (158 mg, 0.5 mmol) was added. The mixture was stirred at reflux temperature for 6 h and then 70 °C for 18 h. The solvent was evaporated and the residue was purified by flash column chromatography (hexane/ethyl acetate 9:1) to yield **21a** (134 mg, 67%) as a yellowish syrup.

*R*_f_ = 0.46 (hexane/acetone 8:2); ^1^H NMR (400 MHz, CDCl_3_): *δ* (ppm) 8.64 (bs, 2H, OH), 6.15 (s, 1H, aromatic C*H*), 5.37–5.24 (m, 1H, H-2′ C*H*), 5.12–5.02 (m, 1H, H-6′ C*H*), 3.70 (s, 2H, methylene C*H*_2_), 3.40 (d, 2H, *J* = 7.2 Hz, H-1′ C*H*_2_), 2.60 (q, 4H, *J* = 7.2 Hz, ethyl C*H*_2_), 2.43–2.39 (m, 2H, H-1″ C*H*_2_), 2.15–1.98 (m, 4H, H-4′ and H-5′ C*H*_2_), 1.80 (s, 3H, H-9′ C*H*_3_), 1.66 (s, 3H, H-8′ or H-10′ C*H*_3_), 1.58 (s, 3H, H-8′ or H-10′ C*H*_3_), 1.53–1.41 (m, 2H, H-2″ C*H*_2_), 1.41–1.28 (m, 2H, H-3″ and H-4″ C*H*_2_), 1.10 (t, 6H, *J* = 7.2 Hz, ethyl C*H*_3_), 0.94–0.83 (m, 3H, H-5″ C*H*_3_); ^13^C NMR (100 MHz, CDCl_3_): *δ* (ppm) 157.4, 154.7, 139.6, 137.7, 131.8 (5C, quat.), 124.2 (1C, C-6′ *C*H), 122.7 (1C, C-2′ *C*H), 111.9, 111.6 (2C, quat.), 107.6 (1C, aromatic *C*H), 52.3 (1C, methylene *C*H_2_), 46.2 (2C, ethyl *C*H_2_), 39.9 (1C, C-4′ *C*H_2_), 33.5 (1C, C-1″ *C*H_2_), 31.9 (1C, C-3″ *C*H_2_), 30.9 (1C, C-2″ *C*H_2_), 26.6 (1C, C-5′ *C*H_2_), 25.8 (1C, *C*H_3_), 22.7 (1C, C-4″ *C*H_2_), 22.3 (1C, C-1′ *C*H_2_), 17.8 (1C, *C*H_3_), 16.3 (1C, C-9′ *C*H_3_), 14.1 (1C, C-5″ *C*H_3_), 11.4 (2C, ethyl *C*H_3_); ESI-QqTOF MS: *m/z* calcd for C_26_H_44_NO_2_^+^: 402.336 [M+H]^+^; found: 402.333.

#### Compounds **21b**

Formaldehyde (36% in water 790 μl, 9.48 mmol) and diethylamine (**7**) (980 μl, 9.48 mmol) was dissolved in dioxane (20 ml) and it was stirred for 20 min then cannabigerol (1 g, 3.16 mmol) was added. The mixture was stirred for 48 h at reflux temperature during the days (2 × 8 h) and 70 °C at night (2 × 16 h). The solvent was evaporated and the residue was purified by flash column chromatography (hexane/ethyl acetate 99:1) to yield **21b** (939 mg, 61%) as a yellowish syrup.

*R*_f_ = 0.40 (hexane/acetone 8:2); ^1^H NMR (400 MHz, CDCl_3_): *δ* (ppm) 5.33 (t, 1H, *J* = 6.7 Hz, H-2′ C*H*), 5.13–5.05 (m, 1H, H-6′ C*H*), 3.71 (s, 4H, methylene C*H*_2_), 3.34 (d, 2H, *J* = 6.7 Hz, H-1′ C*H*_2_), 2.59 (q, 8H, *J* = 7.1 Hz, ethyl C*H*_2_), 2.48–2.38 (m, 2H, H-1″ C*H*_2_), 2.12–2.01 (m, 2H, H-5′ C*H*_2_), 2.01–1.92 (m, 2H, C-4′ C*H*_2_), 1.79 (s, 3H, H-9′ C*H*_3_), 1.64 (s, 3H, H-8′ or H-10′ C*H*_3_), 1.57 (s, 3H, H-8′ or H-10′ C*H*_3_), 1.42–1.30 (m, 6H, H-2″, H-3″ and H-4″ C*H*_2_), 1.10 (t, 12H, *J* = 7.2 Hz, ethyl C*H*_3_), 0.96–0.87 (m, 3H, H-5″ C*H*_3_); ^13^C NMR (100 MHz, CDCl_3_): *δ* (ppm) 156.9, 136.4, 134.3, 130.9 (5C, quat.), 125.0 (1C, C-6′ *C*H), 123.8 (1C, C-2′ *C*H), 114.1, 110.1 (3C, quat.), 53.0 (2C, methylene *C*H_2_), 46.2 (4C, ethyl *C*H_2_), 40.0 (1C, C-4′ *C*H_2_), 32.2, 30.6, 29.0 and 27.0 (4C, C-1″, C-3″, C-2″ and C-5′ *C*H_2_), 25.8 (1C, *C*H_3_), 22.6 (1C, C-4″ *C*H_2_), 22.3 (1C, C-1′ *C*H_2_), 17.7 (1C, *C*H_3_), 16.4 (1C, C-9′ *C*H_3_), 14.2 (1C, C-5″ *C*H_3_), 11.4 (4C, ethyl *C*H_3_); UHR ESI-QTOF MS: *m/z* calcd for C_31_H_55_N_2_O_2_^+^: 487.4258 [M+H]^+^; found: 487,4260.

### Supplementary Information

Supplementary Information.

## Data Availability

The datasets used and/or analyzed during the current study available in the here and in the Supplementary Information file or from the corresponding author (Ilona Bereczki) on reasonable request.
