# Supplementary material for: Mannich-type modifications of (−)-cannabidiol and (−)-cannabigerol leading to new, bioactive derivatives
Source: Sci Rep. 2023 Nov 10;13:19618. doi: 10.1038/s41598-023-45565-7 (PMC10638401; doi:10.1038/s41598-023-45565-7)

Supplementary information

Mannich-Type Modifications Of (-)-Cannabidiol And (-)-Cannabigerol Leading To New, Bioactive Derivatives

Eszter Boglárka Lőrincz, Gergely Tóth, Júlia Spolárics, Mihály Herczeg, Jan Hodek, István Zupko, Renáta Minorics, Dorottya Ádám, Attila Oláh, Christos C. Zouboulis, Jan Weber, Lajos Nagy, Eszter Ostorházi, Ildikó Bácskay, Anikó Borbás, Pál Herczegh, Ilona Bereczki

Table of content

1. **Antiproliferative effect of the salt derivative compound 8s** S2
2. **Antibacterial data against Gram-positive bacteria** S3
3. **NMR data of CBD and its derivatives** S4
4. **NMR data of CBG and its derivatives** S22

1. Antiproliferative effect of the salt derivative compound 8s

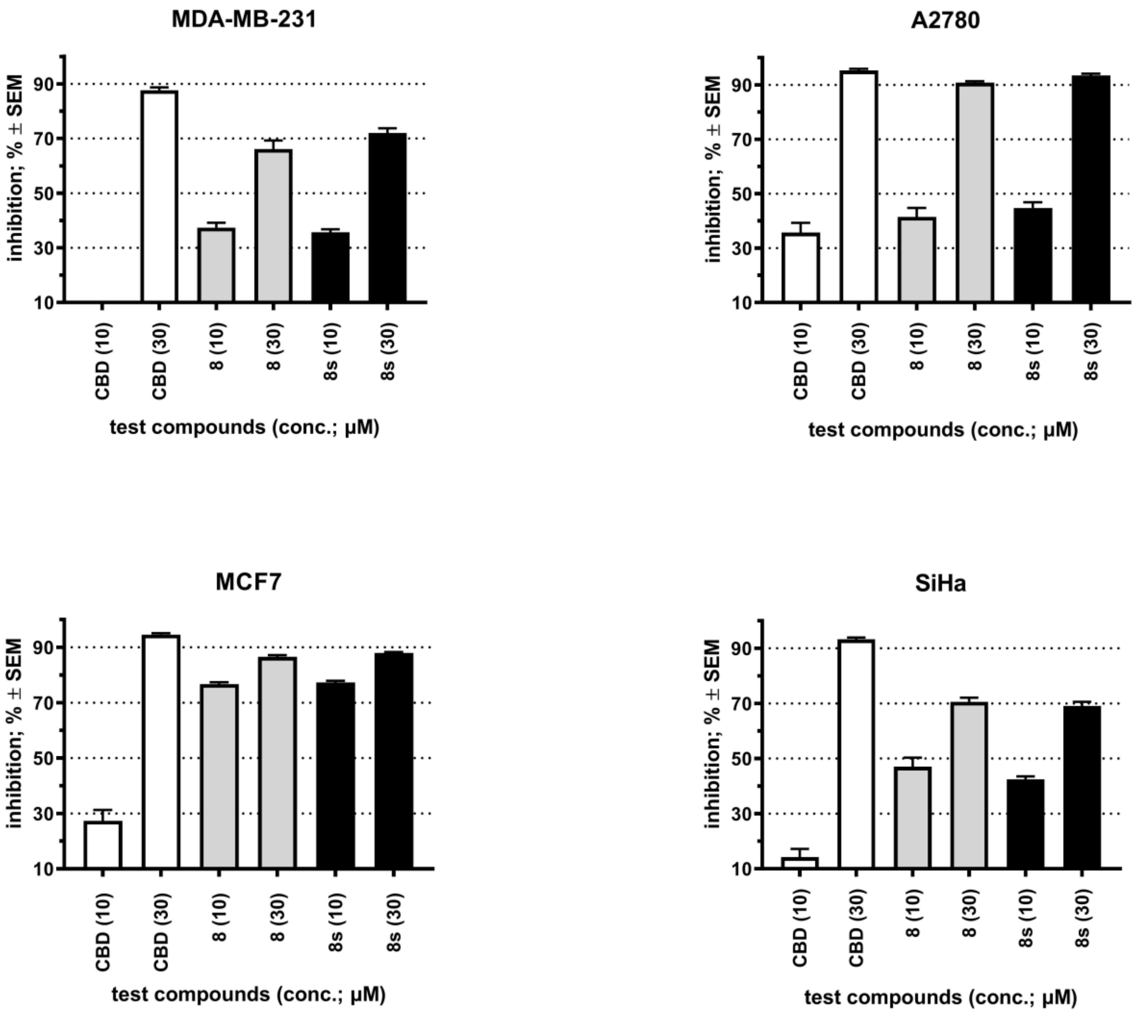

Figure S1. Antiproliferative properties of compound **8s** compared to CBD and its free base derivative (**8**) (10 and 30 µM). Inhibition values less than 10% are considered negligible and not presented for clarity.

2. Antibacterial data against Gram-positive bacteria

Table S1. Antibacterial effects of CBD, CBG and their derivatives against Gram-positive bacteria

	**CBD**	**8**	**8s**	**9**	**10/12/13**	**11**	**CBG**	**16b/17a/ 18/21b**	**20**
	(MIC, µg/ml)								
Bacillus subtilis	4	>256	128	>256	>256	>256	2	>256	>256
**MSSA**	4	>256	128	>256	>256	>256	4	>256	>256
**MRSA**	4	>256	128	>256	>256	>256	4	>256	>256
***Staphylococcus epidermidis* biofilm forming**	8	>256	128	>256	>256	>256	8	>256	>256
***Staphylococcus epidermidis* mecA**	8	>256	128	>256	>256	>256	8	>256	>256
***Enterococcus faecalis* 29 212**	2	2	2	8	>256	128	4	64	64
***Enterococcus faecalis* 15 376 VanA**	4	>256	128	128	>256	>256	4	>256	128
***Enterococcus faecalis* 51299 VanB**	4	2	2	8	>256	128	4	64	128

MIC: Minimum Inhibitory Concentration; MSSA: Methicillin Sensitive *Staphylococcus aureus;* MRSA: Methicillin Resistant *Staphylococcus aureus*; mecA: mecA gene expression; vanA: vanA gene positive; vanB: vanB gene positive.

3. NMR data of CBD and its derivatives

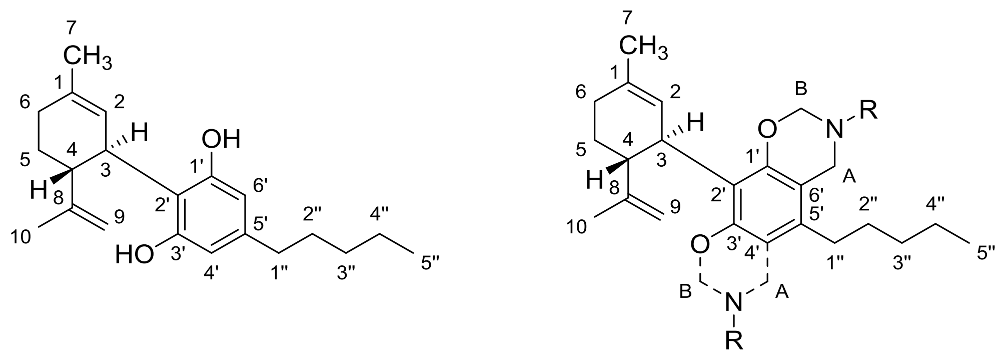

Figure S2. Numbering of CBD and its derivatives.

NMD data of cannabidiol:

^{1}H NMR (500 MHz, $CDCl_3$): δ (ppm) 6.34-6.09 (m, 2H, aromatic C*H*), 6.00 (bs, 1H, O*H*), 5.56 (m, 1H, H-2 C*H*), 4.95 (bs, 1H, O*H*), 4.64 (m, 1H, H-9 C*H*2a), 4.54 (m, 1H, H-9 C*H*2b), 3.90-3.83 (m, 1H, H-3 C*H*), 2.46-2.36 (m, 3H, H-1” C*H*2 and H-4 C*H*), 2.29-2.17 (m, 1H, H-6 C*H*2a), 2.13-2.04 (m, 1H, H-6 C*H*2b), 1.86-1.72 (m, 5H, H-5 C*H*2 and H-7 C*H*3), 1.66 (s, 3H, H-10 C*H*3), 1.59-1.50 (m, 2H, H-2” C*H*2), 1.35-1.22 (m, 4H, H-3” and H-4” C*H*2), 0.87 (t, 3H, *J* = 6.9 Hz, H-5” C*H*3); ^{13}C NMR (125 MHz, $CDCl_3$): δ (ppm) 149.3 (1C, C-8, quat.), 143.1 (1C, C-3’, quat.), 140.1 (1C, C-1, quat.), 124.3 (1C, C-2, *C*H), 113.9 (1C, C-6’, quat.), 111.0 (1C, C-9, *C*H2), 46.3 (1C, C-4, *C*H), 37.2 (1C, C-3, *C*H), 35.6 (1C, C-1”, *C*H2), 31.6 (1C, C-3”, *C*H2), 30.7 (1C, C-2”, *C*H2), 30.5 (1C, C-6, *C*H2), 28.5 (1C, C-5, *C*H2), 23.8 (1C, C-7, *C*H3), 22.6 (1C, C-4”, *C*H2), 20.5 (1C, C-10, *C*H3), 14.1 (1C, C-5”, *C*H3).

NMR spectra of CBD

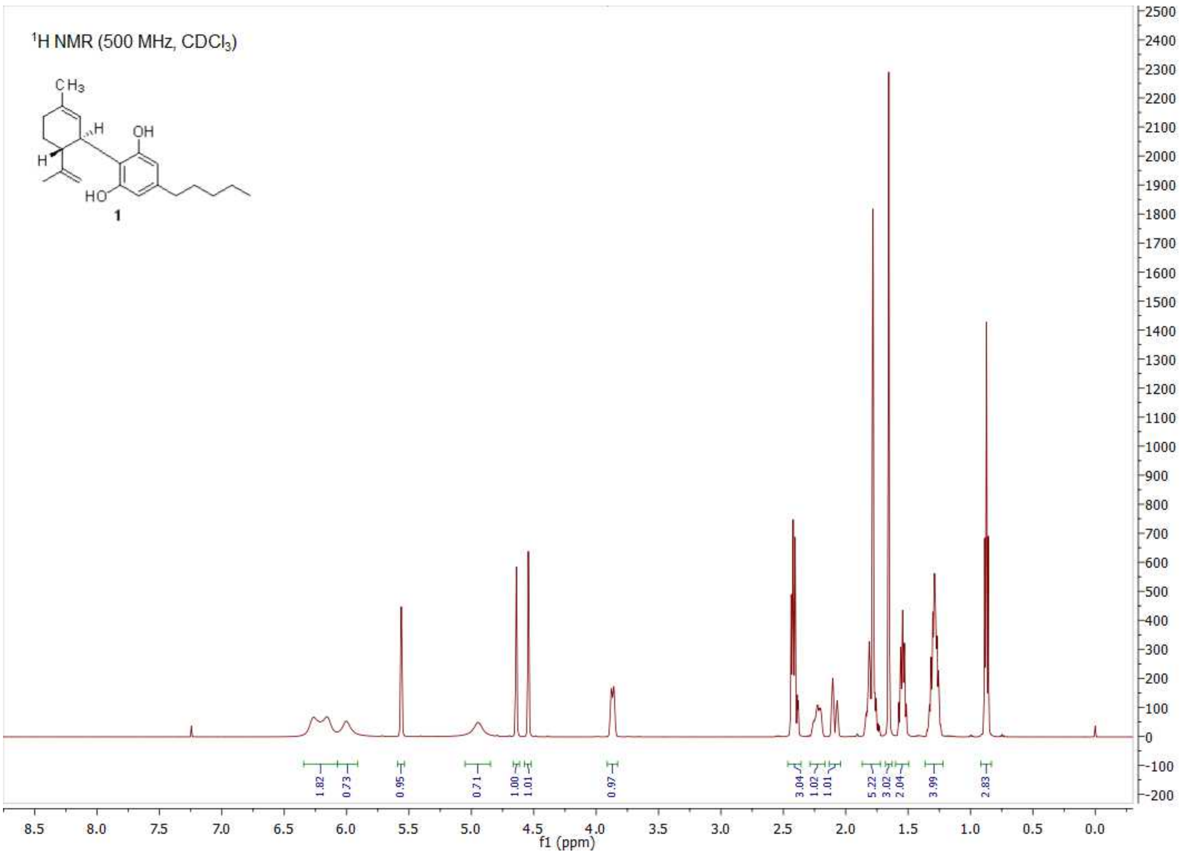

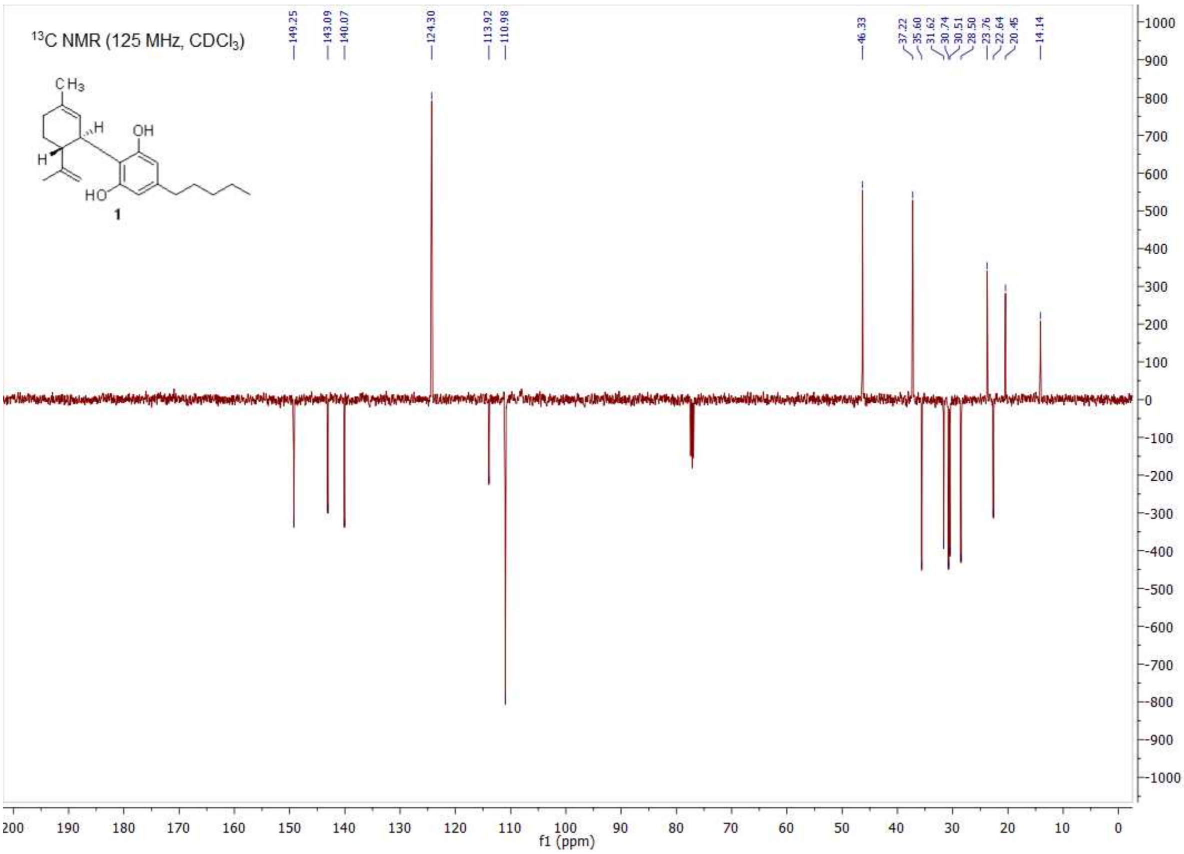

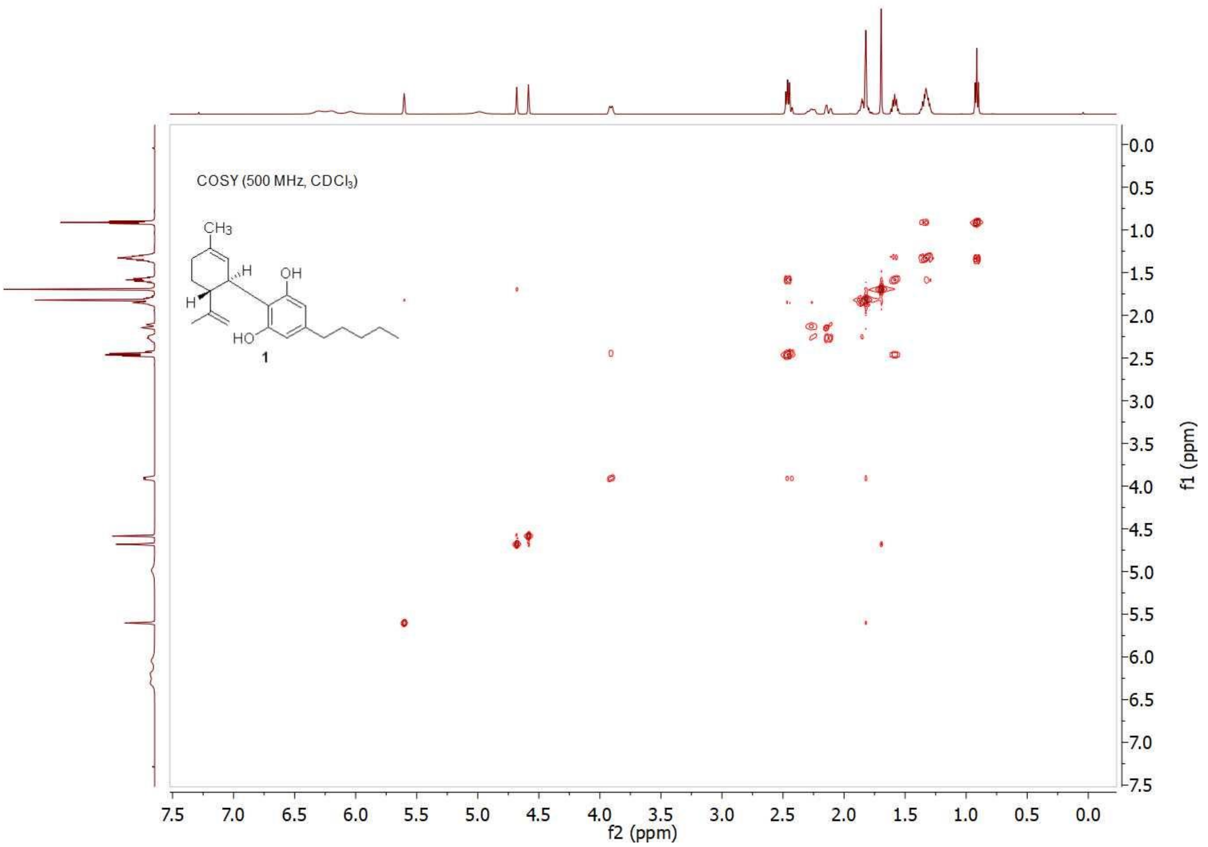
COSY (500 MHz, CDCl3)
CH3
H
OH
H
HO
1
f2 (ppm)
f1 (ppm)

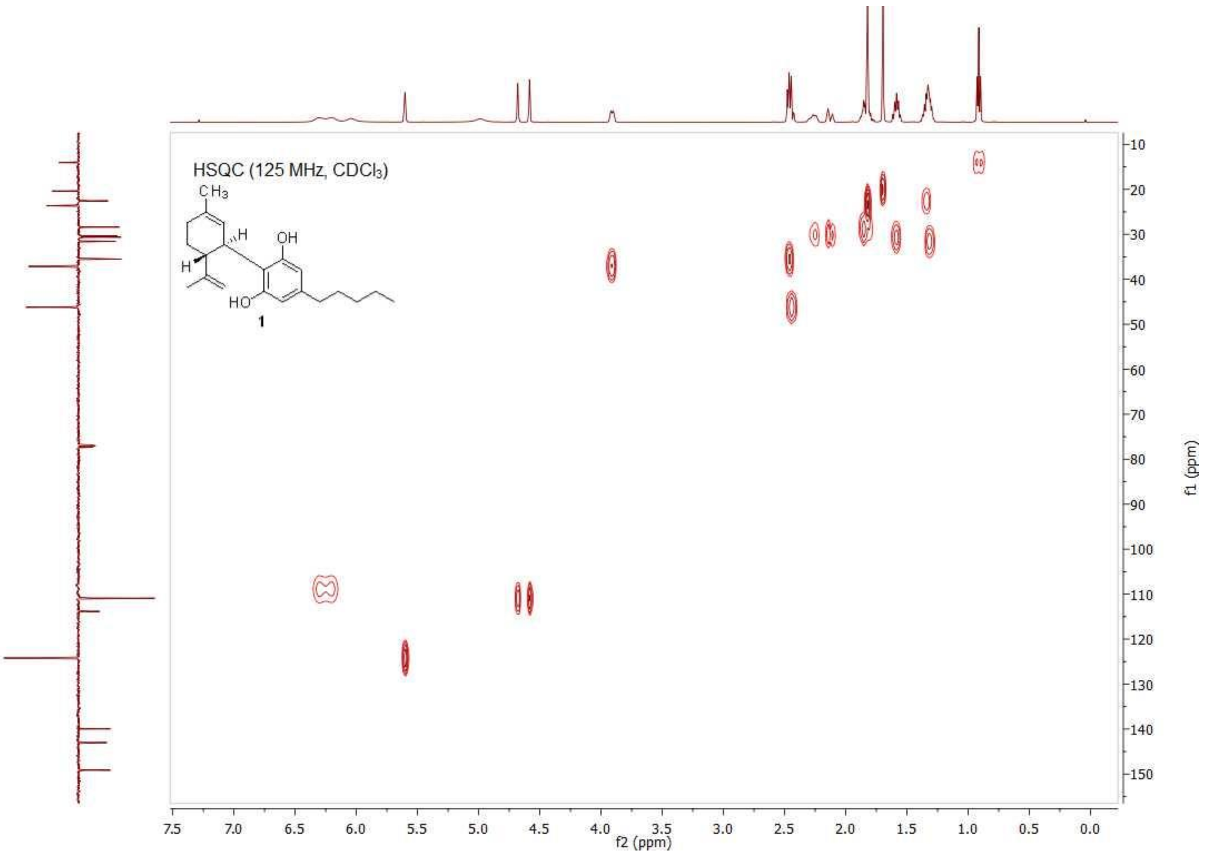
HSQC (125 MHz, CDCl3)
CH3
H
OH
H
HO
1
f2 (ppm)
f1 (ppm)

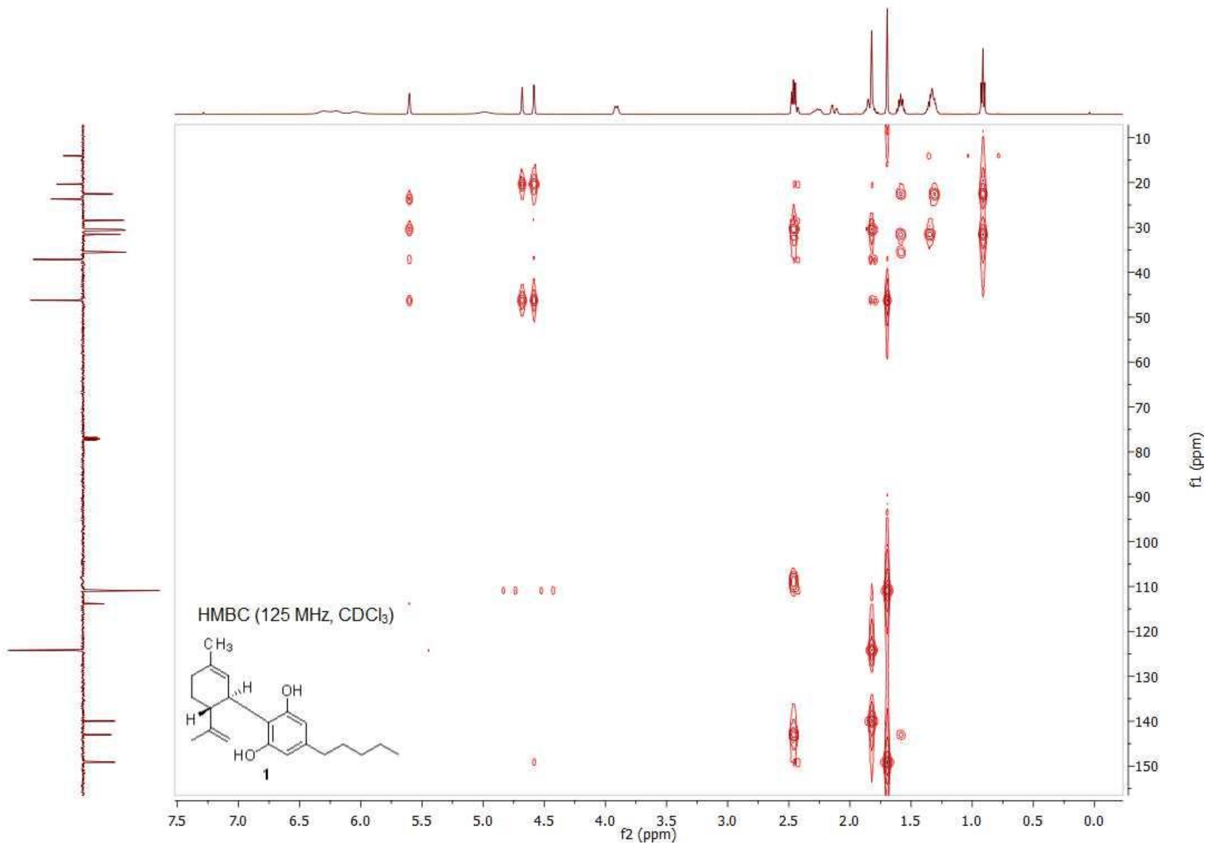
HMBC (125 MHz, CDCl3)
CH3
H
H
OH
HO
1
f2 (ppm)
f1 (ppm)

NMR spectra of compound 8

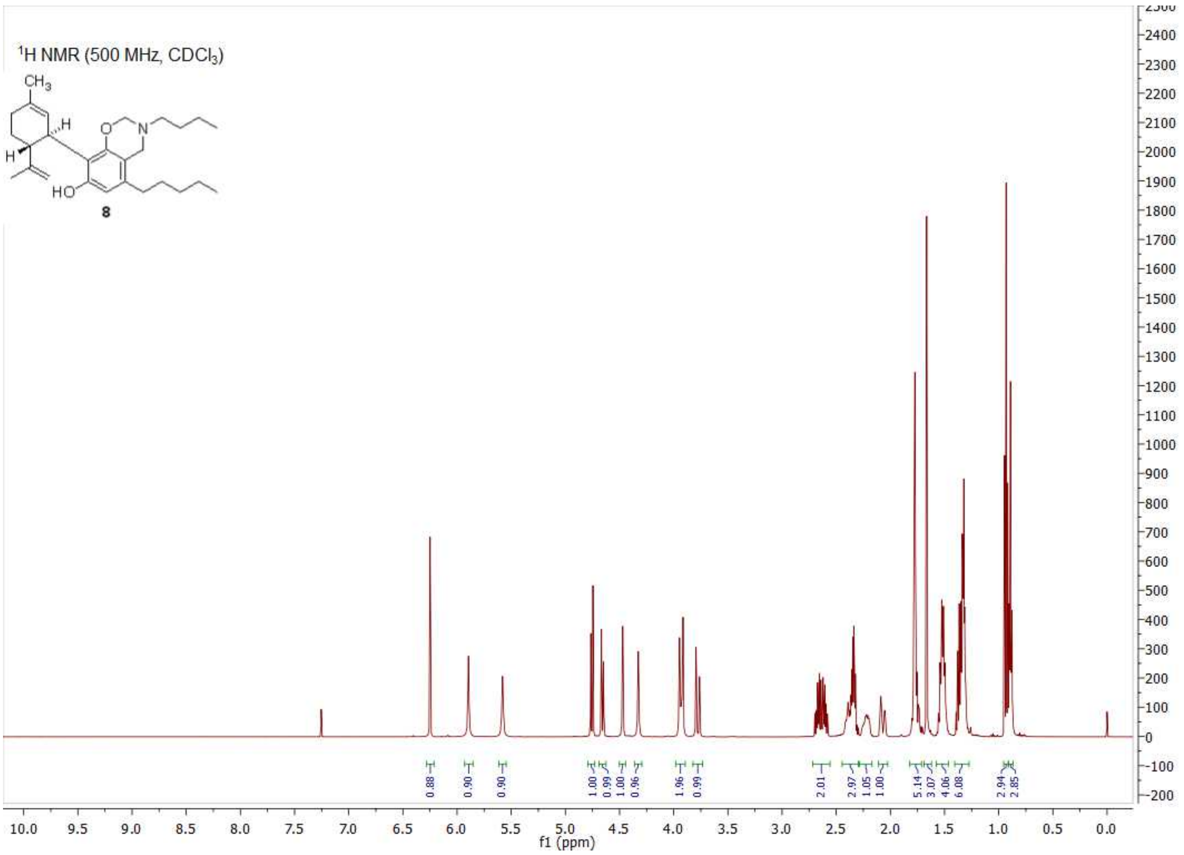

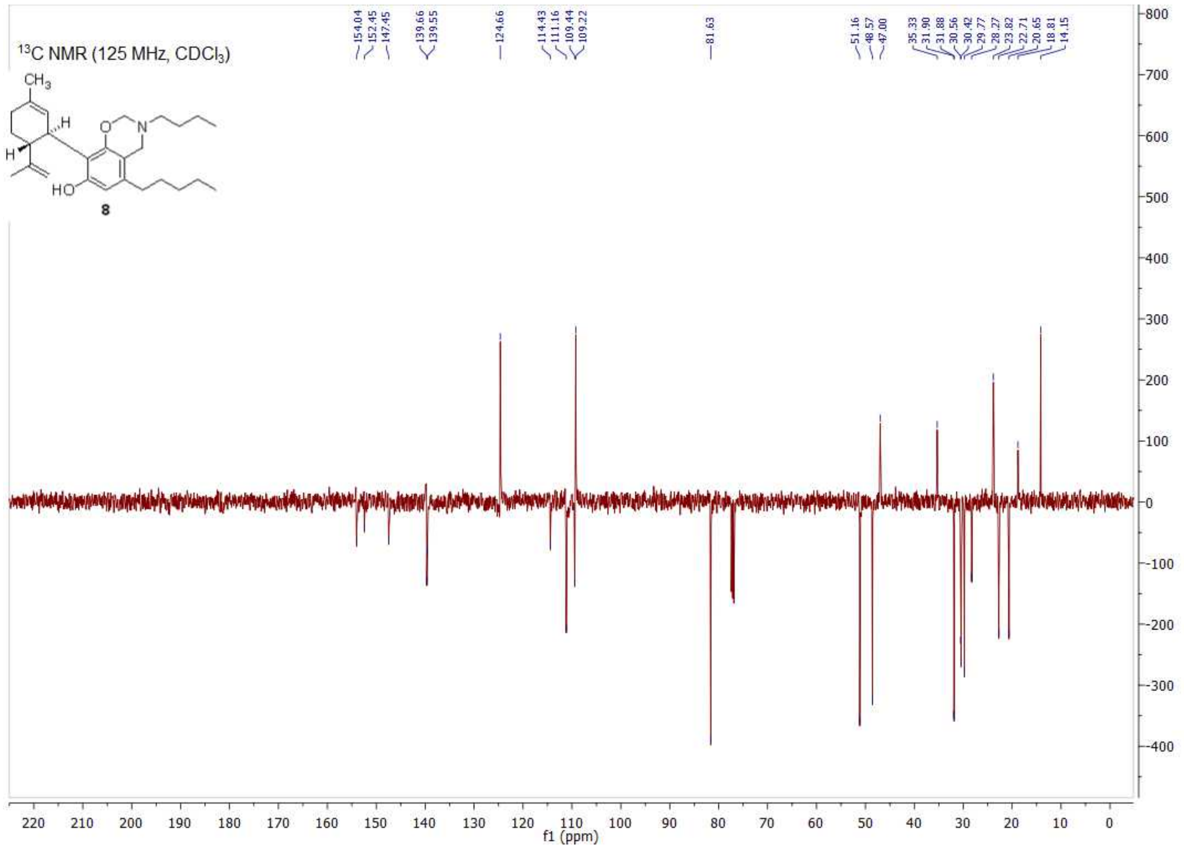

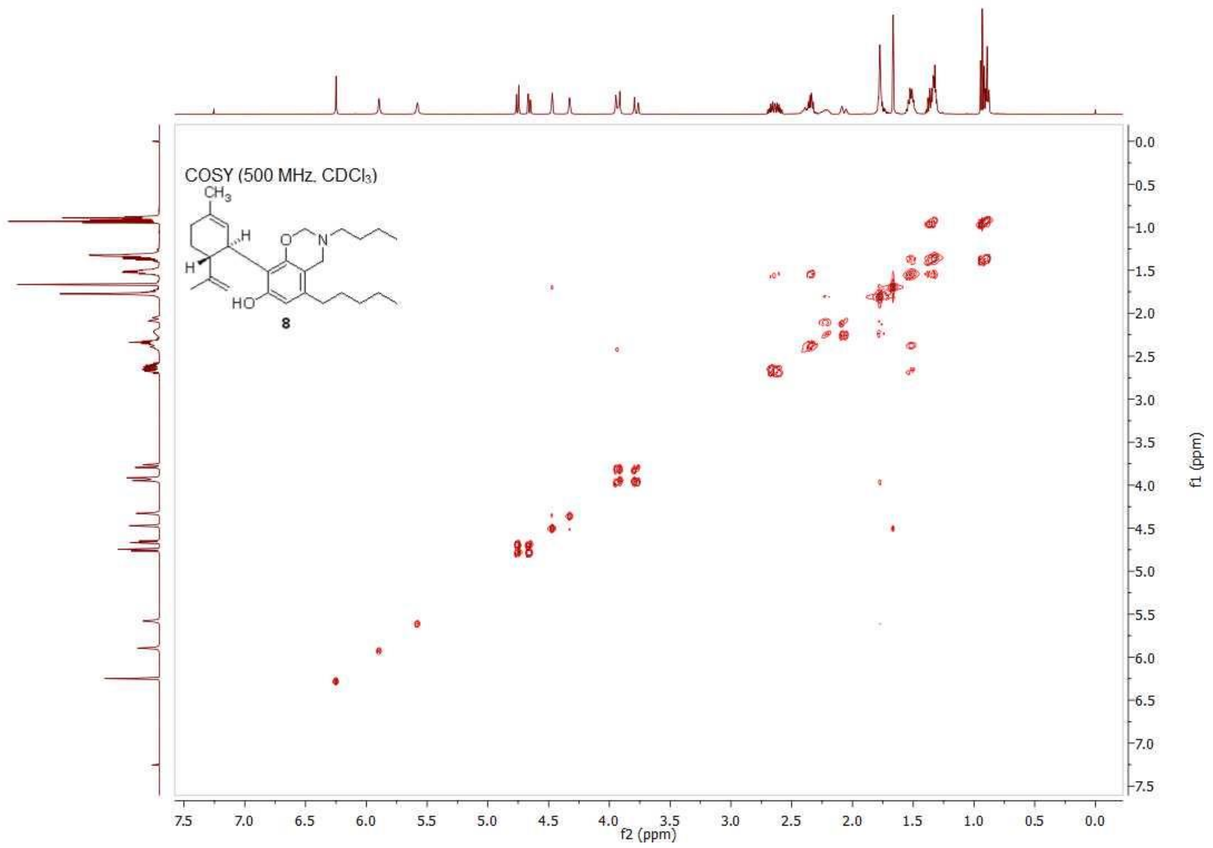
COSY (500 MHz, CDCl3)
CH3
H
O
N
H
HO
8
f2 (ppm)
f1 (ppm)

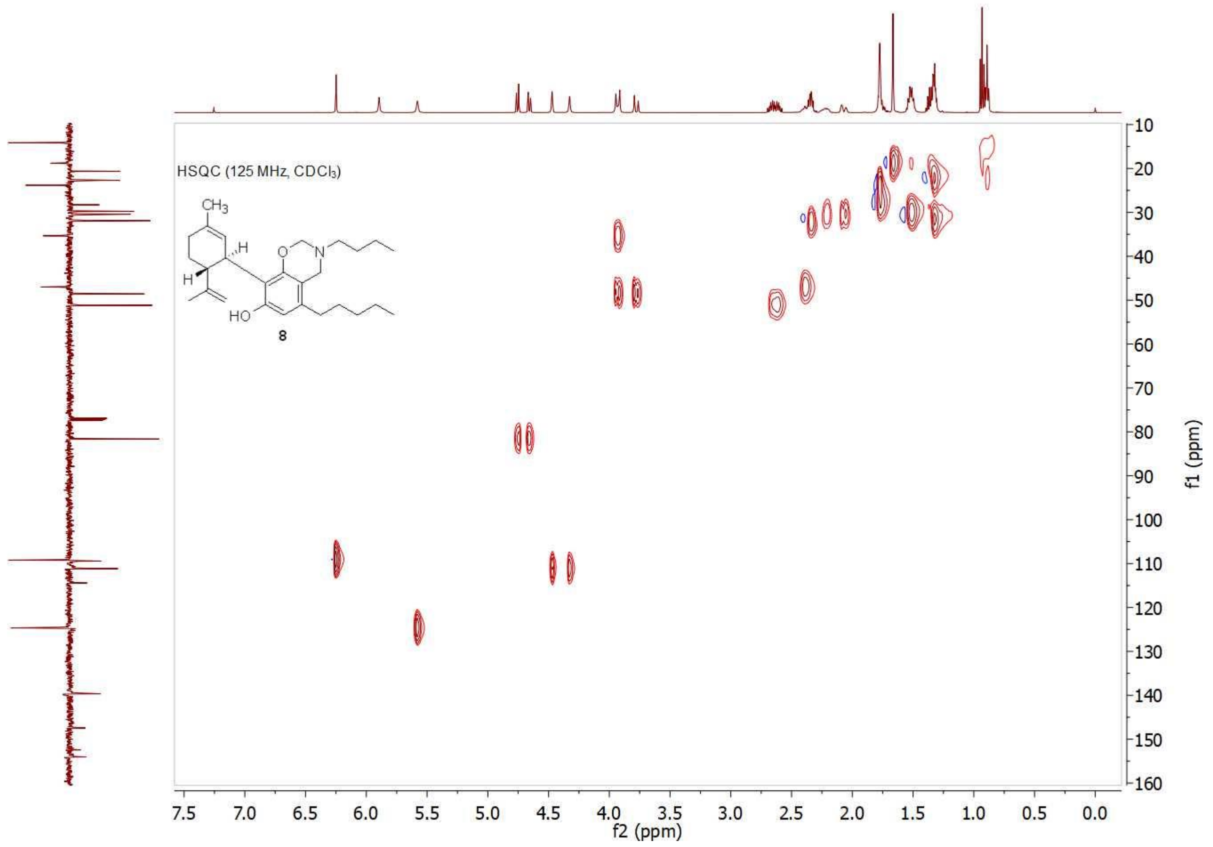
HSQC (125 MHz, CDCl3)
CH3
H
O
N
H
HO
8
f2 (ppm)
f1 (ppm)

NMR spectra of compound 9

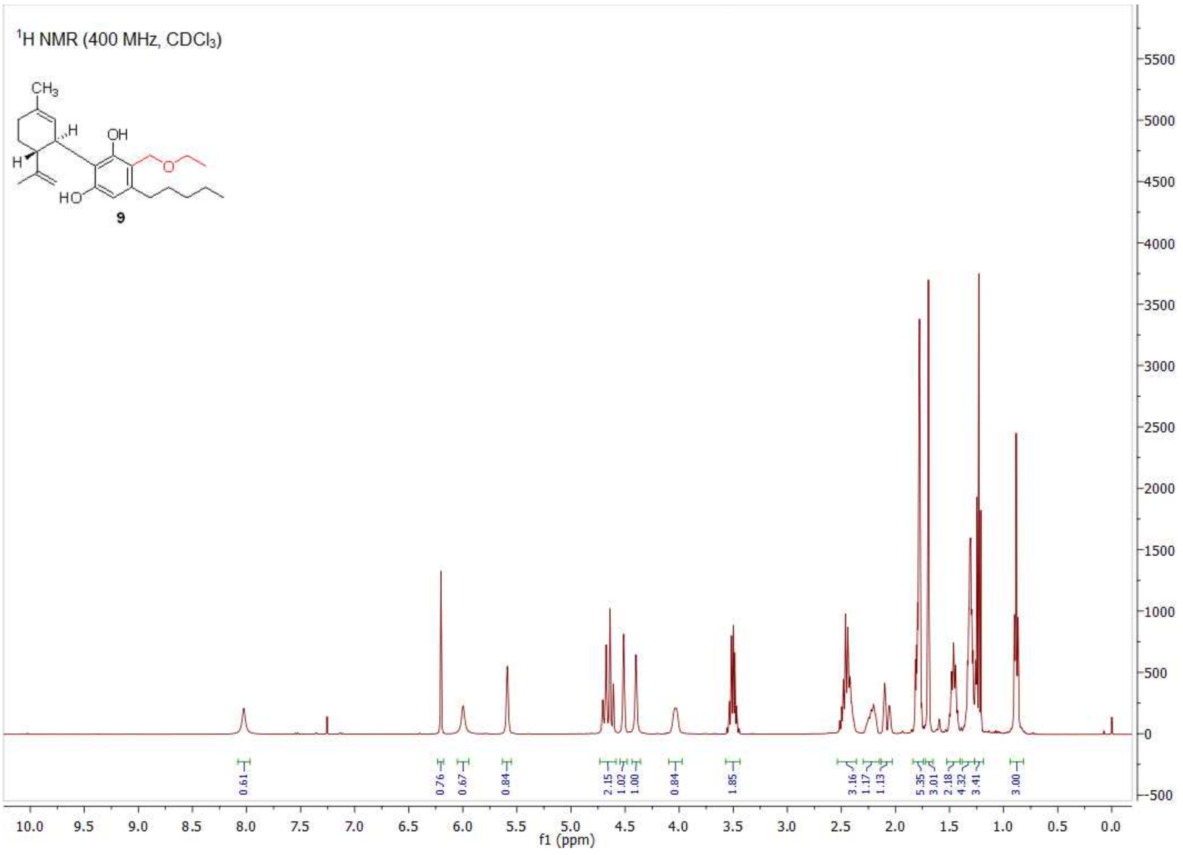

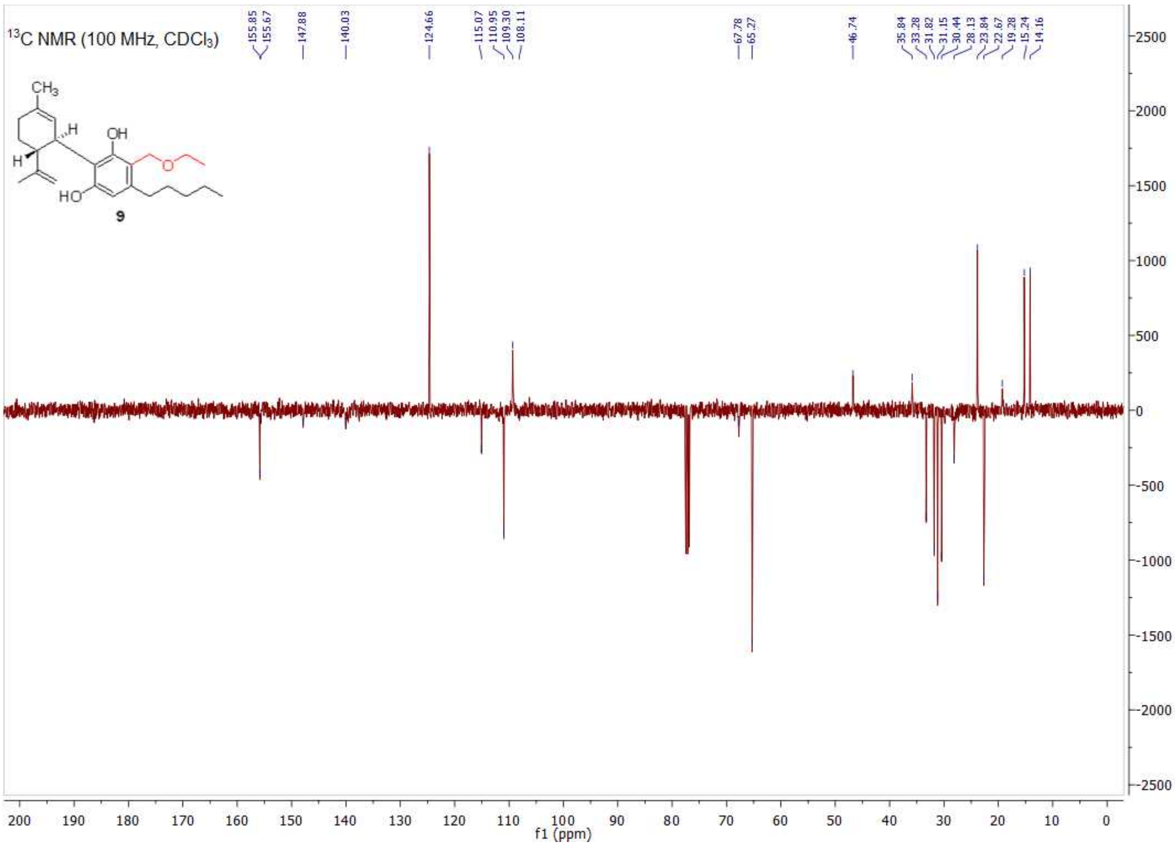

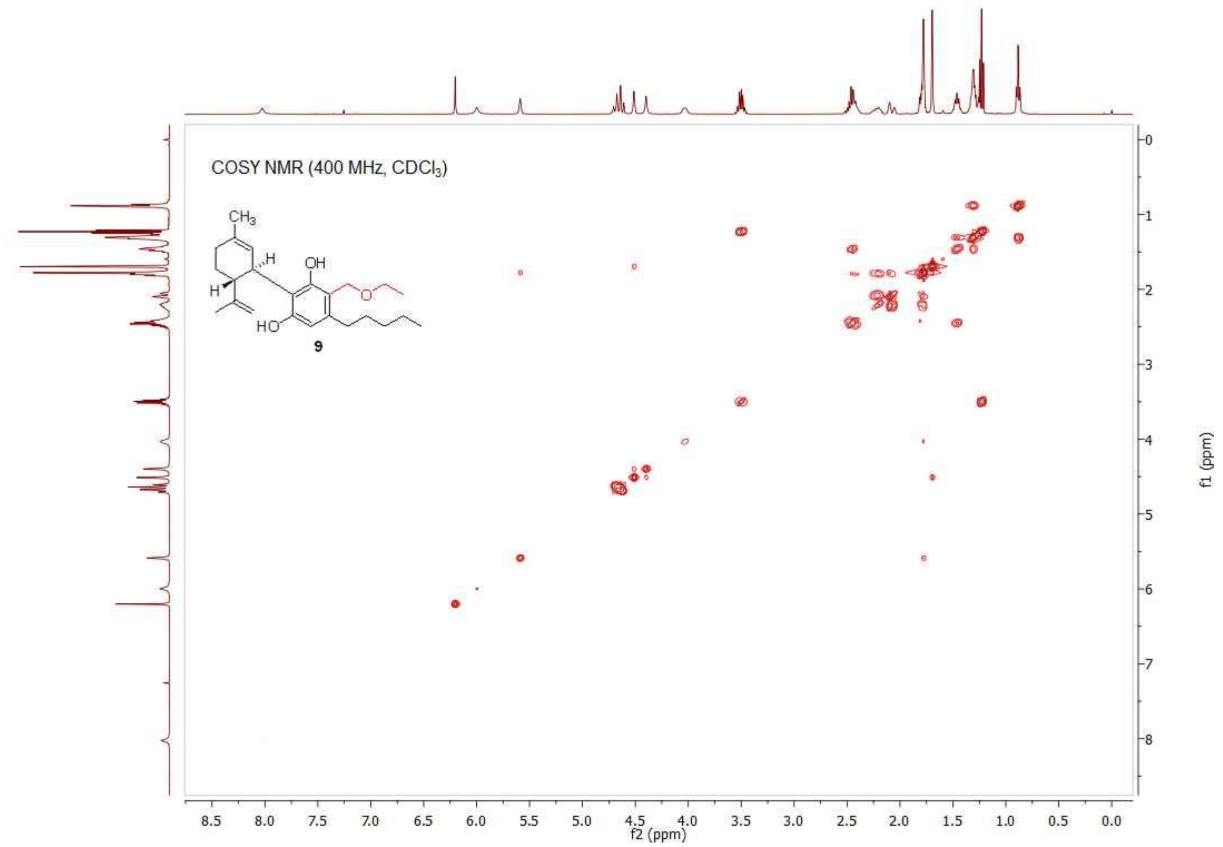
COSY NMR (400 MHz, CDCl3)
CH3
H
OH
H
HO
9
f2 (ppm)
f1 (ppm)

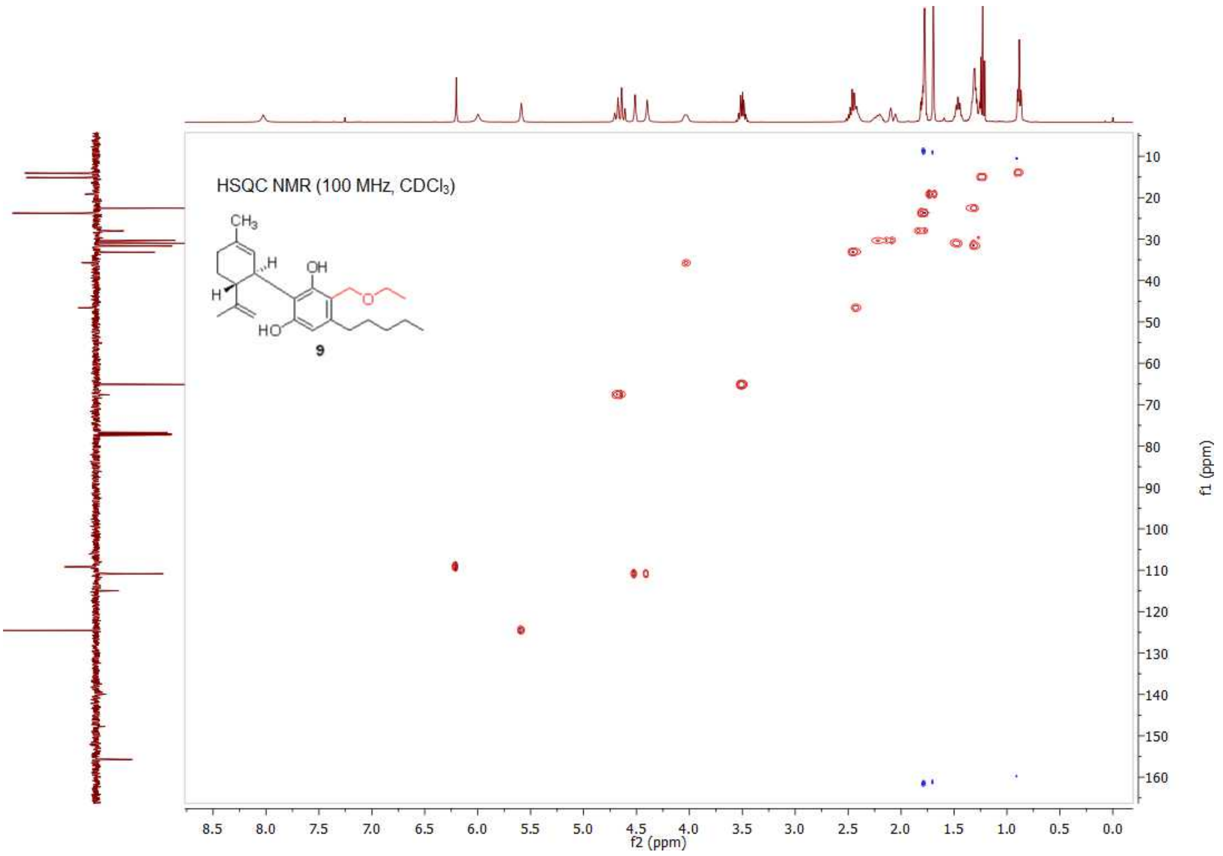
HSQC NMR (100 MHz, CDCl3)
CH3
H
OH
H
HO
9
f2 (ppm)
f1 (ppm)

NMR spectra of compound 10

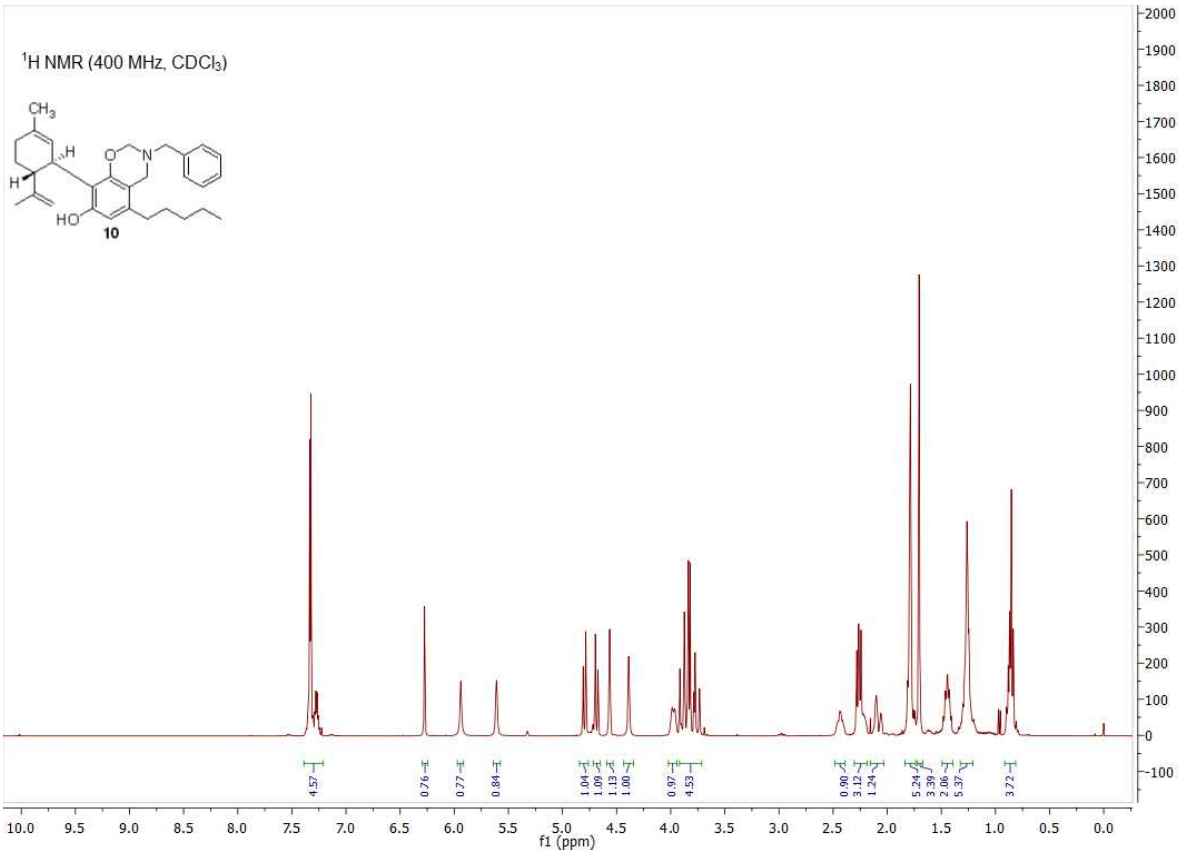

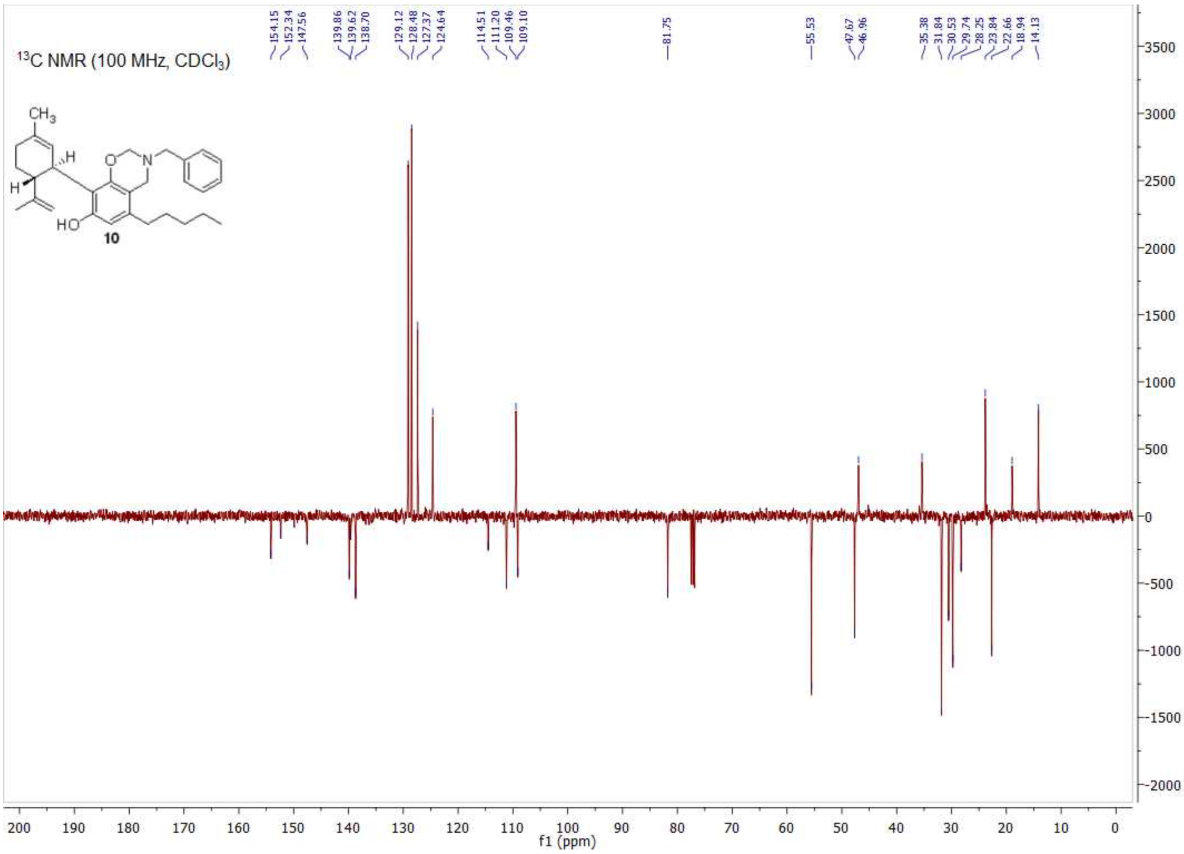

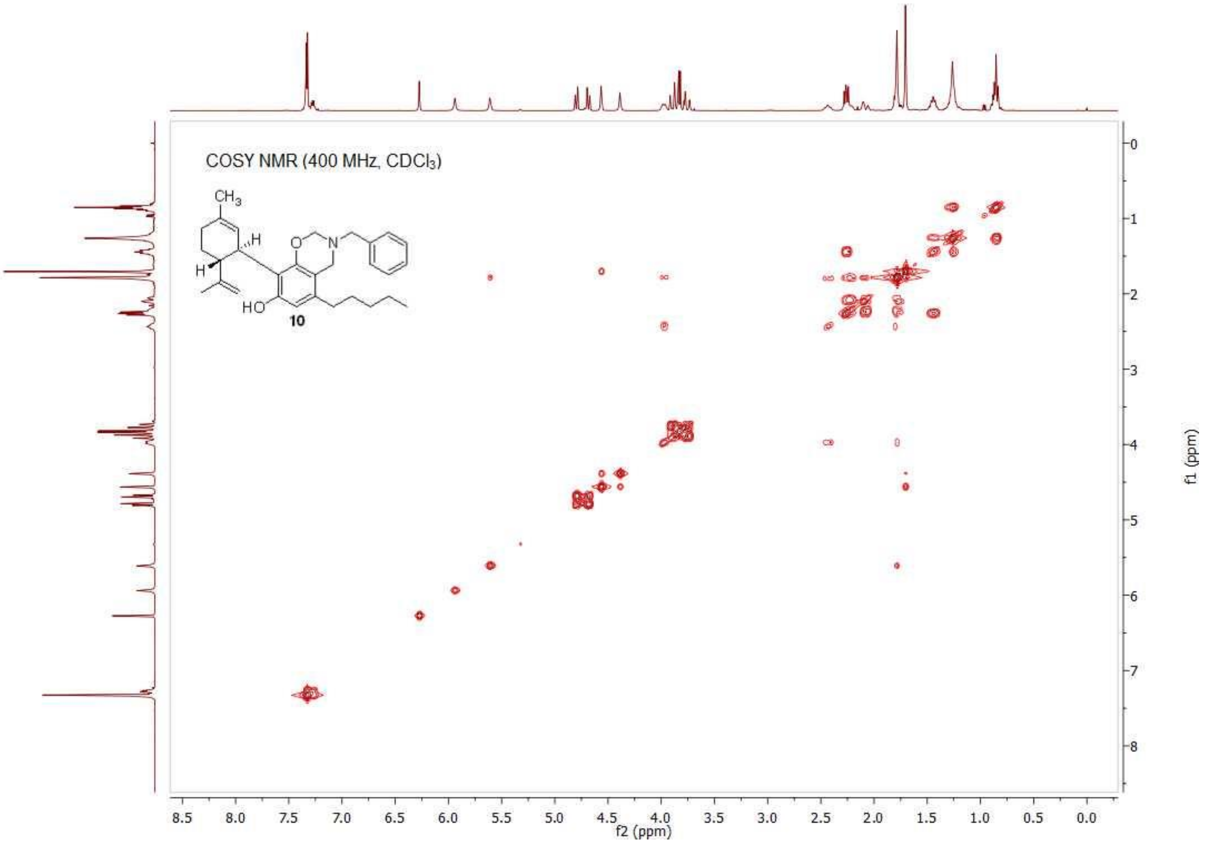
COSY NMR (400 MHz, CDCl3)
CH3
H
H
O
N
HO
10
f2 (ppm)
f1 (ppm)

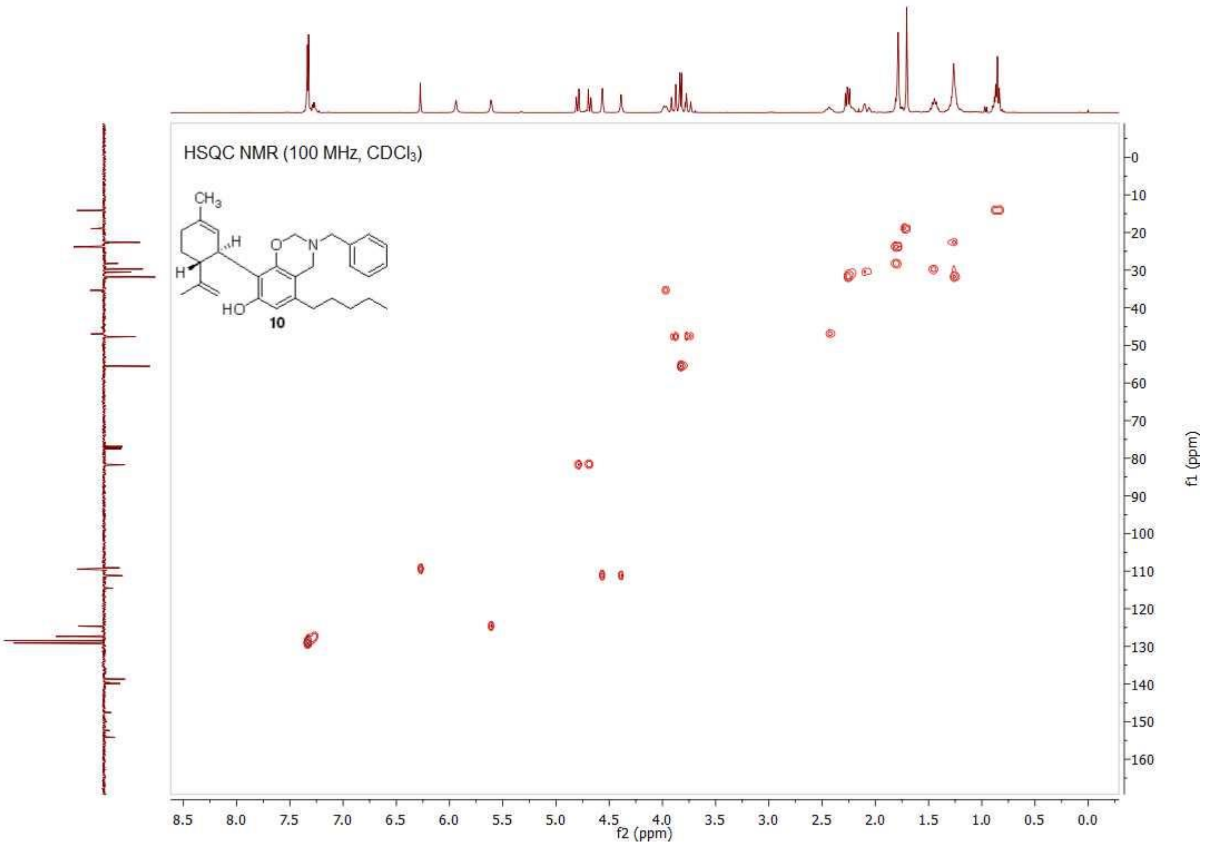
HSQC NMR (100 MHz, CDCl3)
CH3
H
H
O
N
HO
10
f2 (ppm)
f1 (ppm)

NMR spectra of compound 11

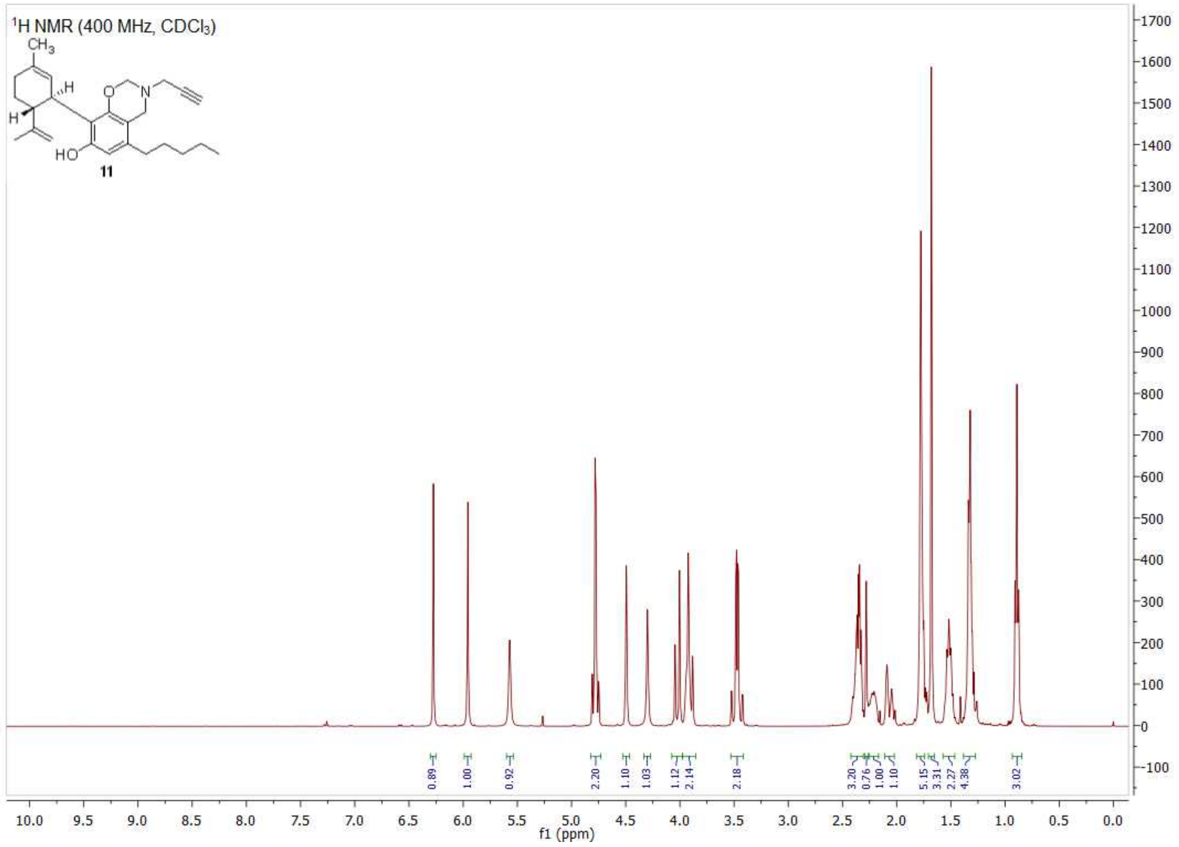

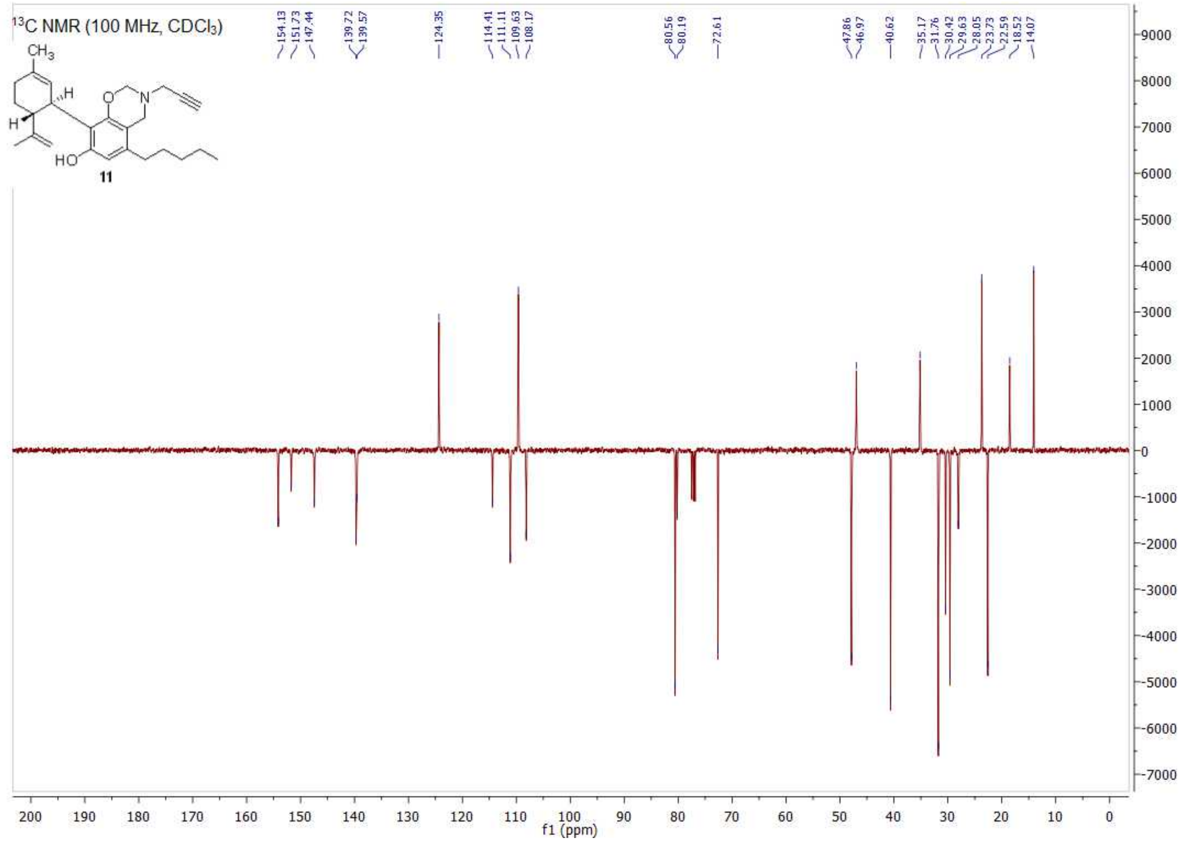

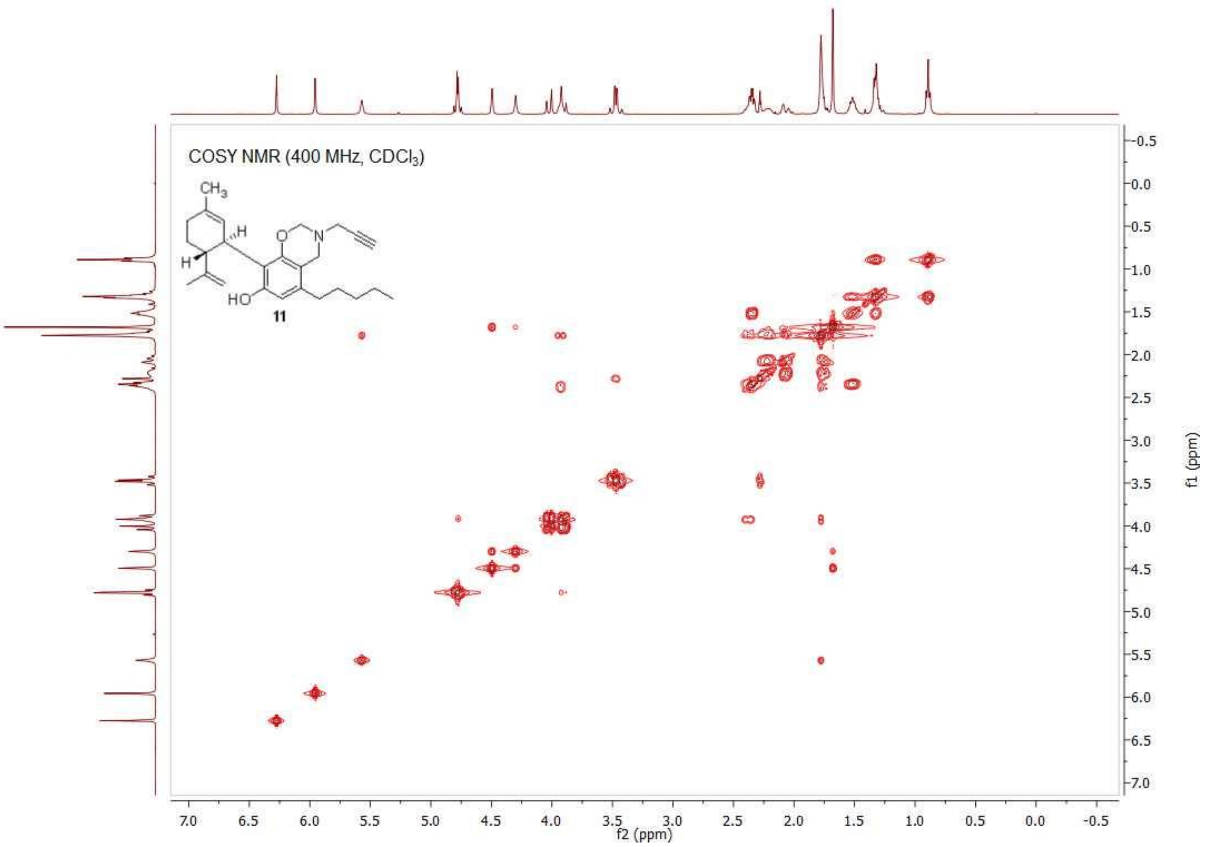
COSY NMR (400 MHz, CDCl3)
11
f2 (ppm)
f1 (ppm)

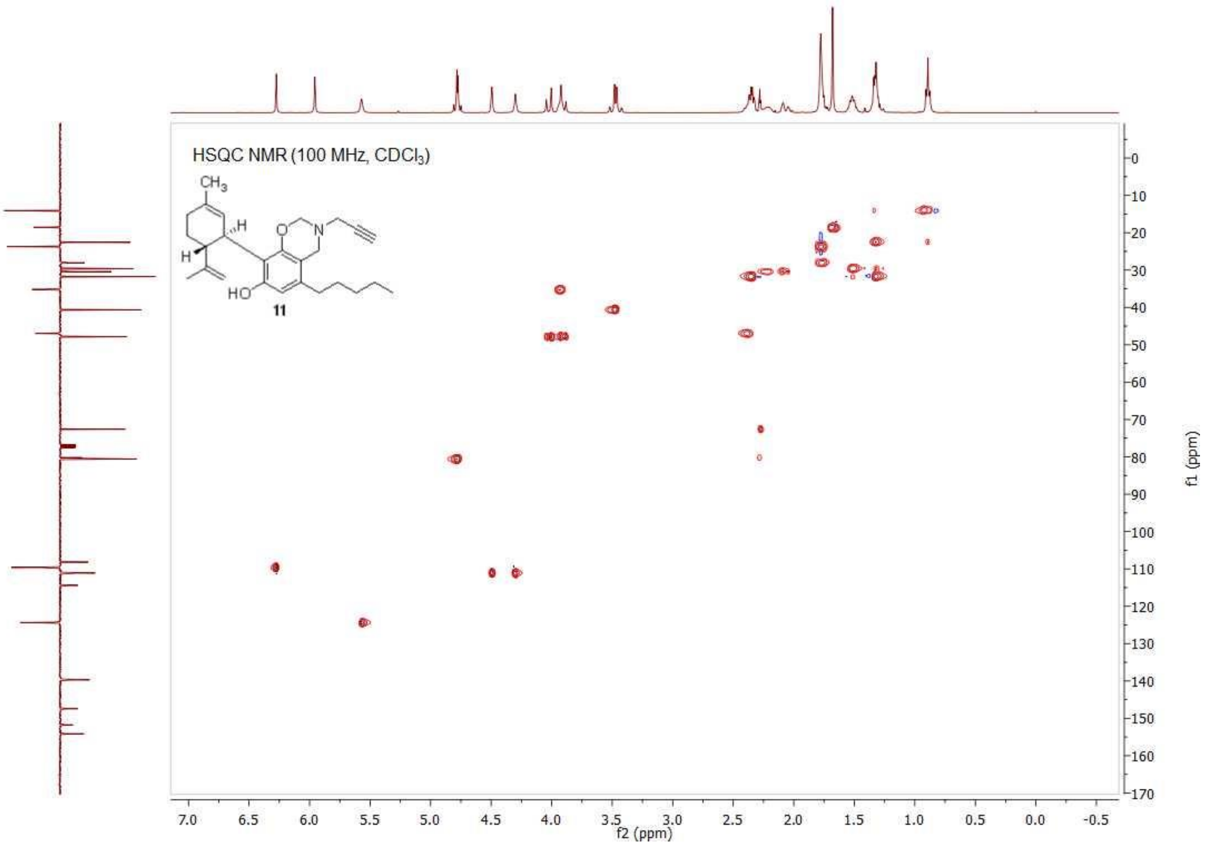
HSQC NMR (100 MHz, CDCl3)
11
f2 (ppm)
f1 (ppm)

NMR spectra of compound 12

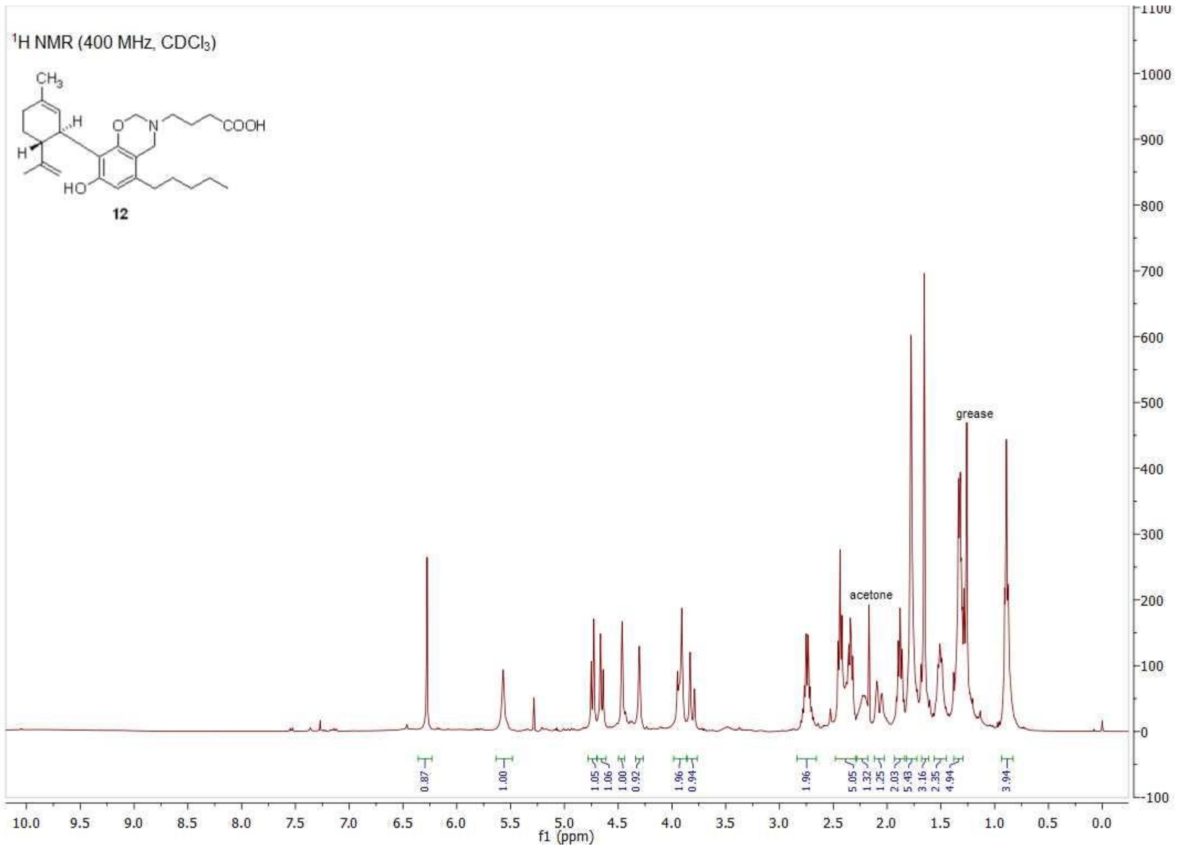

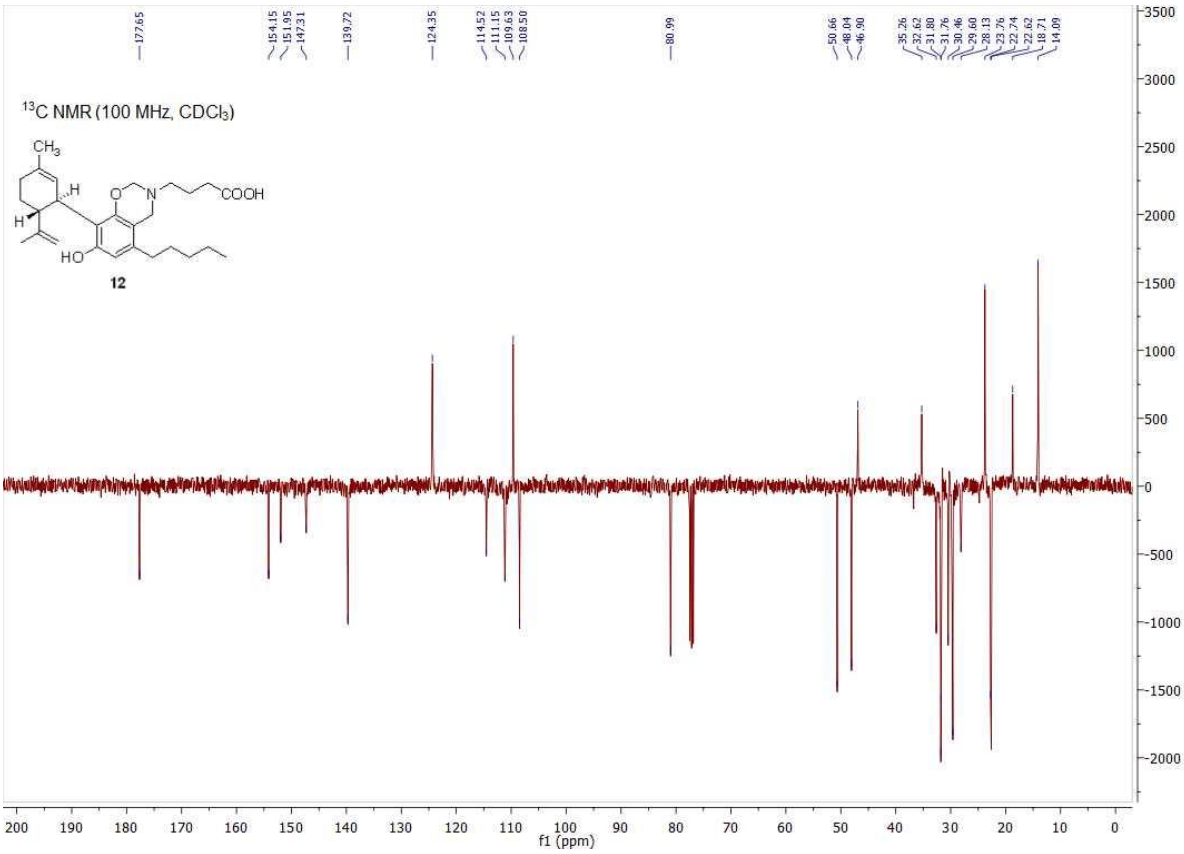

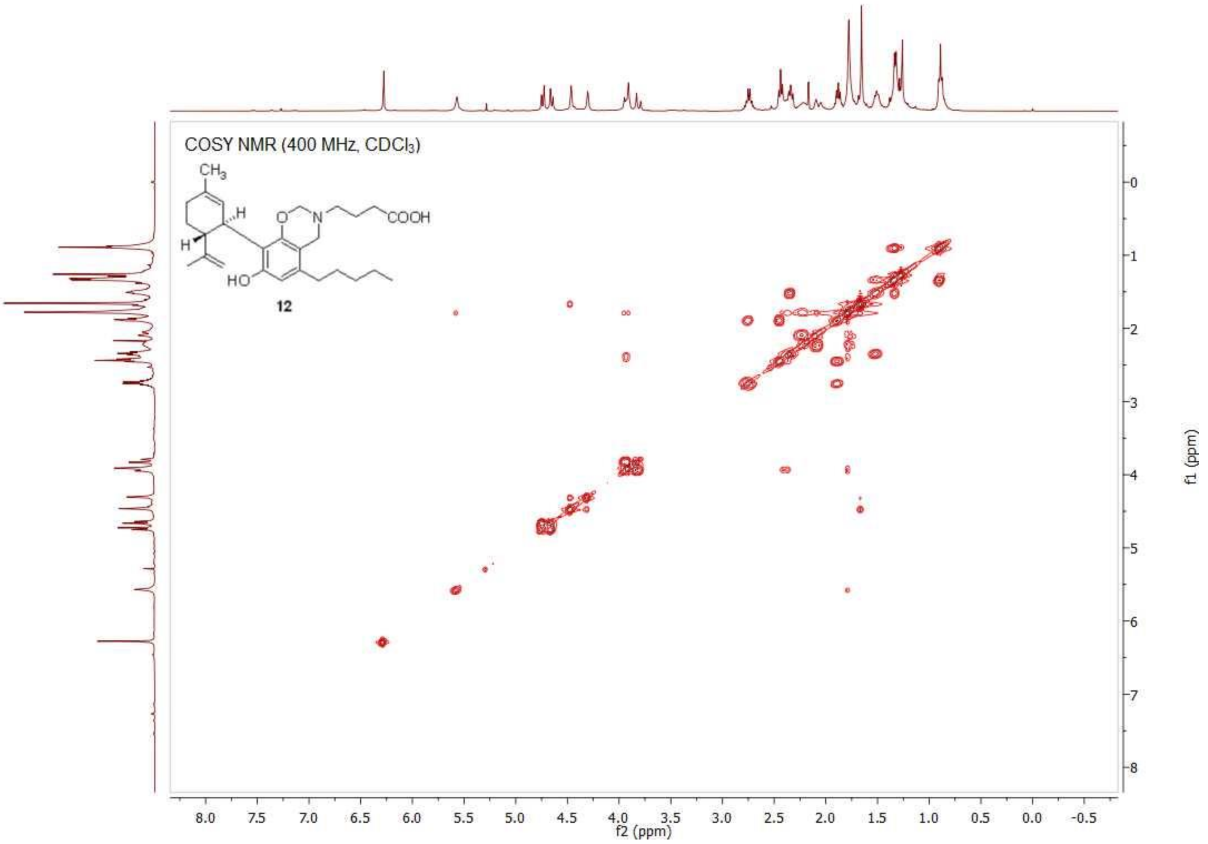
COSY NMR (400 MHz, CDCl3)
CH3
H
H
O
N
COOH
HO
12
f2 (ppm)
f1 (ppm)

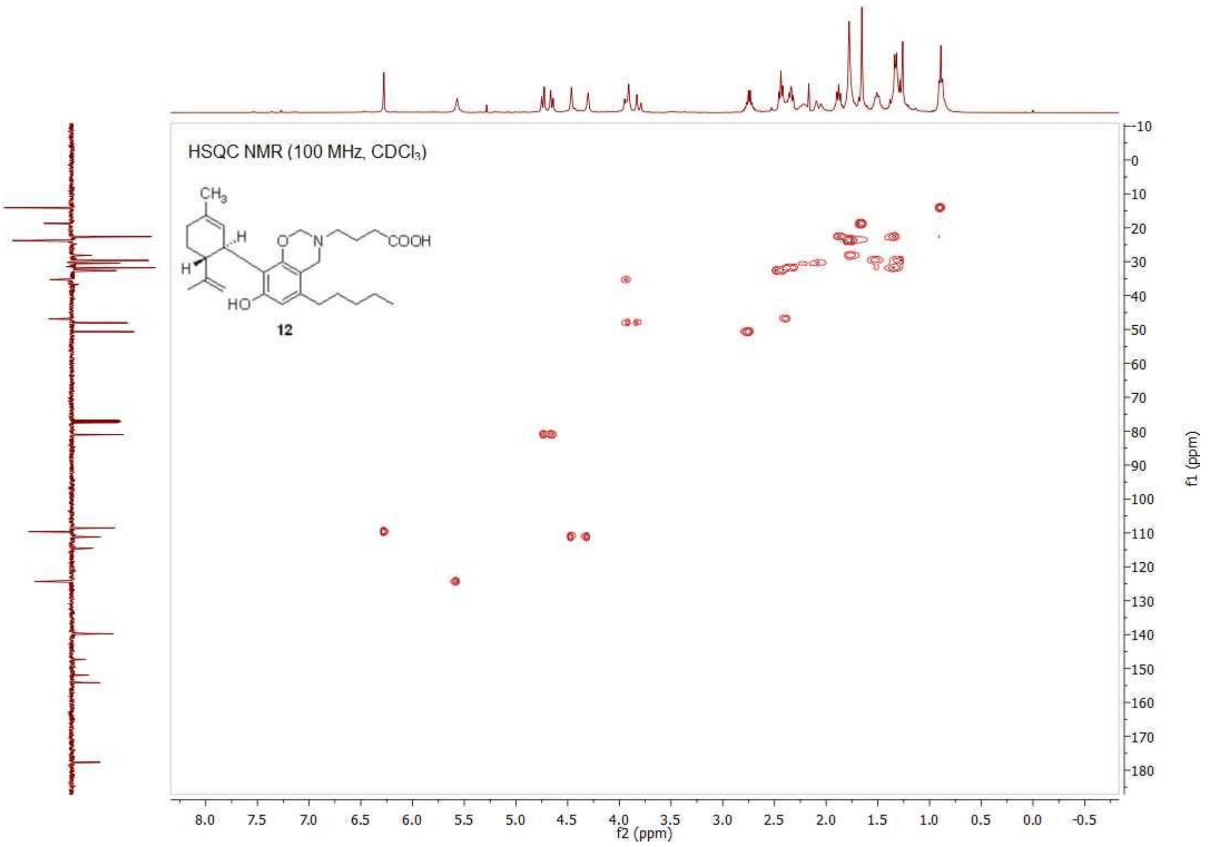
HSQC NMR (100 MHz, CDCl3)
CH3
H
H
O
N
COOH
HO
12
f2 (ppm)
f1 (ppm)

NMR spectra of compound 13

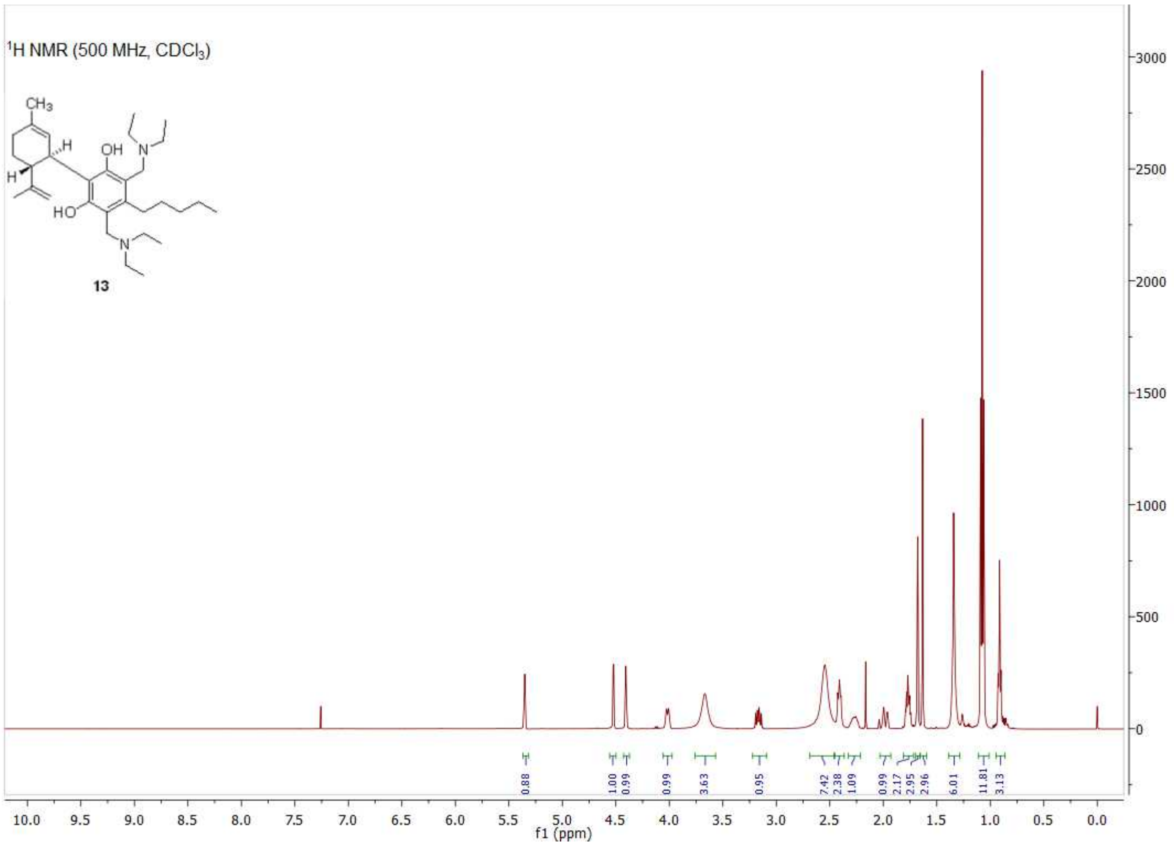

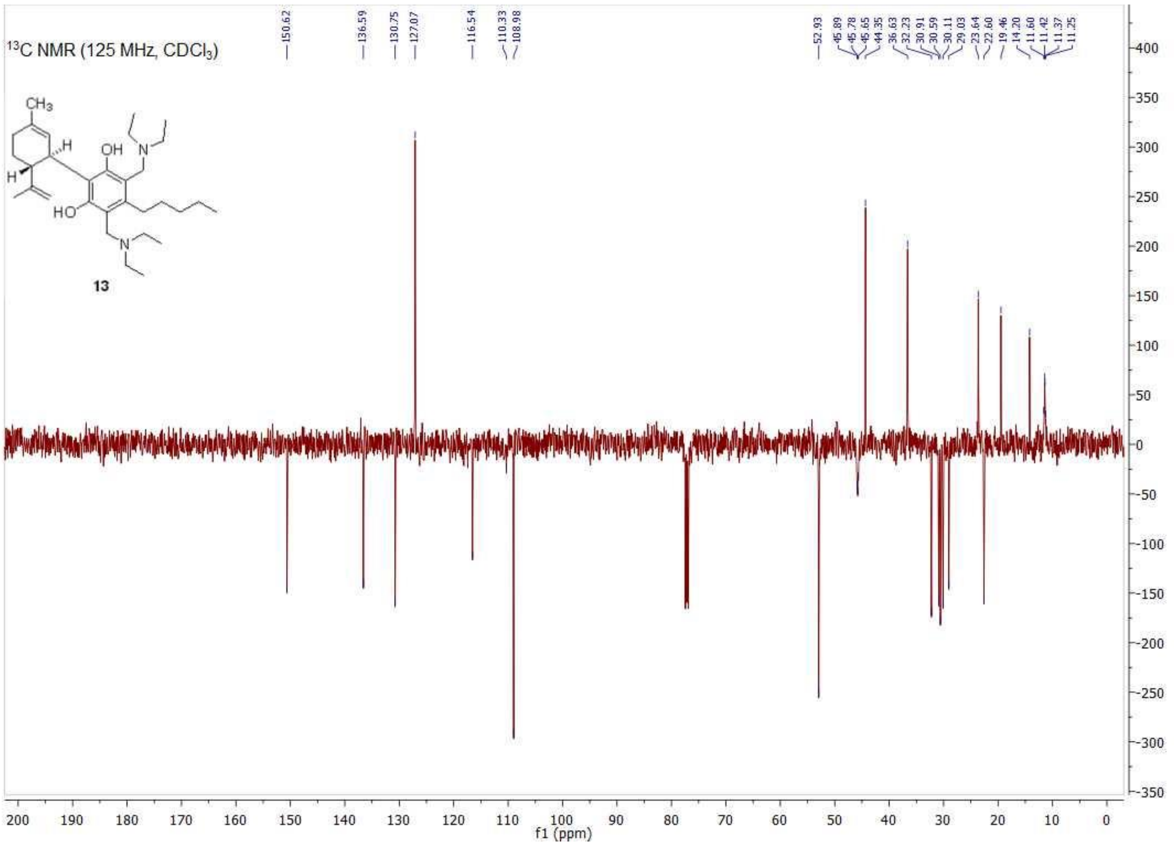

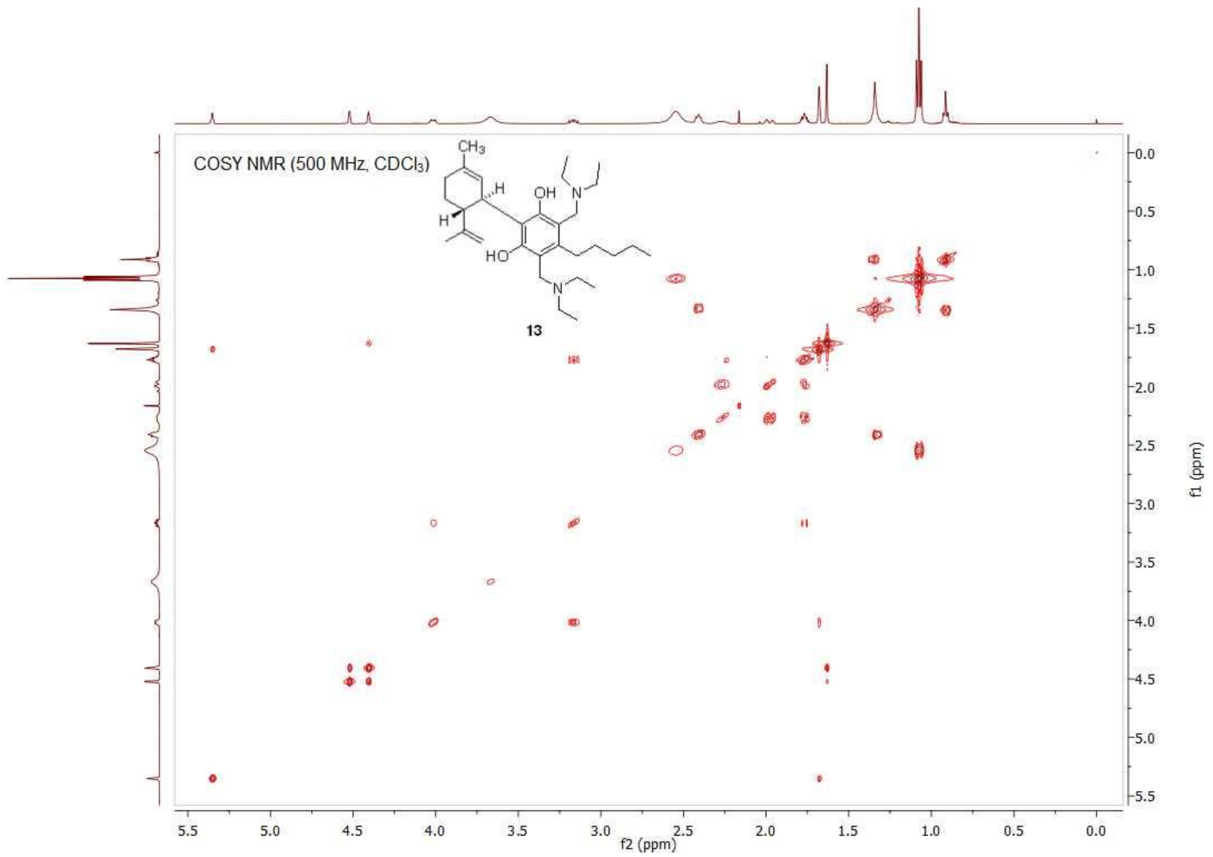
COSY NMR (500 MHz, CDCl3)
CH3
H
OH
N
H
HO
N
13
f2 (ppm)
f1 (ppm)

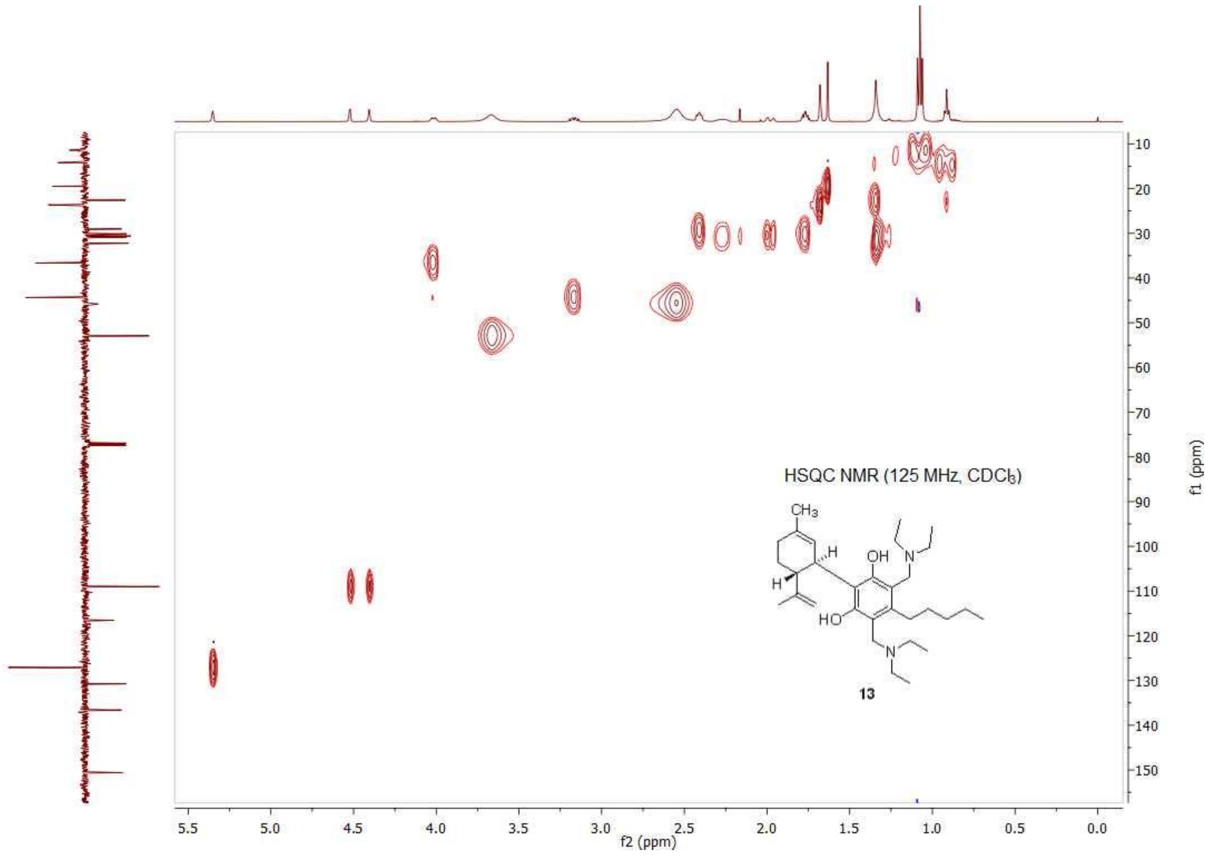
HSQC NMR (125 MHz, CDCl3)
CH3
H
OH
N
H
HO
N
13
f2 (ppm)
f1 (ppm)

NMR spectra of compound 14

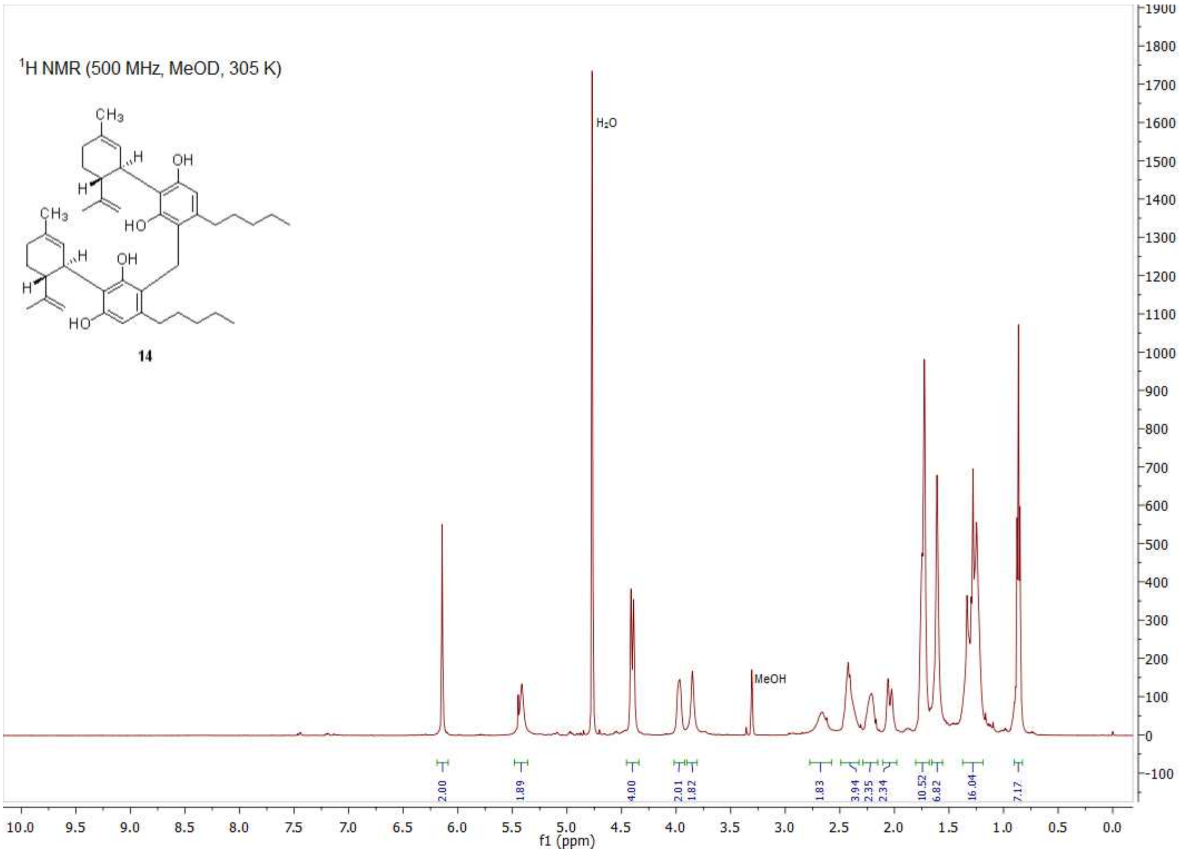

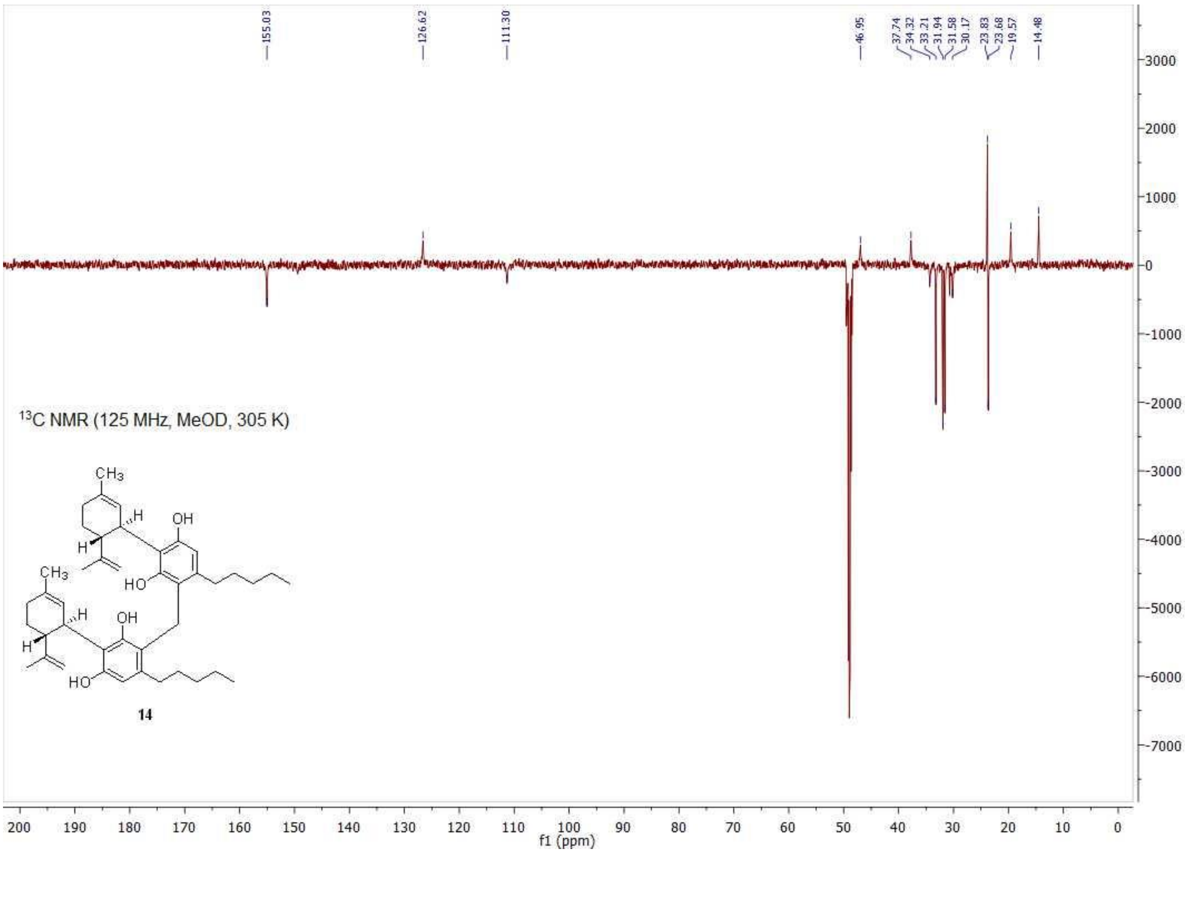

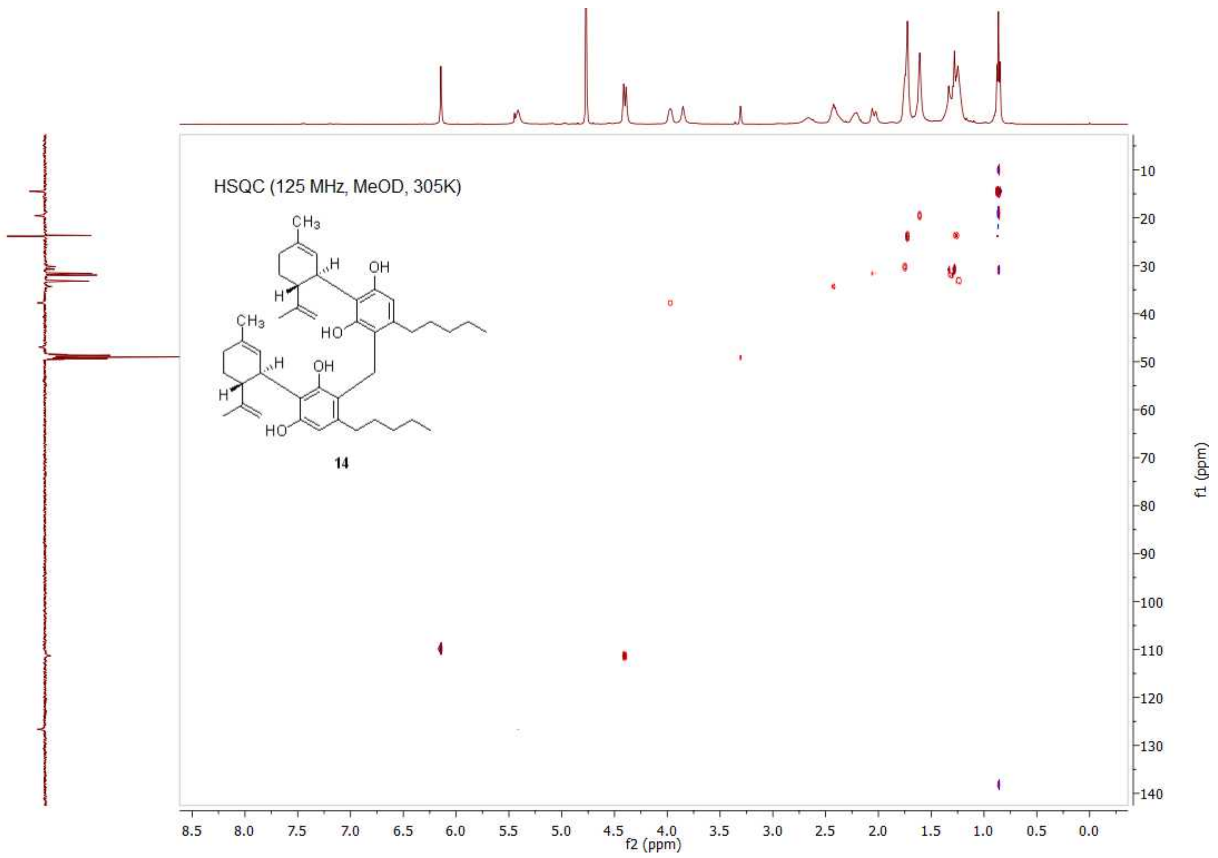

HSQC (125 MHz, MeOD, 305K)
CH3
OH
HO
OH
HO
14
f1 (ppm)
f2 (ppm)

4. NMR data of CBG and its derivatives

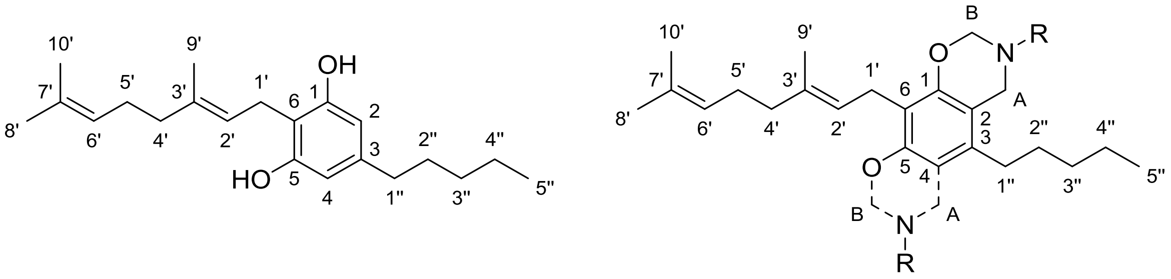

Figure S3. Numbering of CBG and its derivatives

NMR data of CBG:

^{1}H NMR (500 MHz, $CDCl_3$): δ (ppm) 6.24 (s, 2H aromatic C*H*), 5.29-5.24 (m, 1H, H-2'C*H*), 5.15 (s, 2H, O*H*), 5.07-5.02 (m, 1H, H-6' C*H*), 3.39 (d, 2H, J = 7.1 Hz, H-1' CH_2), 2.46-2.41 (m, 2H, H-1" CH_2), 2.13-2.07 (m, 2H, H-5' CH_2), 2.07-2.02 (m, 2H, H-4' CH_2), 1.80 (s, 3H, H-9' CH_3), 1.58, 1.67 (2s, 6H, H-8' and H-10' CH_3), 1.57-1.51 (m, 2H, H-2" CH_2), 1.36-1.24 (m, 4H, H-3" and H-4" CH_2), 0.88 (t, 3H, J = 6.9 Hz, H-5" CH_3); ^{13}C NMR (125 MHz, $CDCl_3$): δ (ppm) 154.9, 142.9, 139.1, 132.2 (4C, quat.), 123.9 (1C, C-6' *C*H), 121.9 (1C, C-2' *C*H), 110.8 (1C, quat.), 108.5 (2C, aromatic *C*H), 39.8 (1C, C-4' *C*H_2), 35.7 (1C, C-1" *C*H_2), 31.6 (1C, C-3" *C*H_2), 30.9 (1C, C-2" *C*H_2), 26.5 (1C, C-5' *C*H_2), 25.8 (1C, C-8'or C-10' *C*H_3), 22.7 (1C, C-4" *C*H_2), 22.4 (1C, C-1' *C*H_2), 17.8 (1C, C-8'or C-10' *C*H_3), 16.3 (1C, C-9' *C*H_3), 14.1 (1C, C-5" *C*H_3).

NMR spectra of CBG

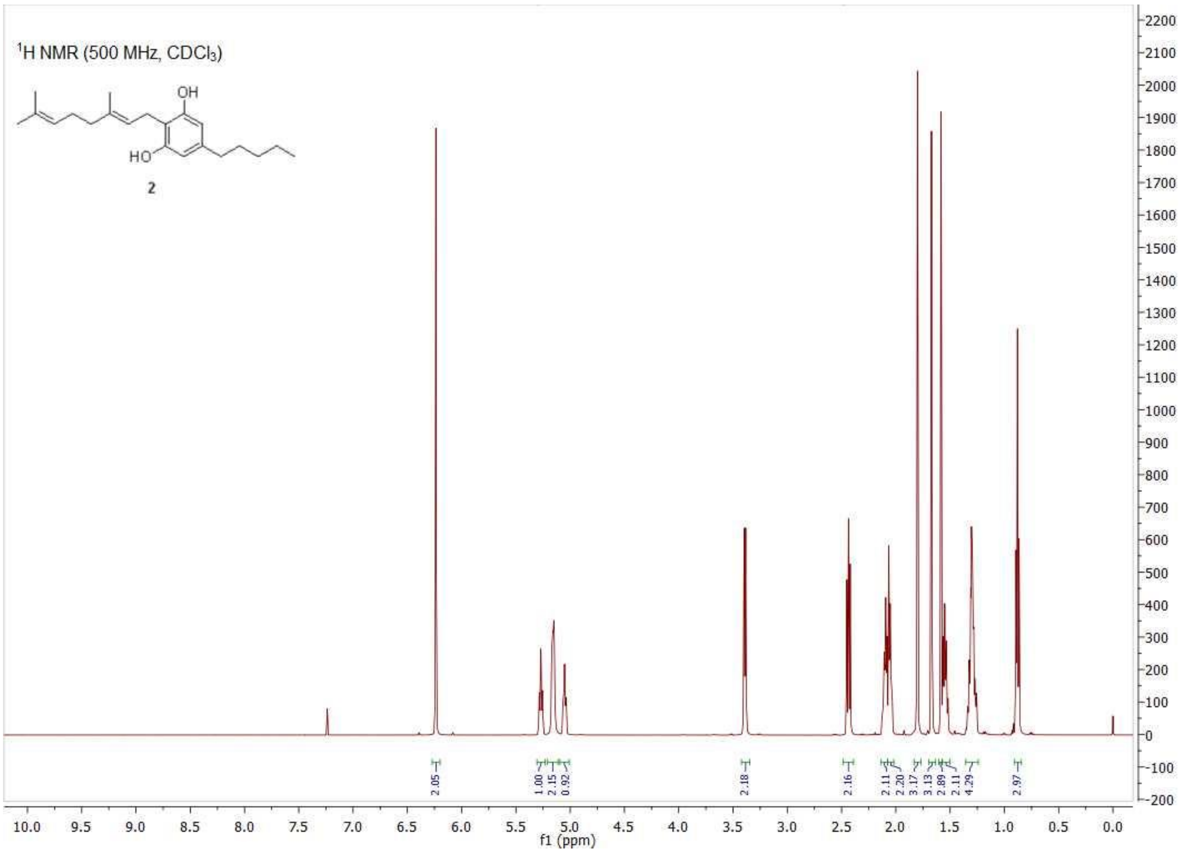

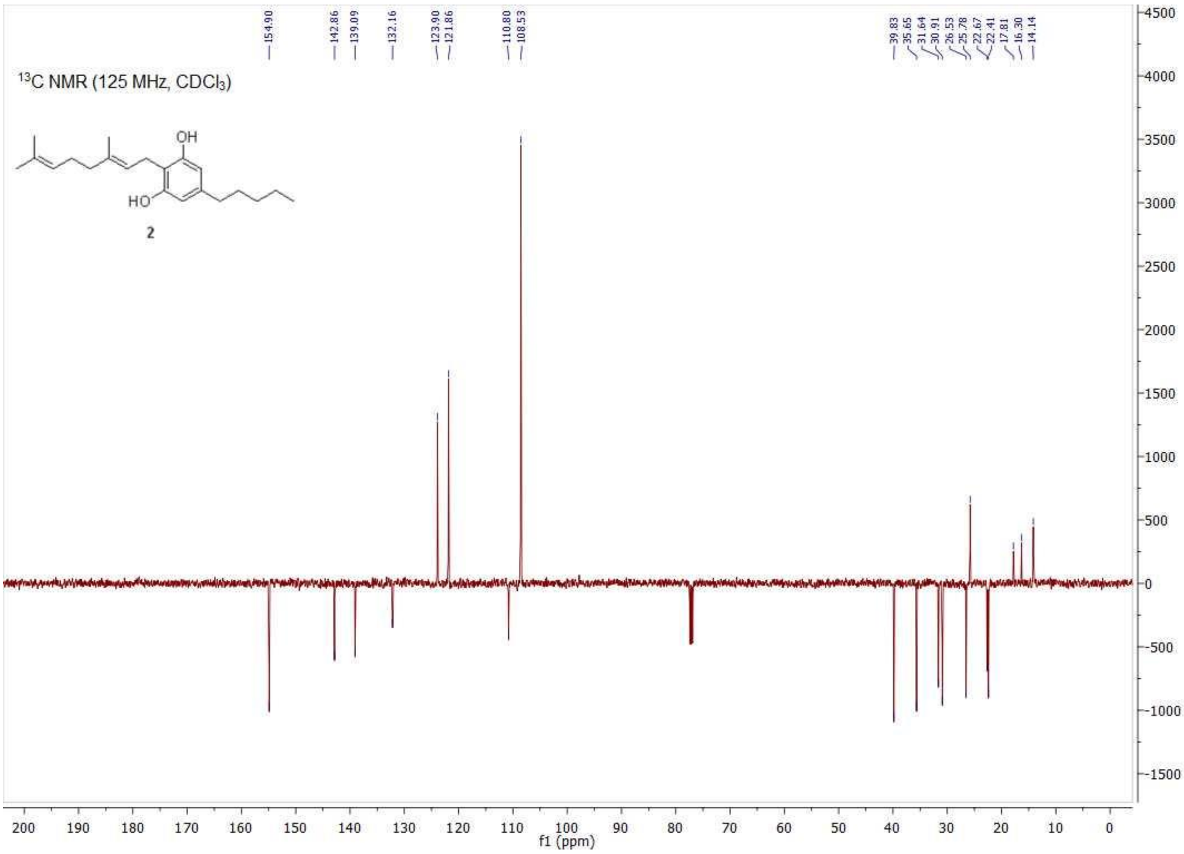

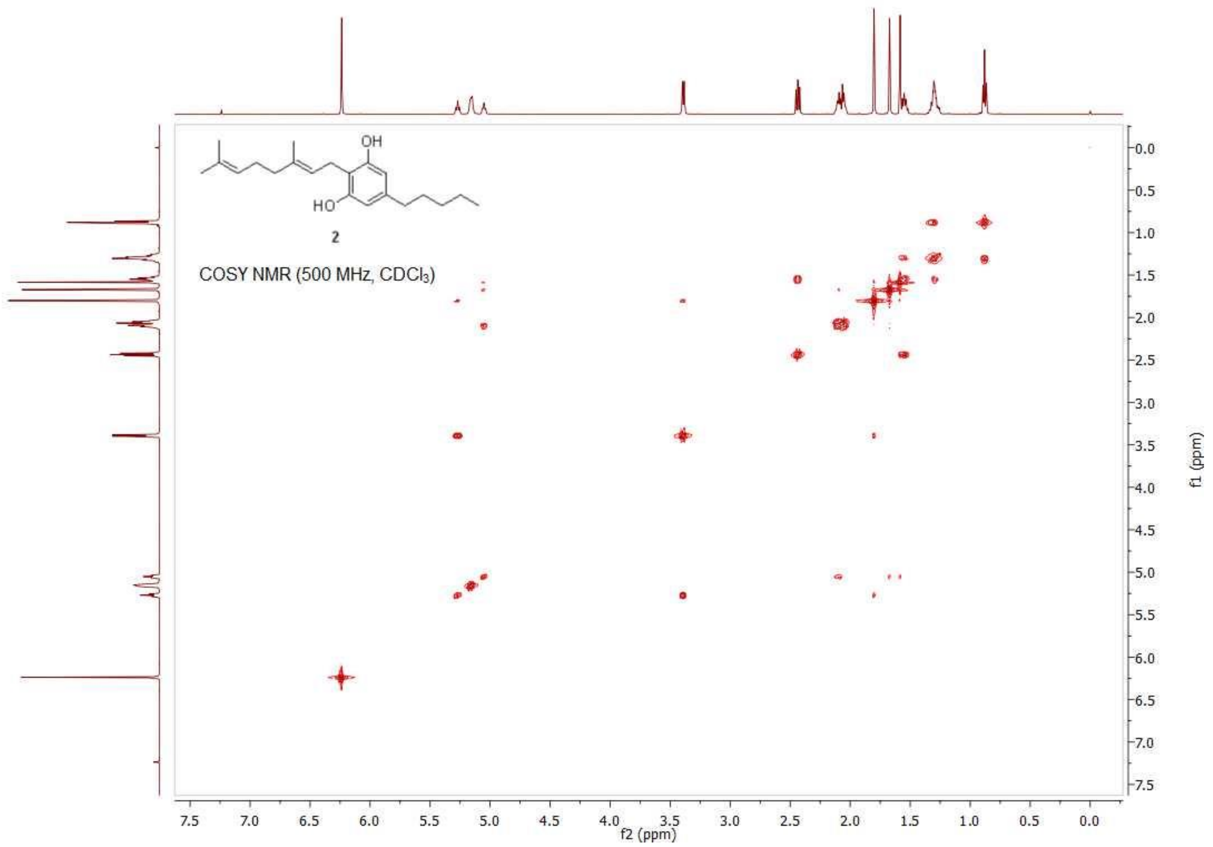
OH
HO
2
COSY NMR (500 MHz, CDCl3)
f1 (ppm)
f2 (ppm)

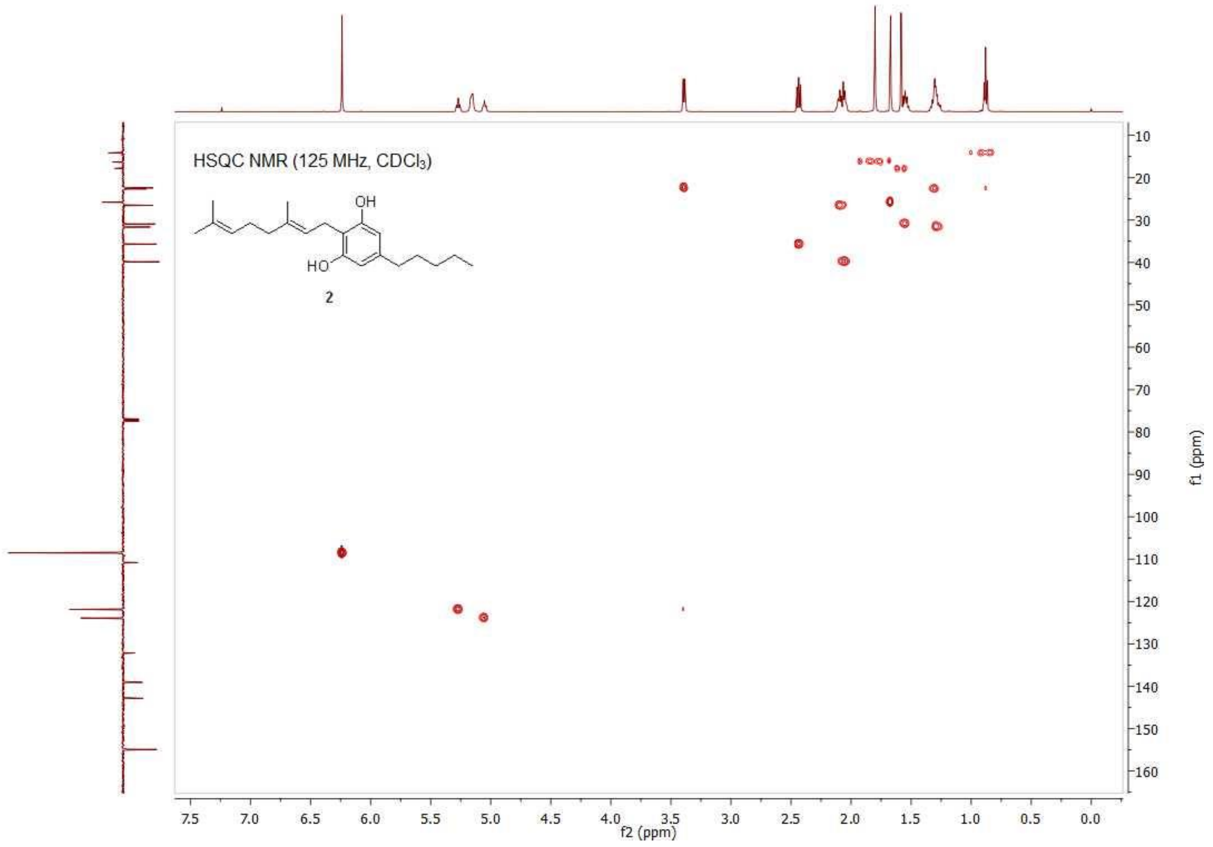
HSQC NMR (125 MHz, CDCl3)
OH
HO
2
f1 (ppm)
f2 (ppm)

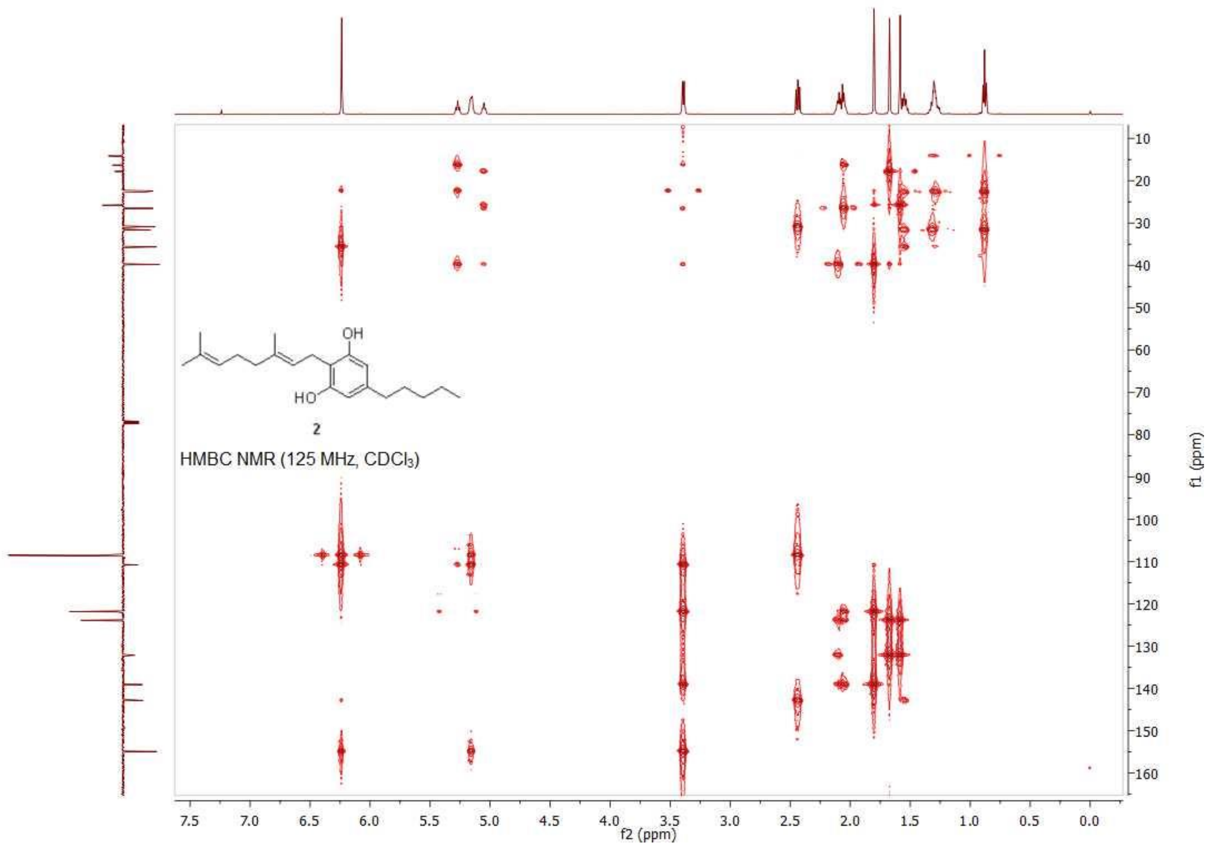
OH
HO
2
HMBC NMR (125 MHz, CDCl3)
f2 (ppm)
f1 (ppm)

NMR spectra of compound 16a

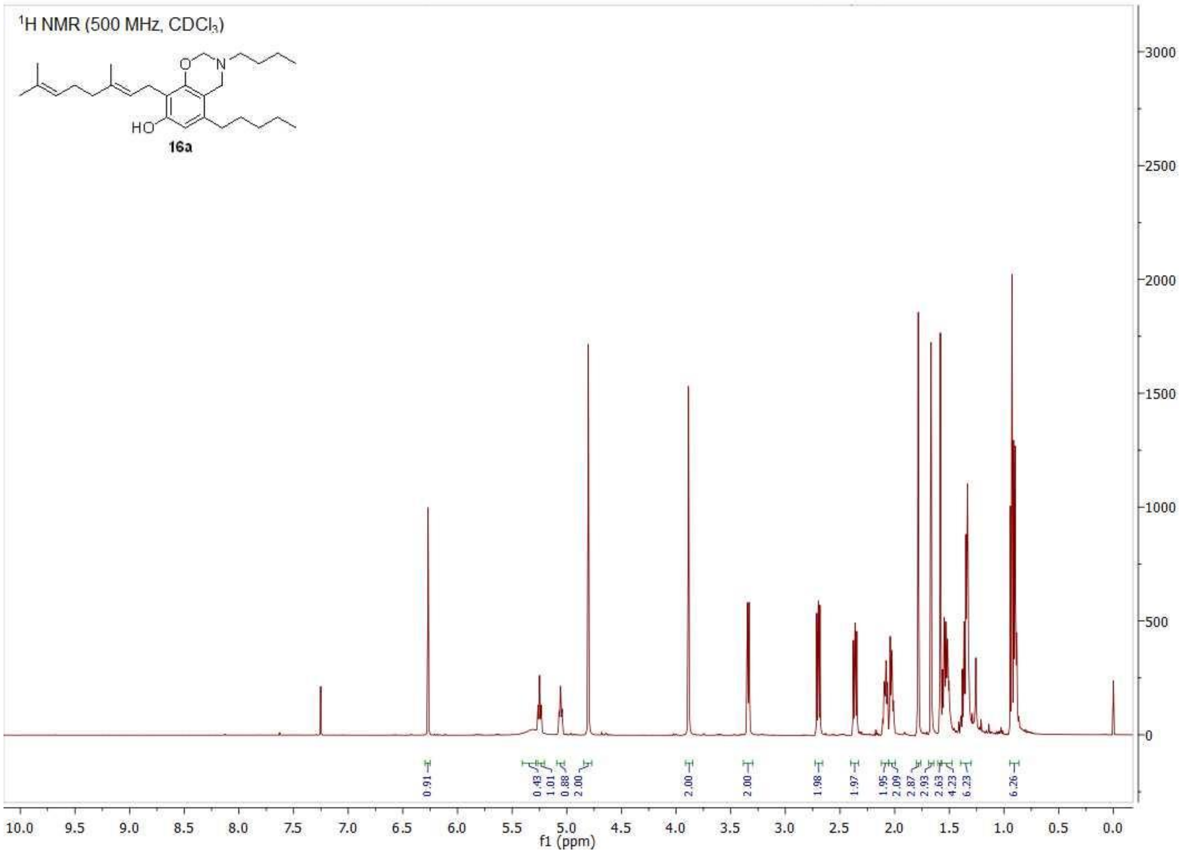

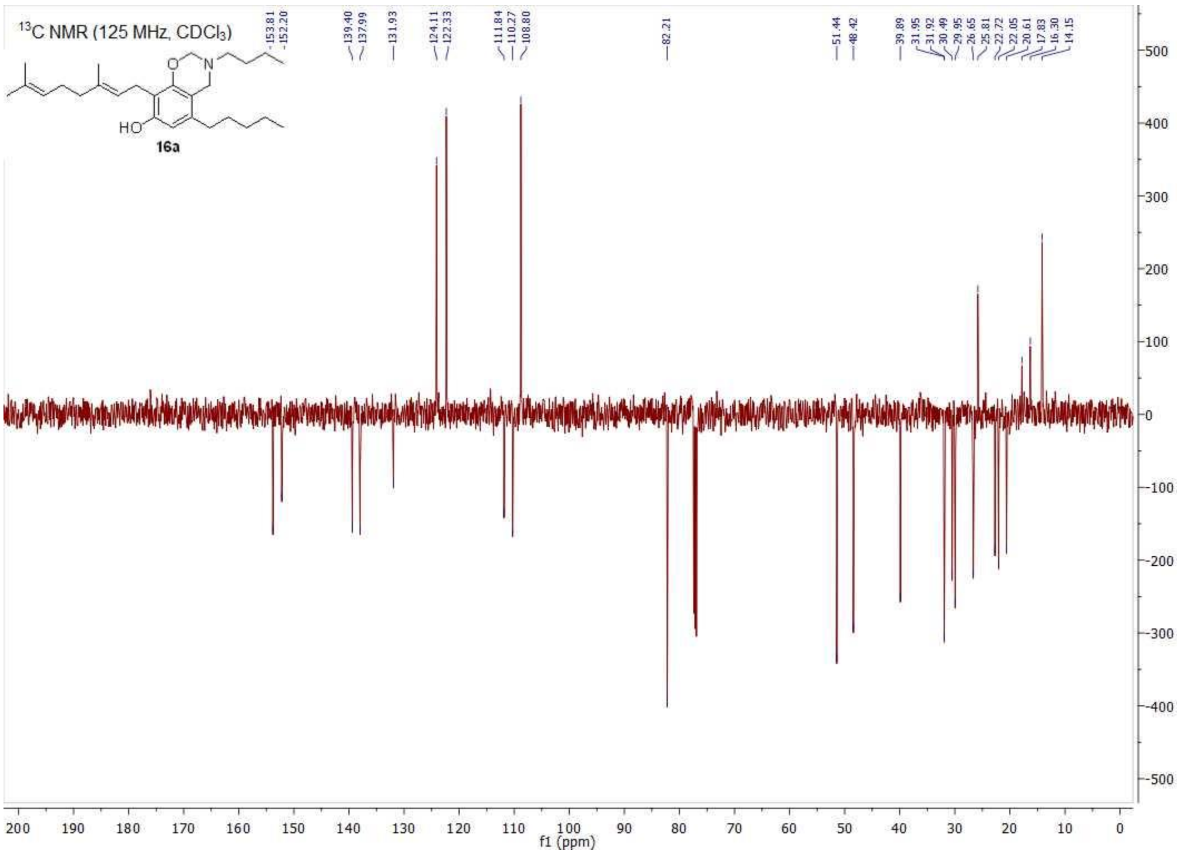

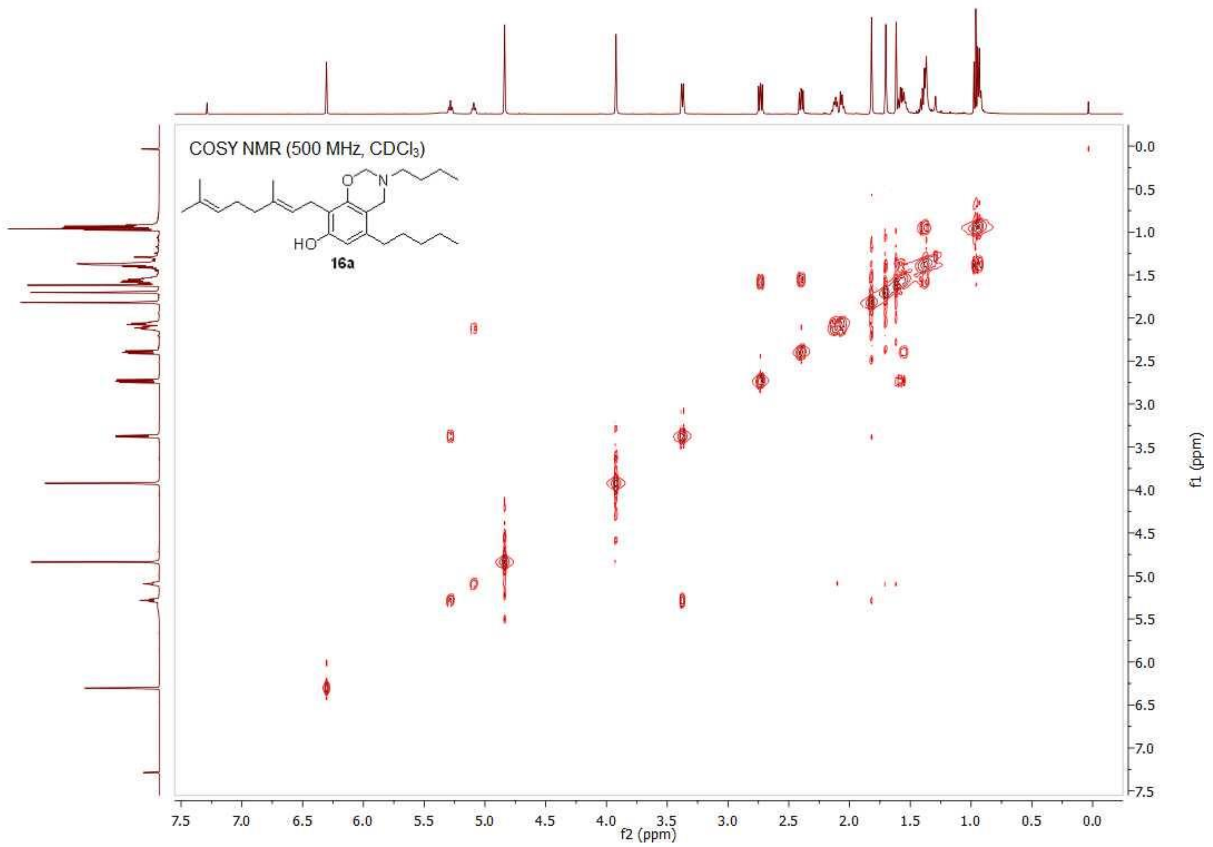
COSY NMR (500 MHz, CDCl3)
O
N
HO
16a
f2 (ppm)
f1 (ppm)

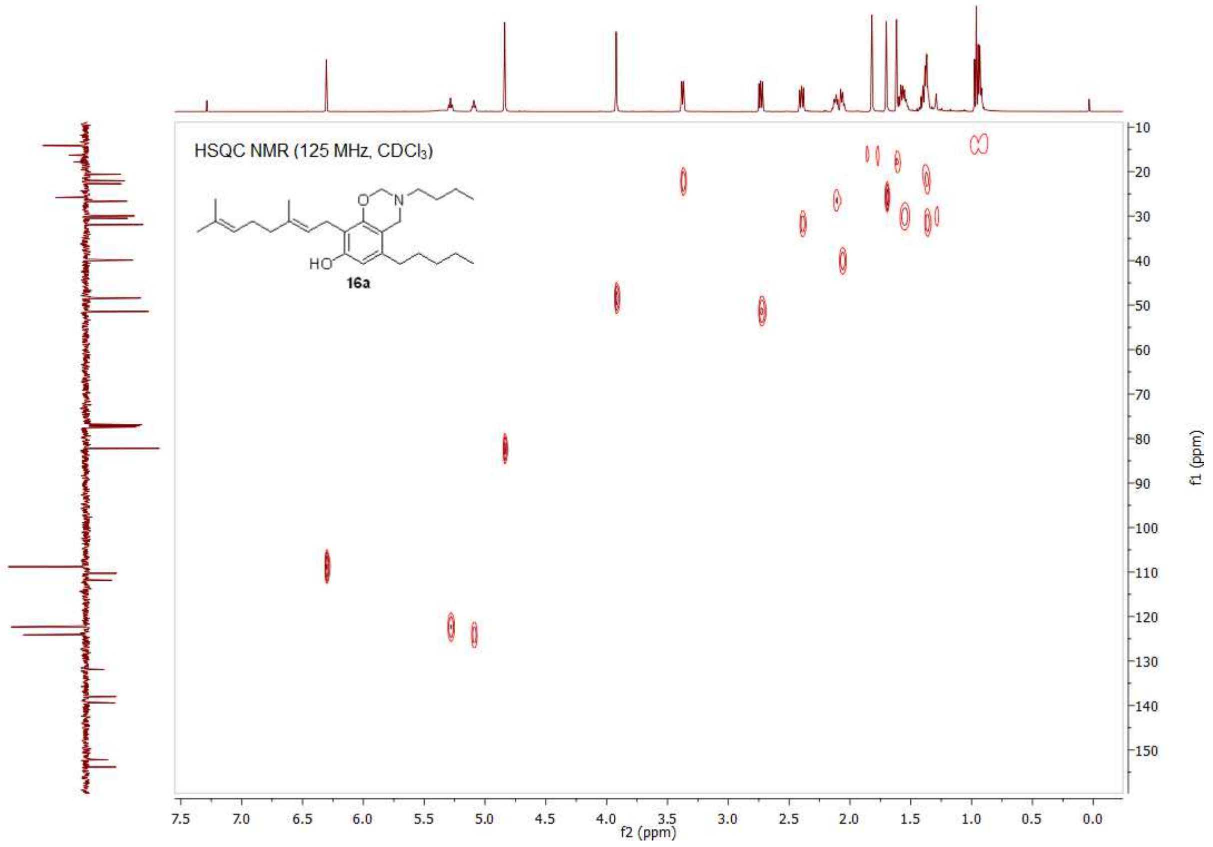
HSQC NMR (125 MHz, CDCl3)
O
N
HO
16a
f2 (ppm)
f1 (ppm)

NMR spectra of compound 16b

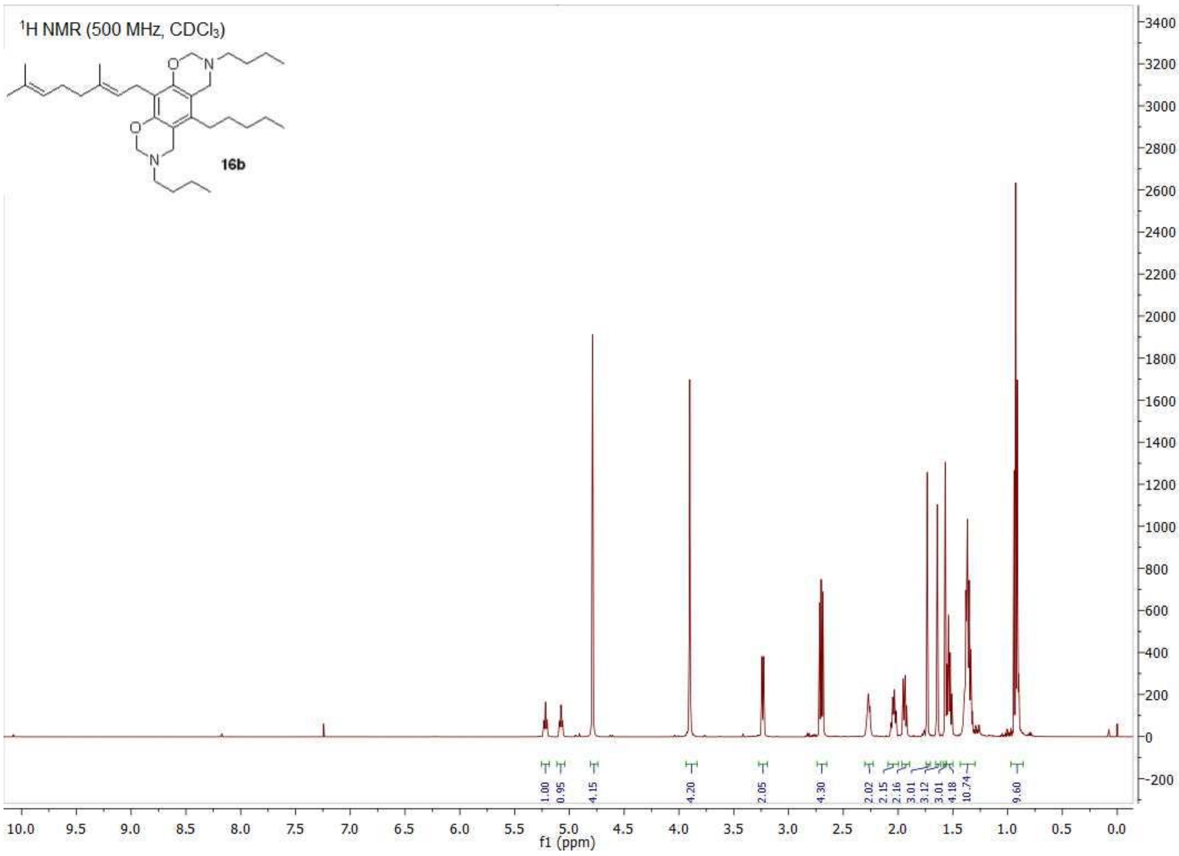

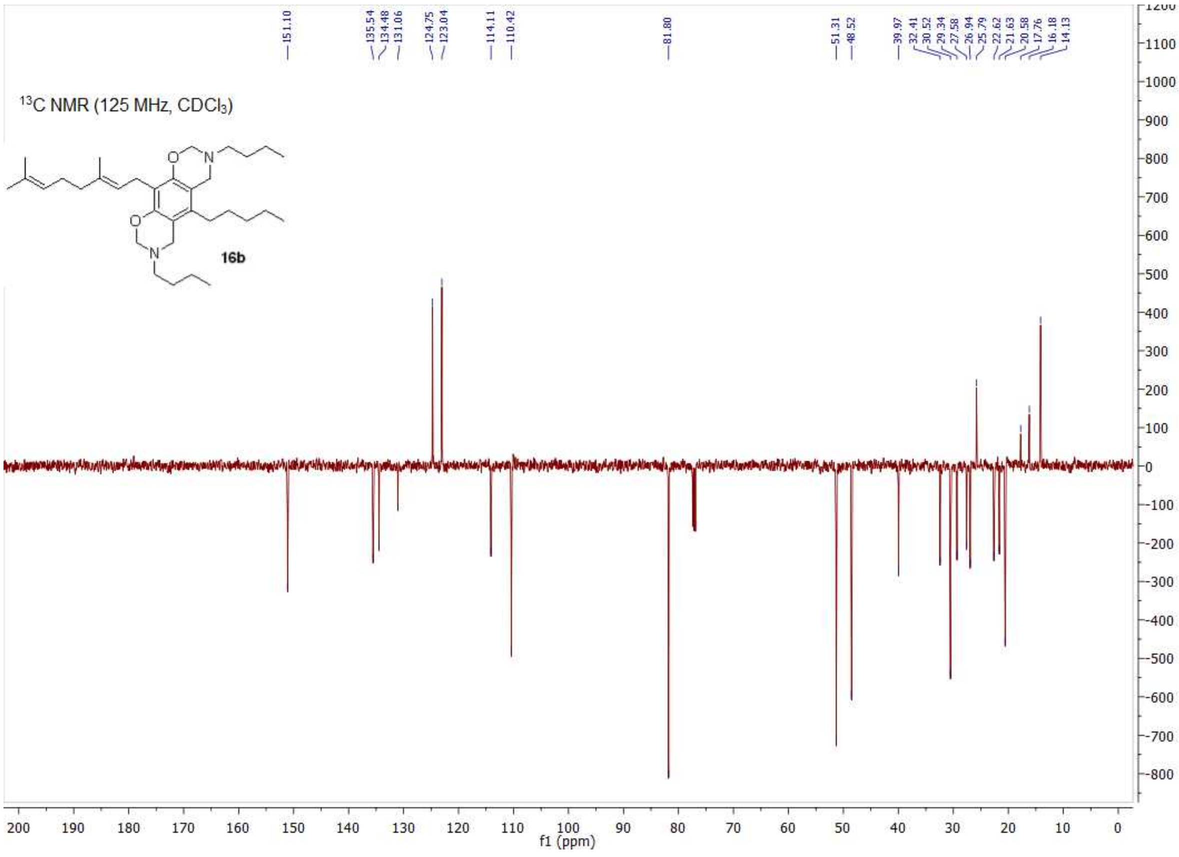

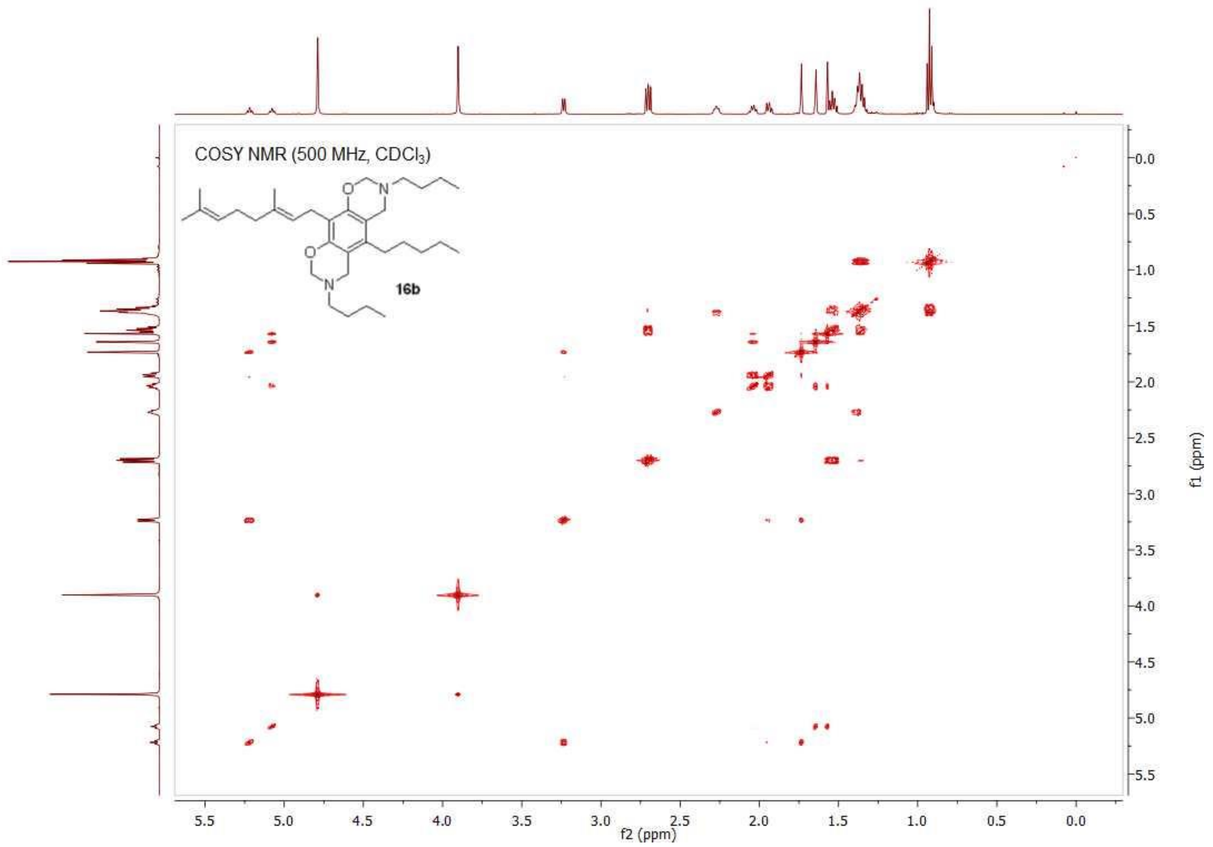
COSY NMR (500 MHz, CDCl3)
16b
f2 (ppm)
f1 (ppm)

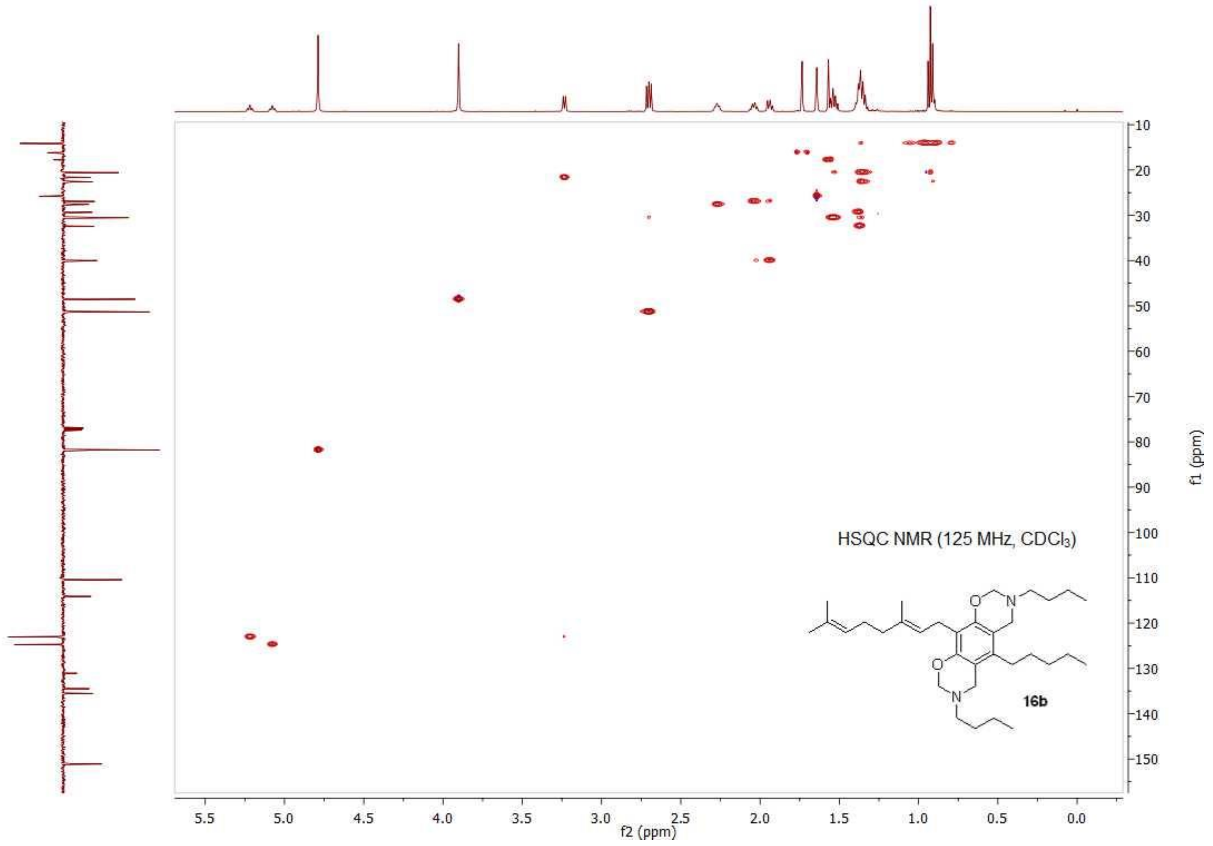
HSQC NMR (125 MHz, CDCl3)
16b
f2 (ppm)
f1 (ppm)

NMR spectra of compound 17a

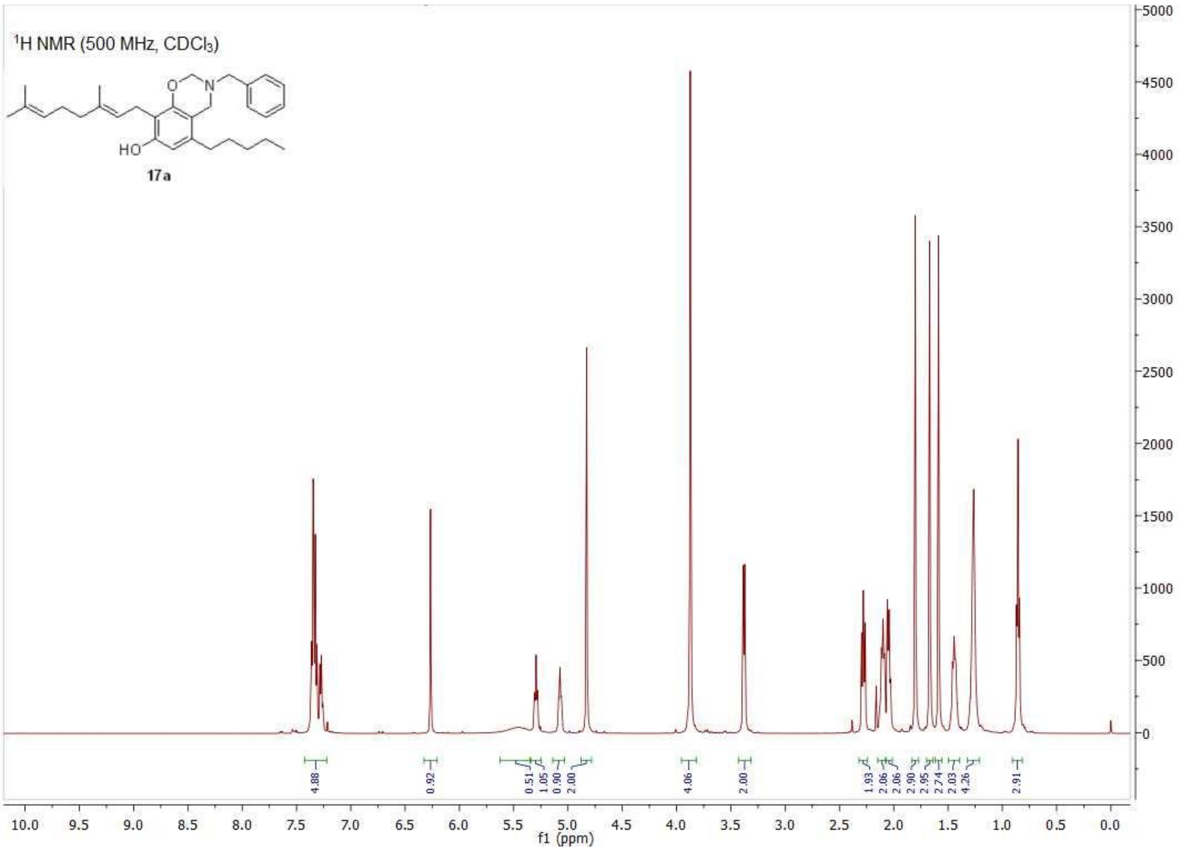

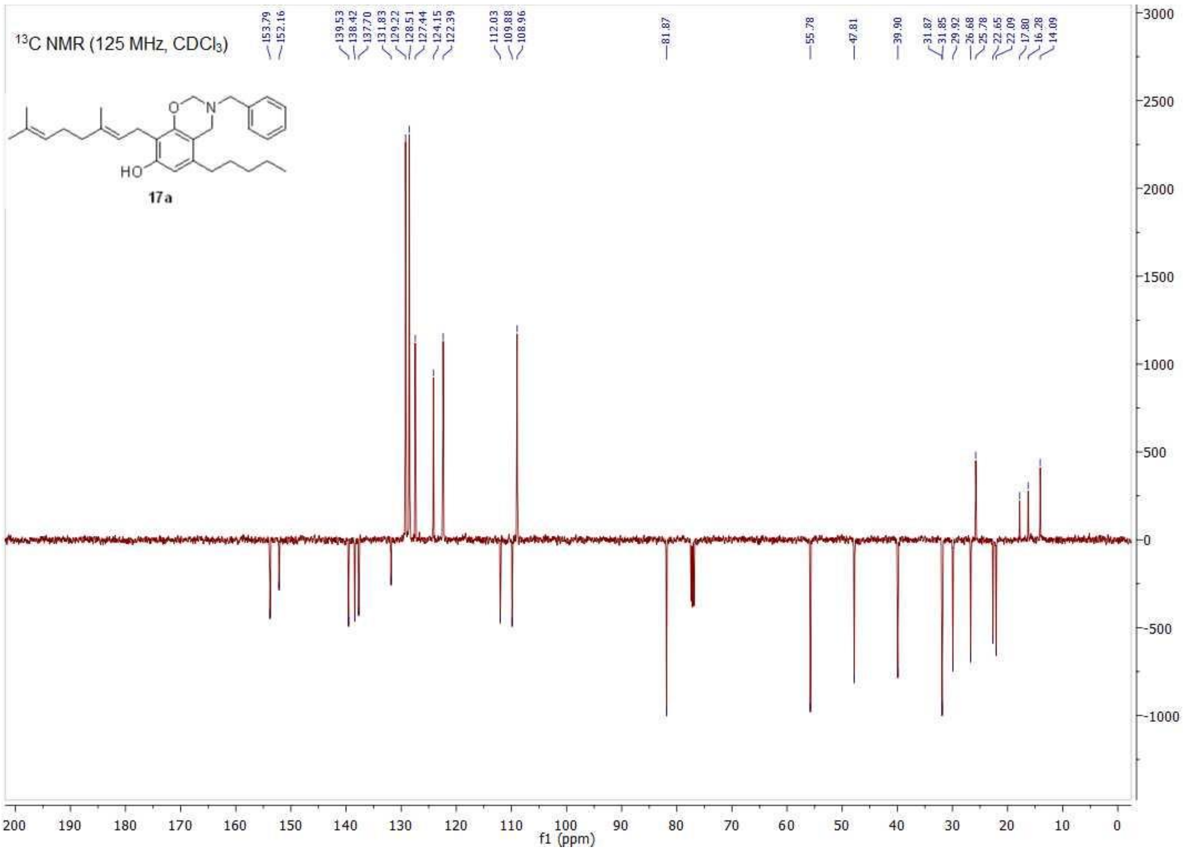

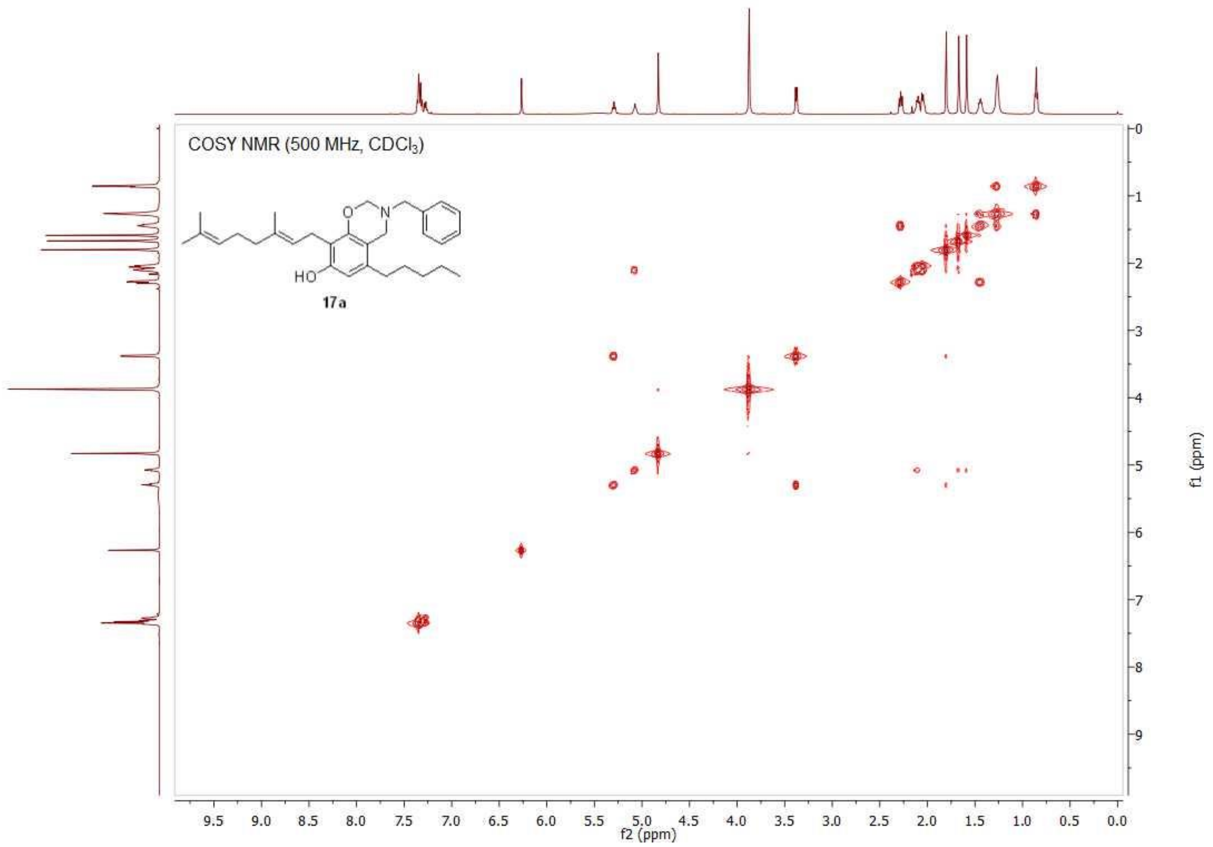
COSY NMR (500 MHz, CDCl3)
HO
17a
f2 (ppm)
f1 (ppm)

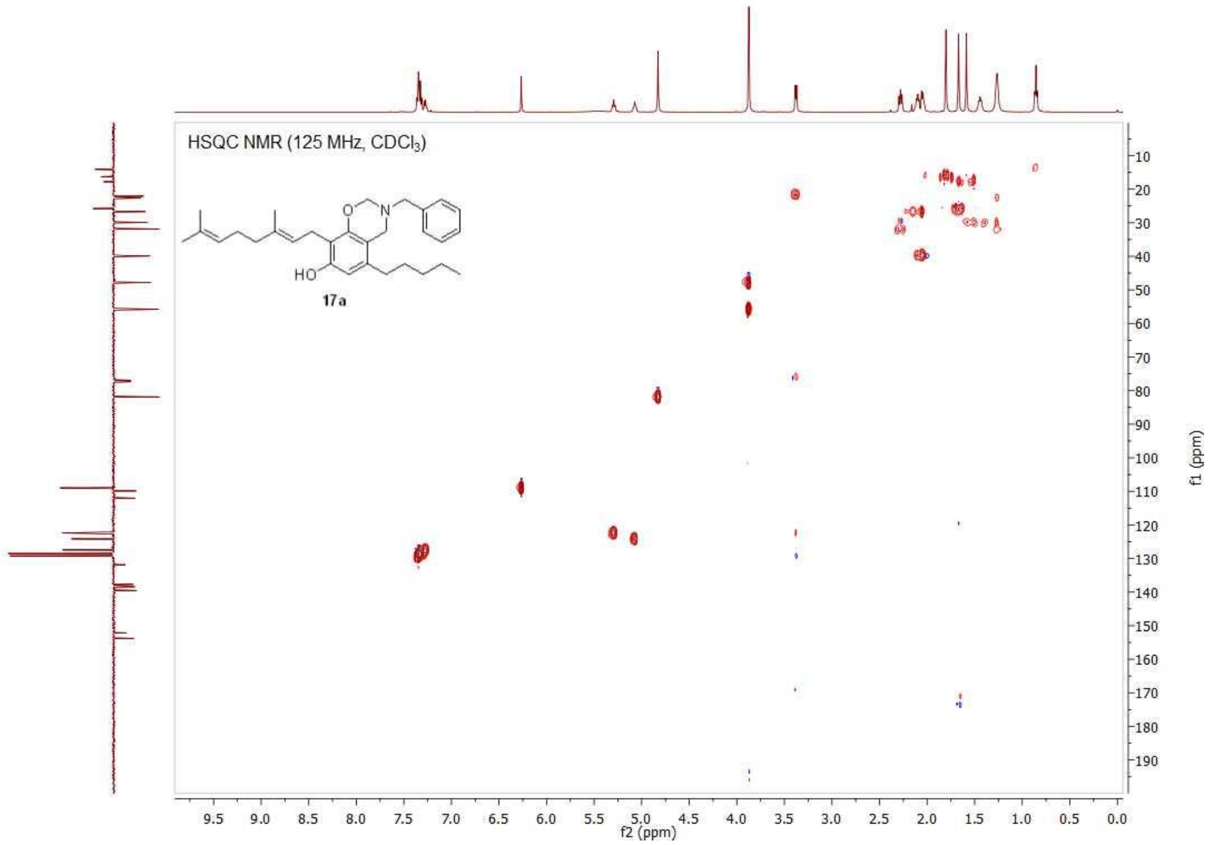
HSQC NMR (125 MHz, CDCl3)
HO
17a
f2 (ppm)
f1 (ppm)

NMR spectra of compound 17b

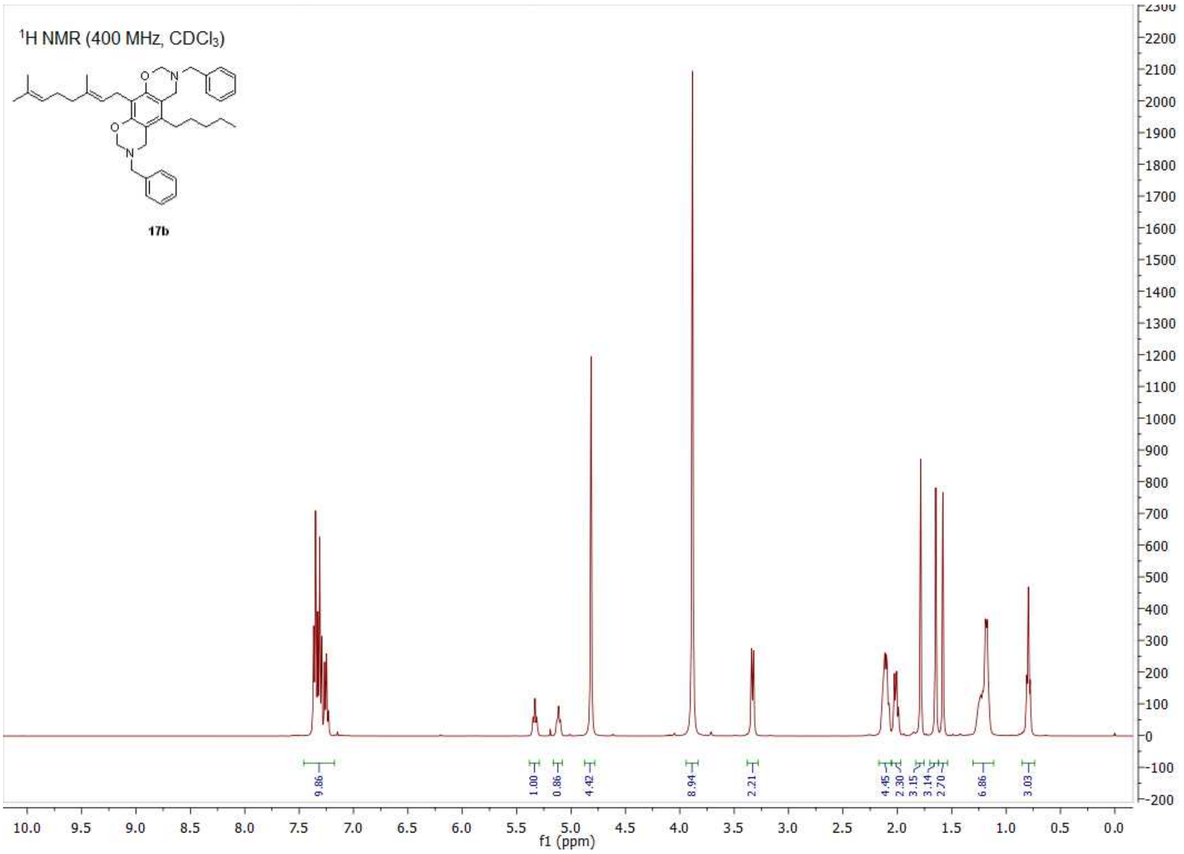

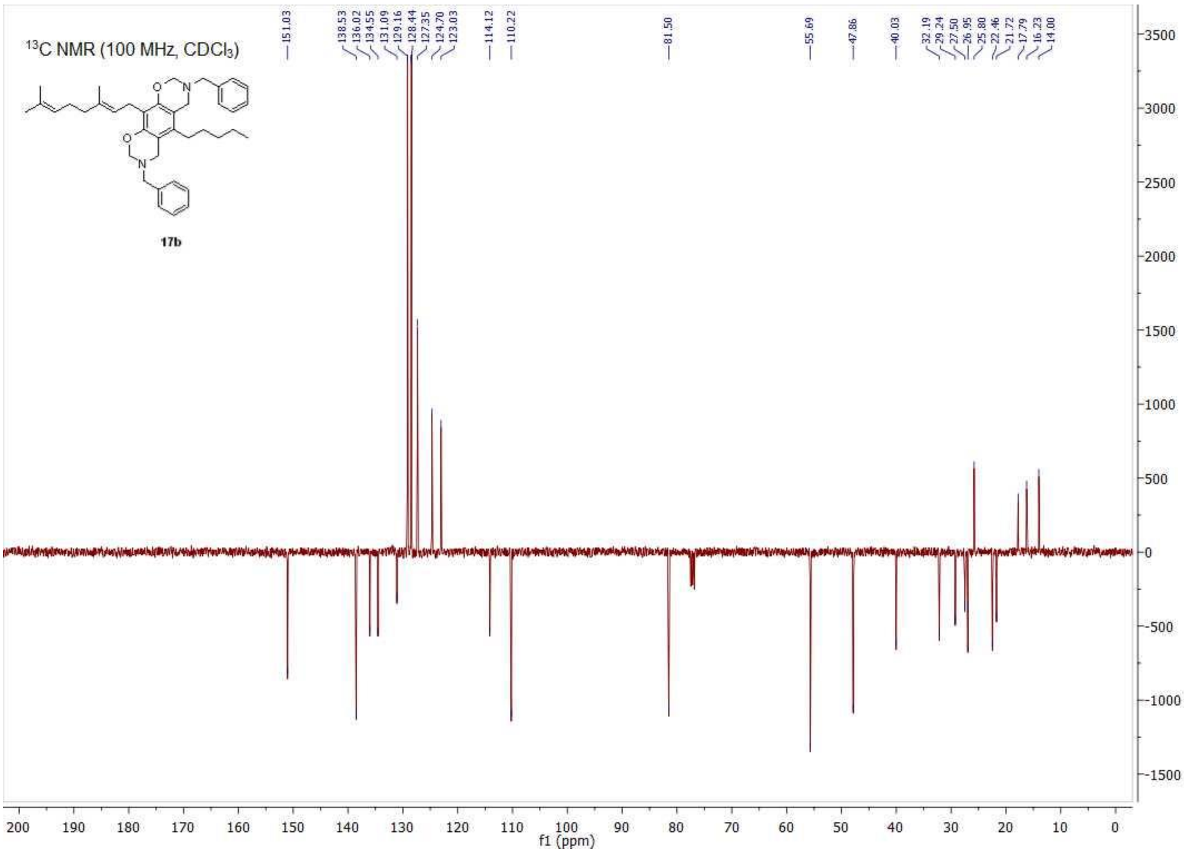

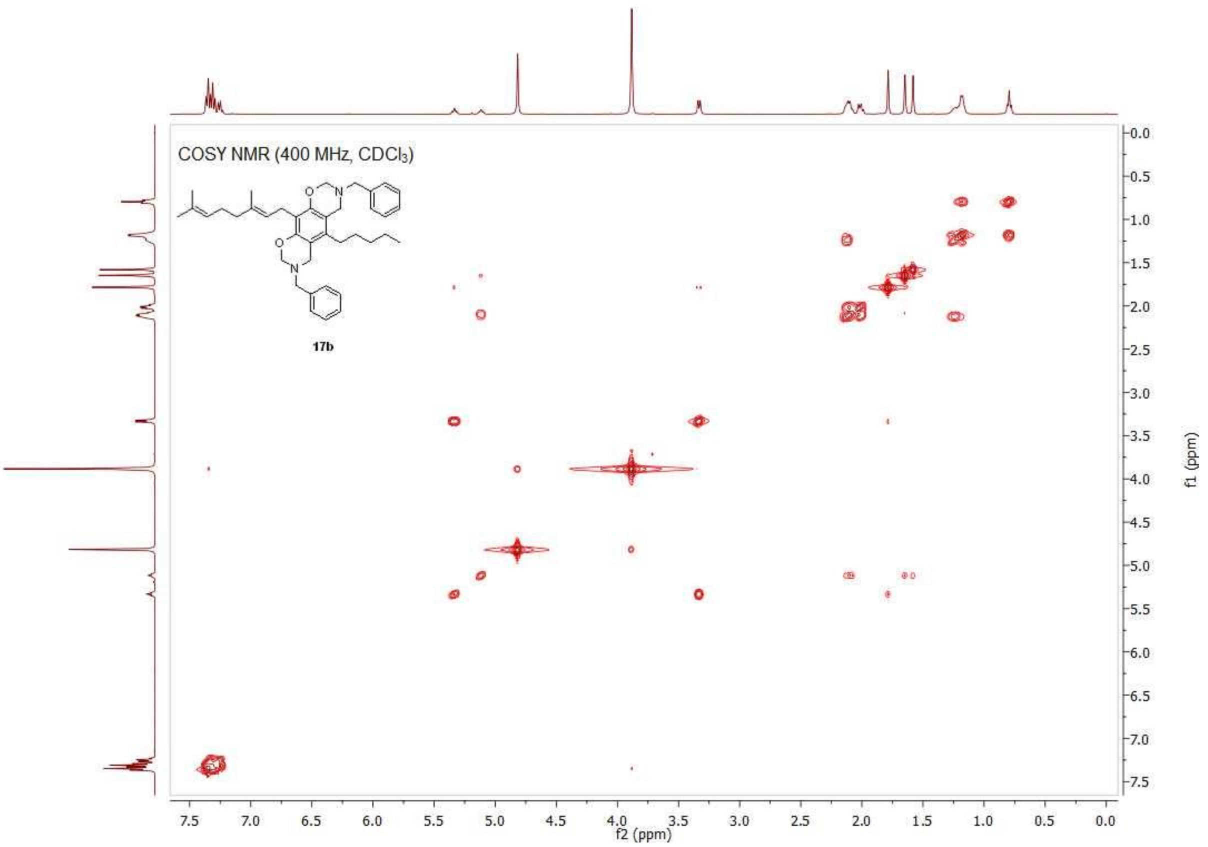

COSY NMR (400 MHz, CDCl3)
17b
f2 (ppm)
f1 (ppm)

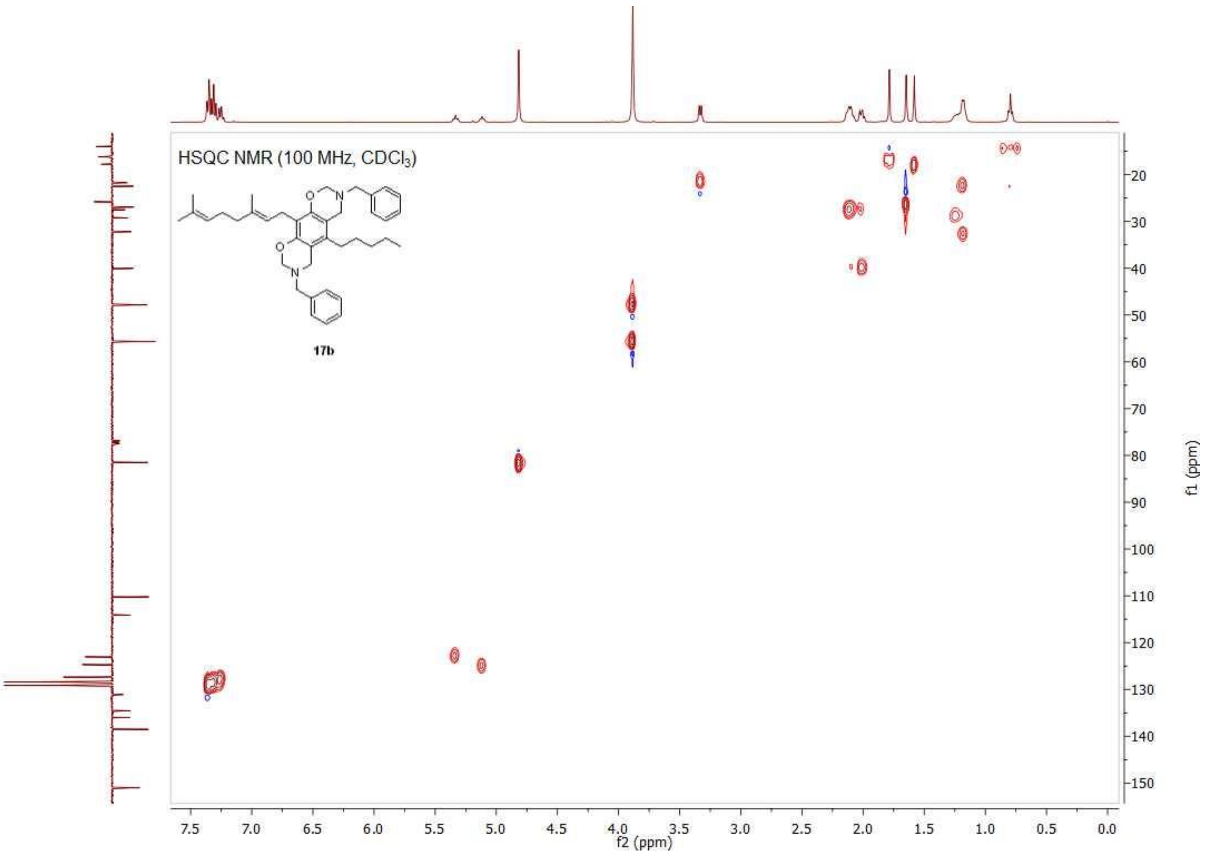

HSQC NMR (100 MHz, CDCl3)
17b
f2 (ppm)
f1 (ppm)

NMR spectra of compound 18

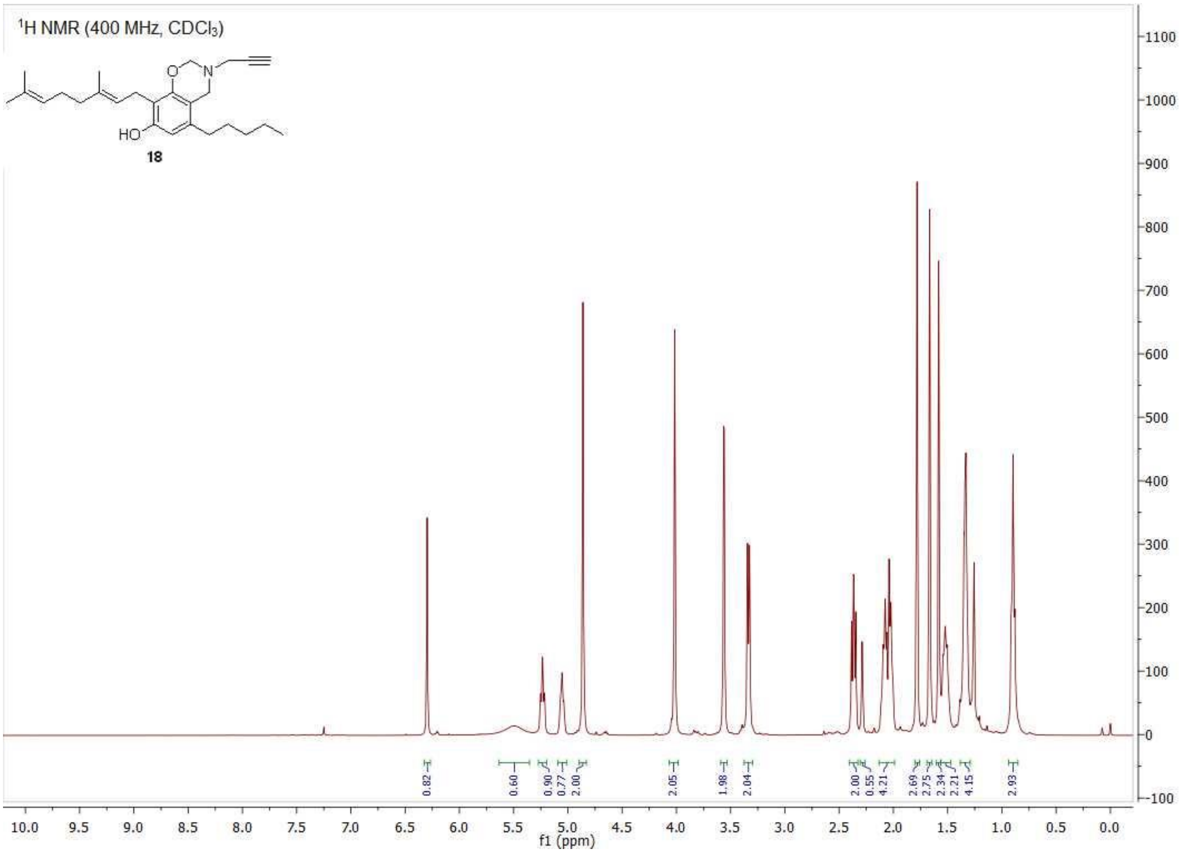

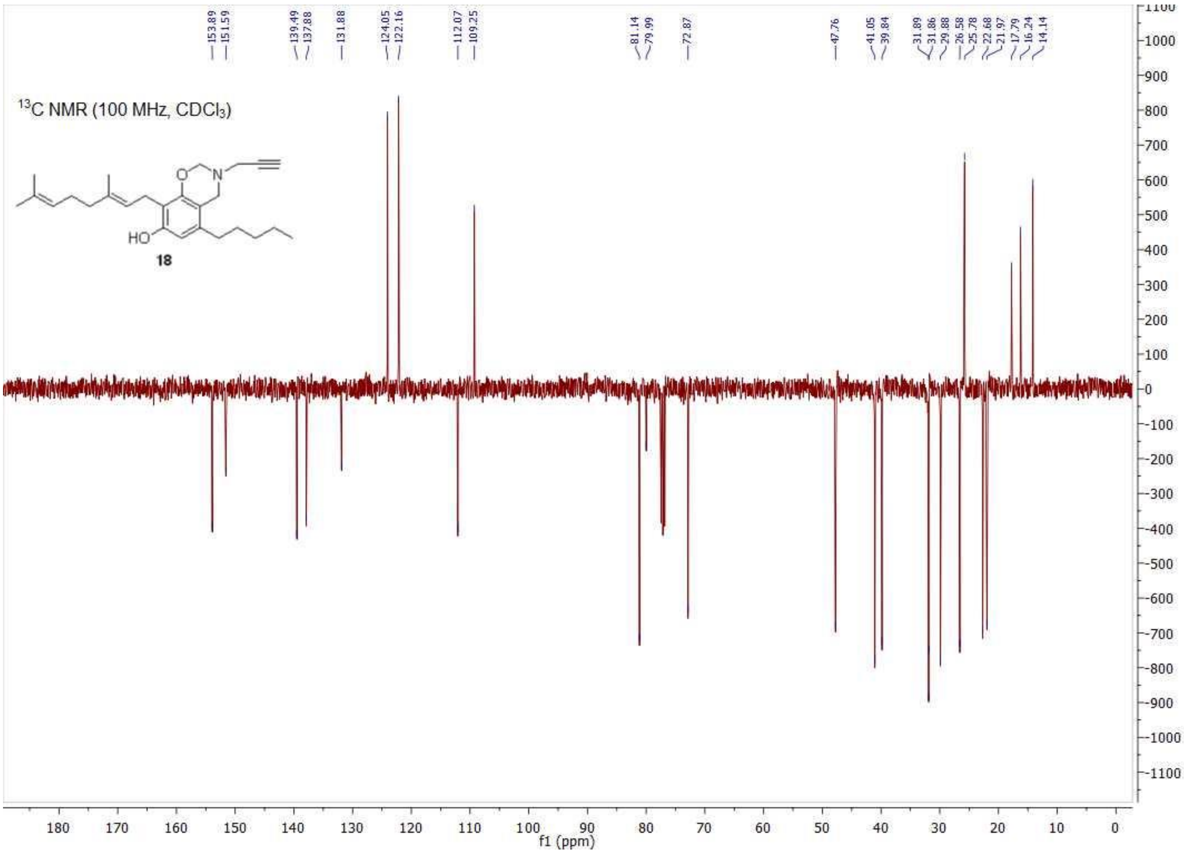

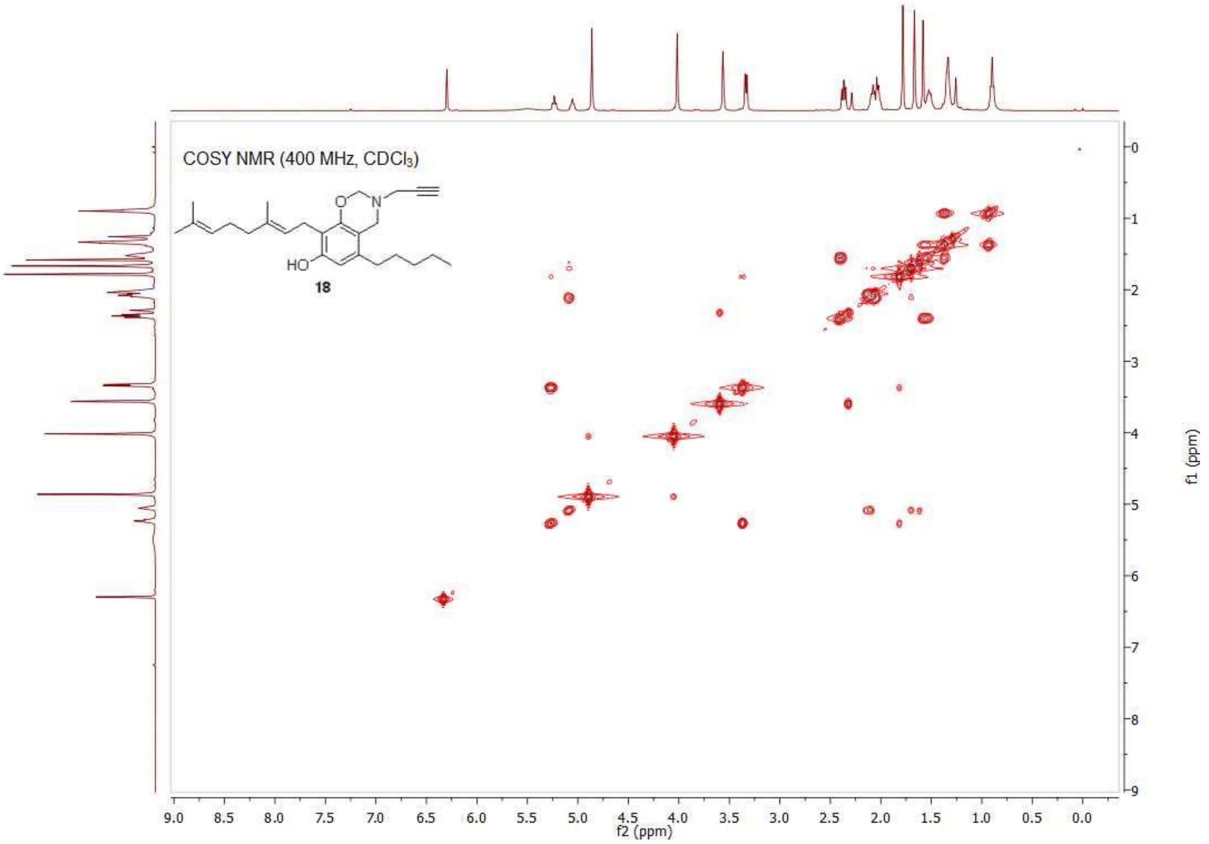
COSY NMR (400 MHz, CDCl3)
HO
18
f2 (ppm)
f1 (ppm)

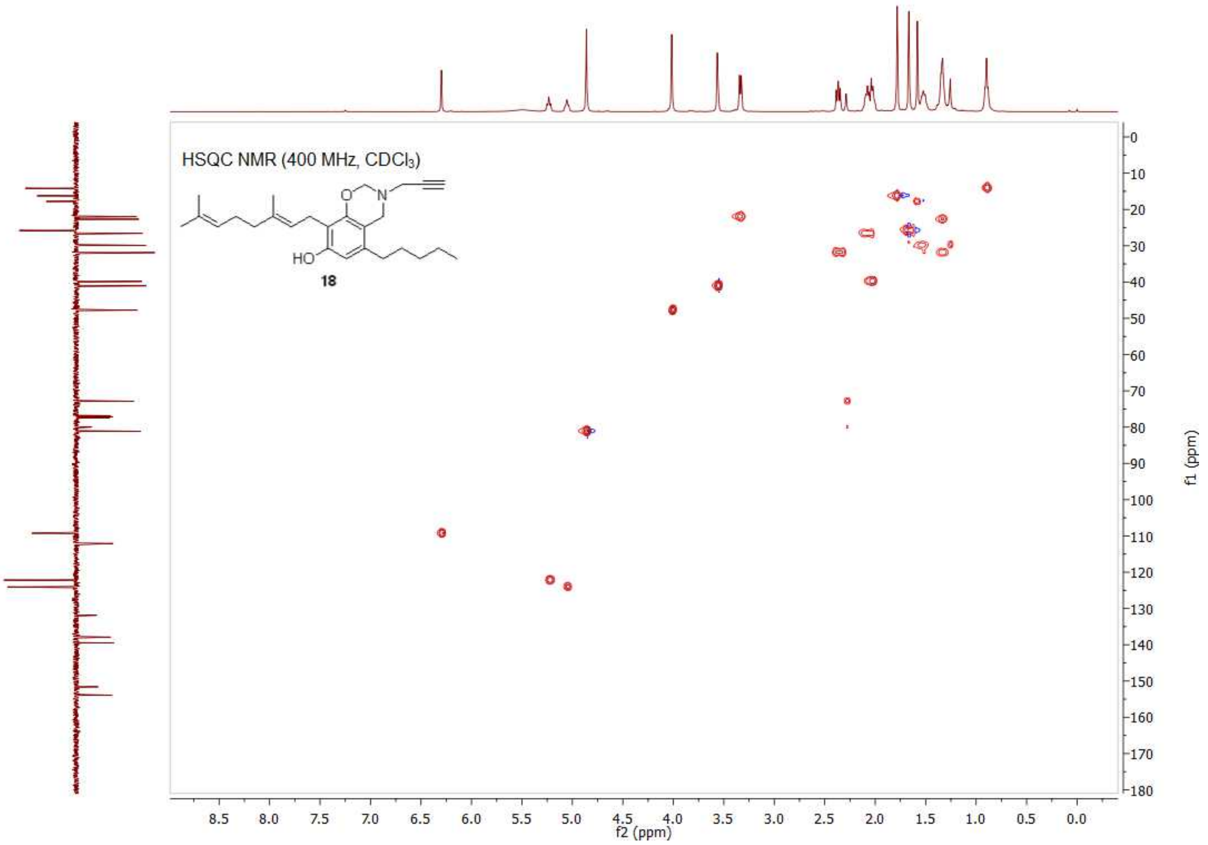
HSQC NMR (400 MHz, CDCl3)
HO
18
f2 (ppm)
f1 (ppm)

NMR spectra of compound 19a

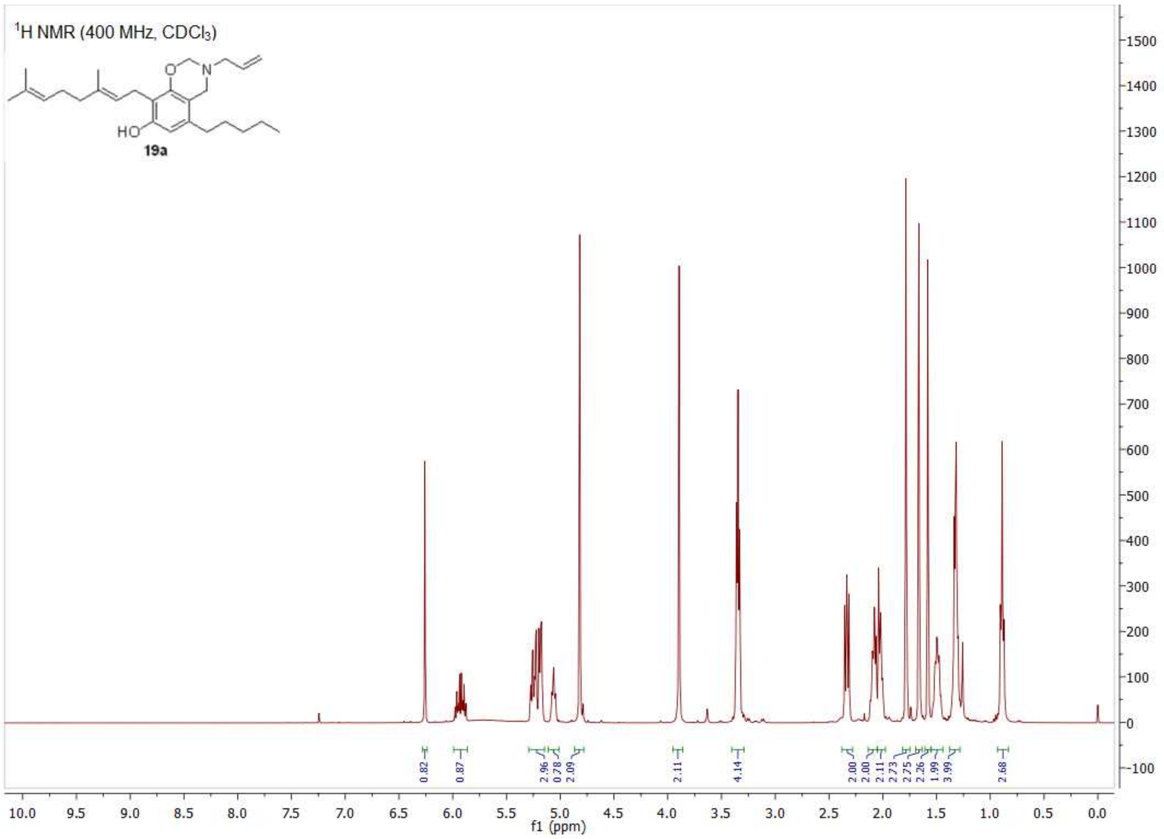

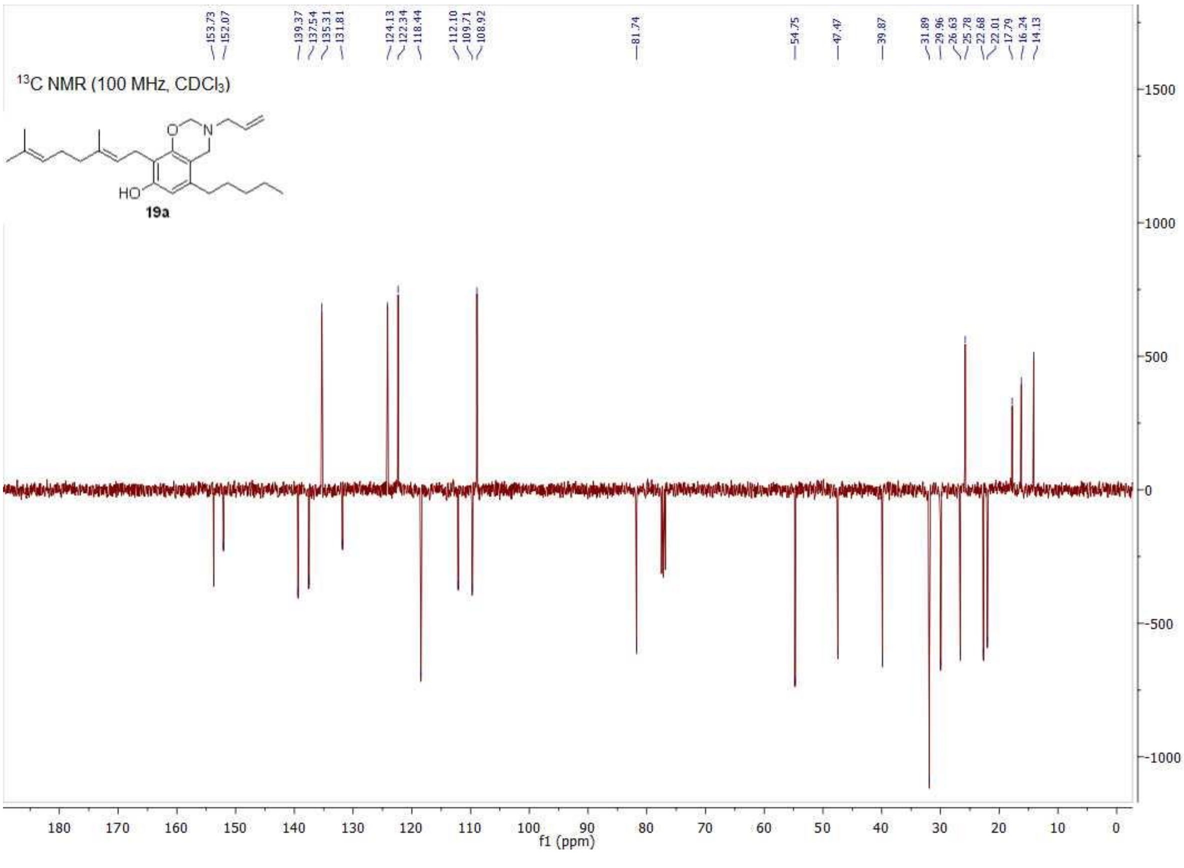

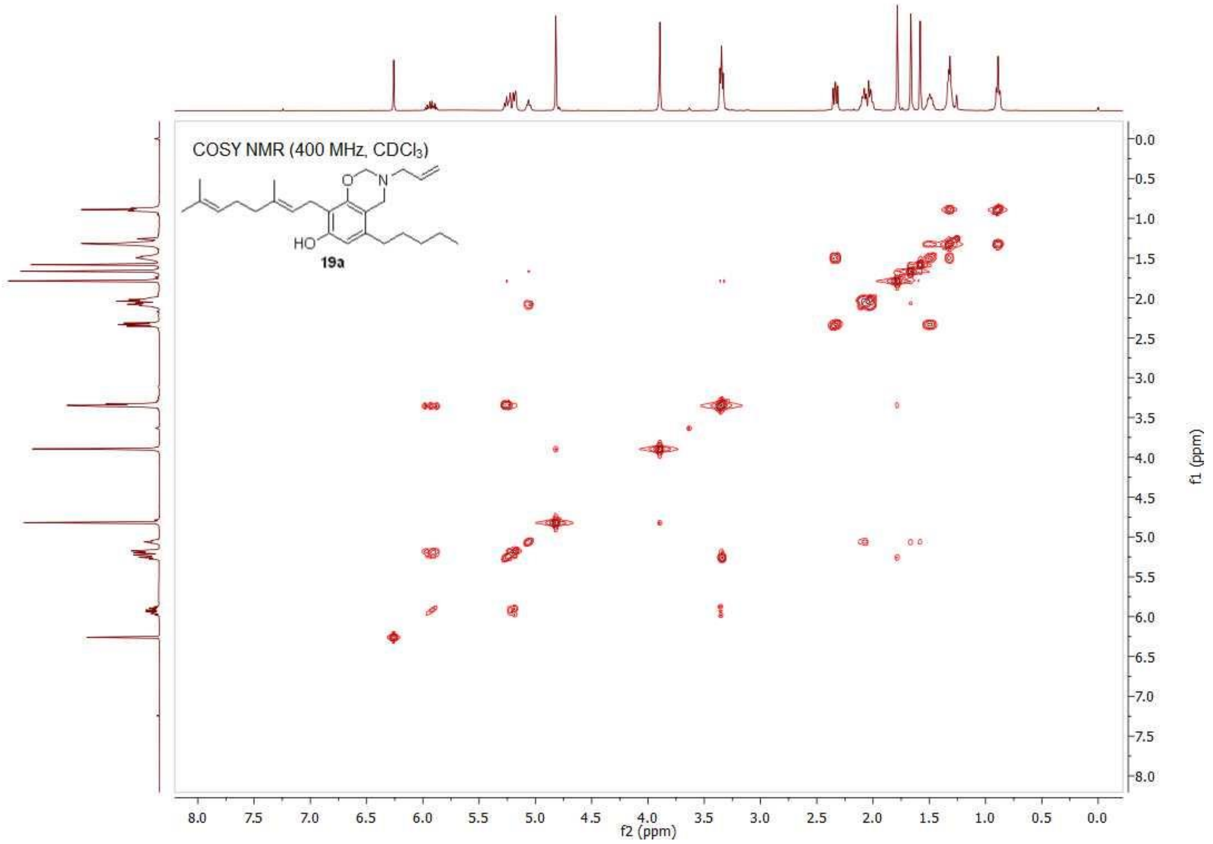
COSY NMR (400 MHz, CDCl3)
HO
19a
f2 (ppm)
f1 (ppm)

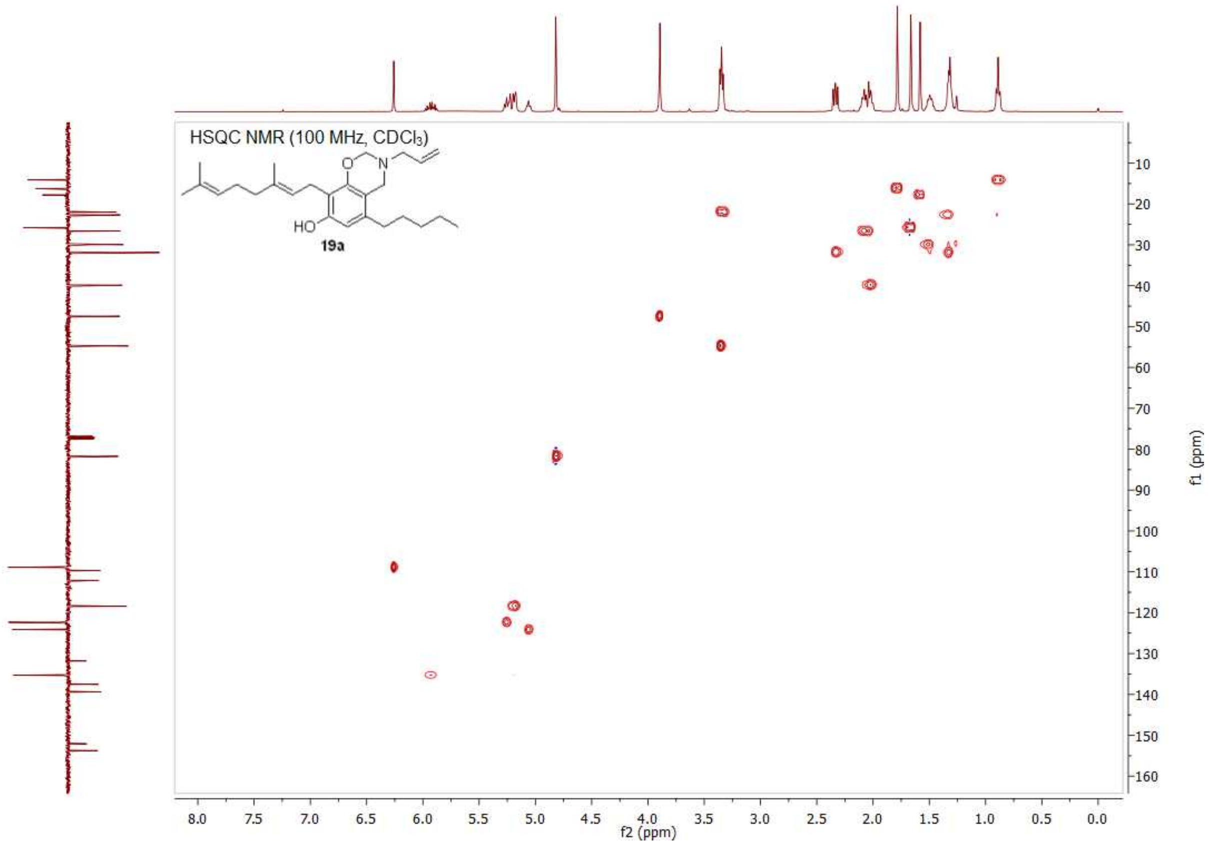
HSQC NMR (100 MHz, CDCl3)
HO
19a
f2 (ppm)
f1 (ppm)

NMR spectra of compound 19b

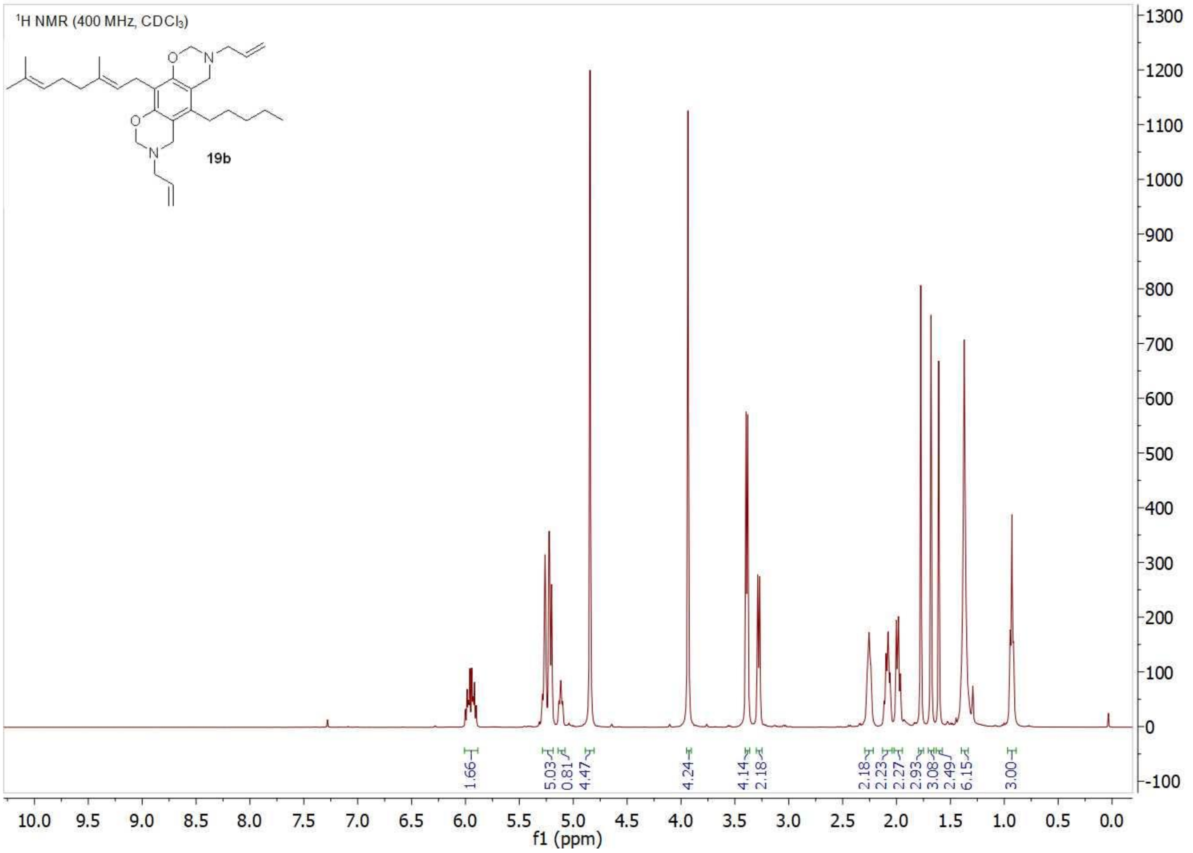

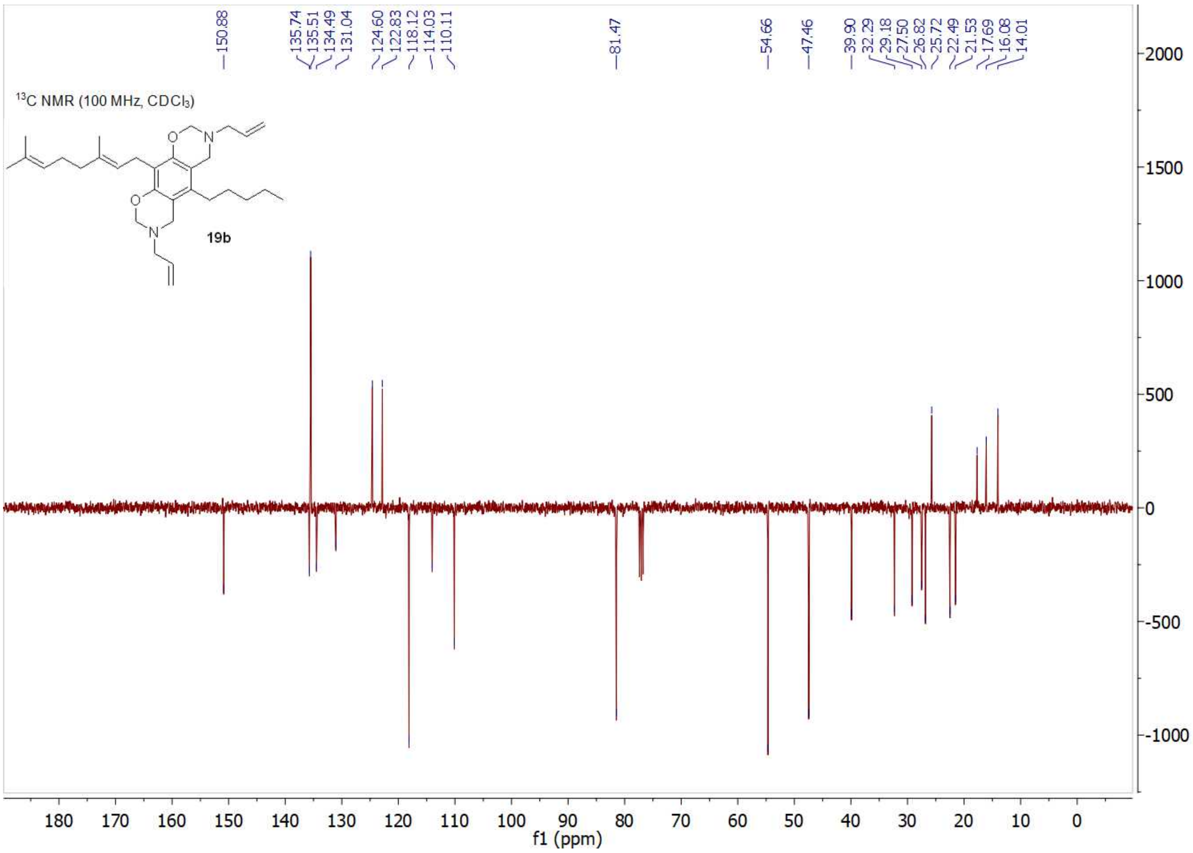

NMR spectra of compound 20

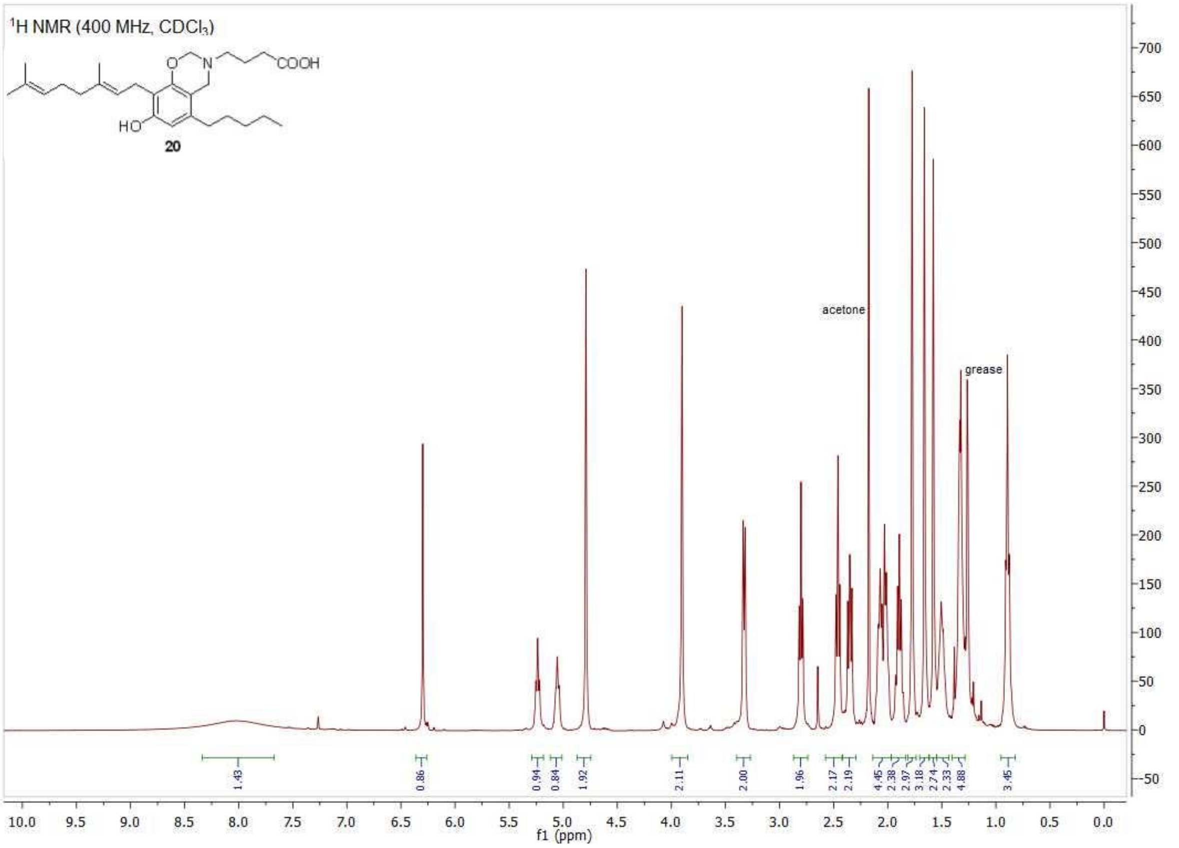

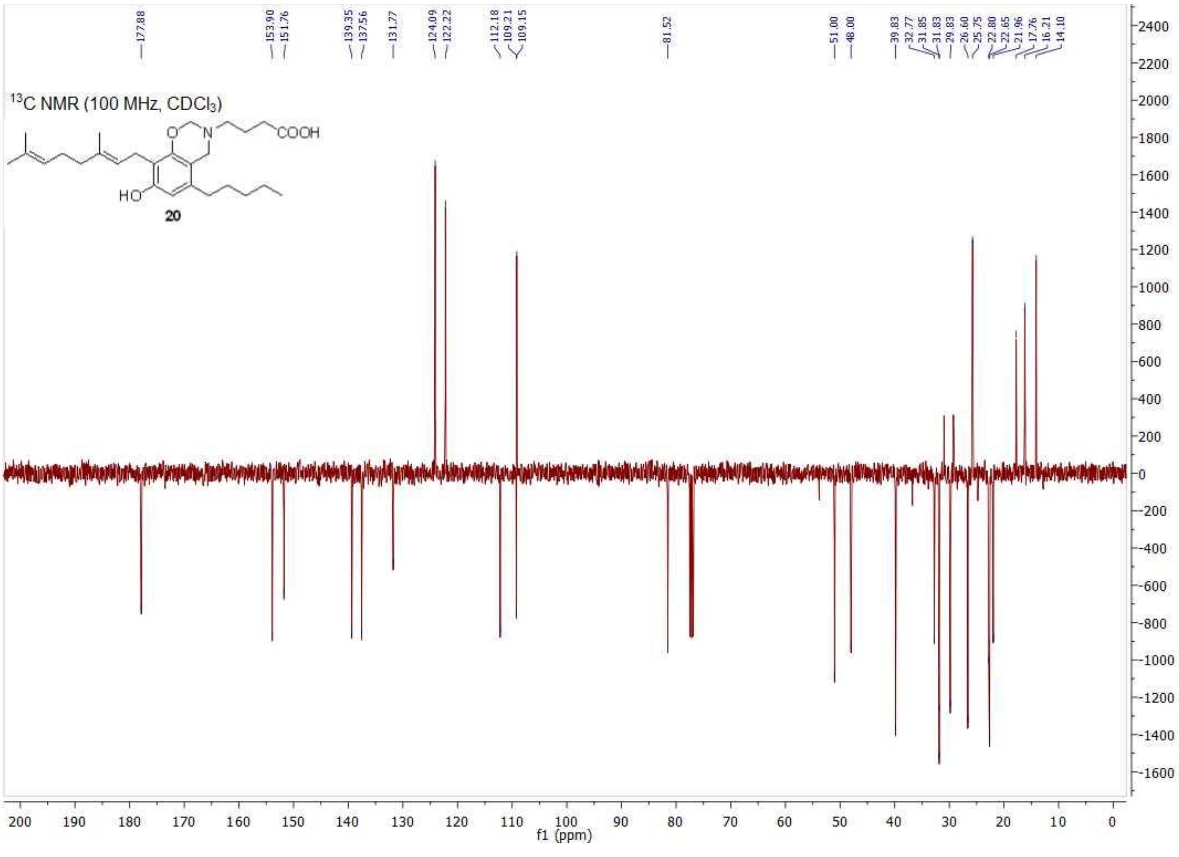

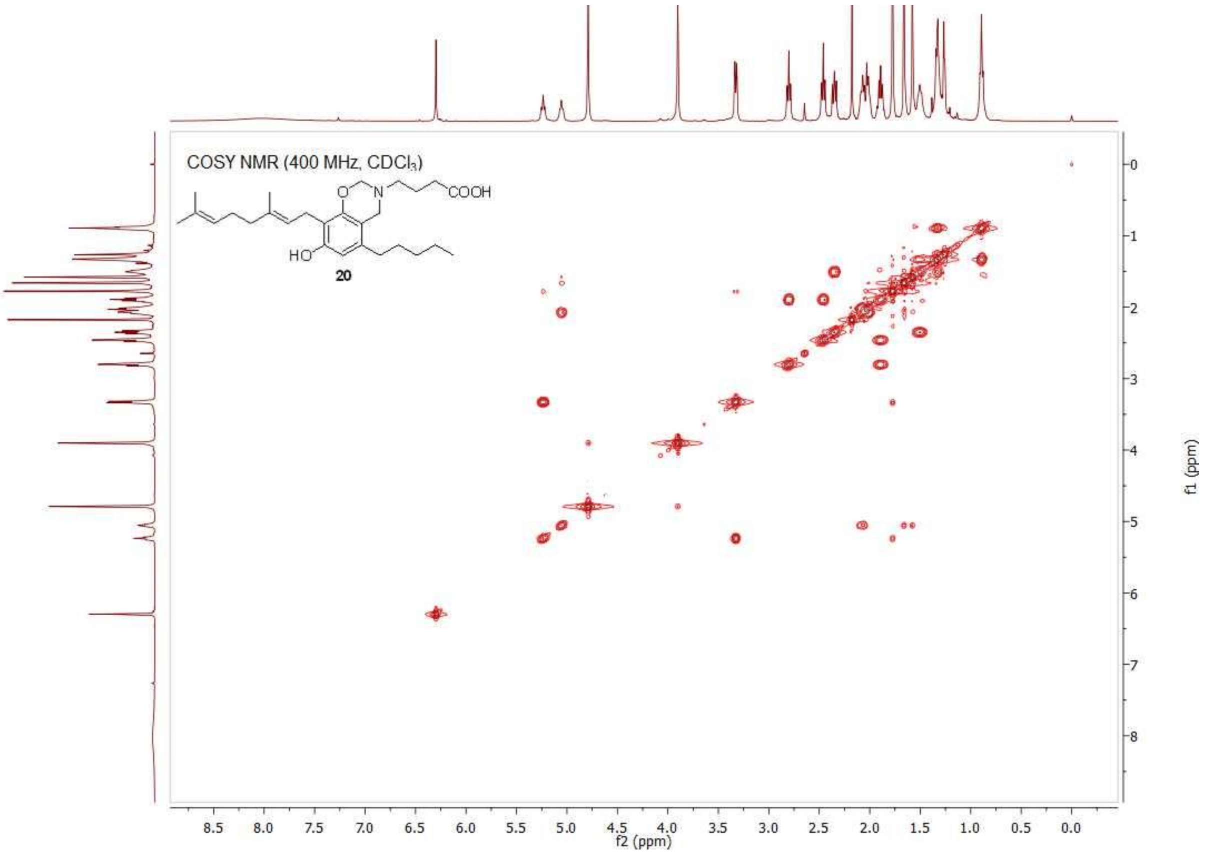
COSY NMR (400 MHz, CDCl3)
COOH
HO
20
f2 (ppm)
f1 (ppm)

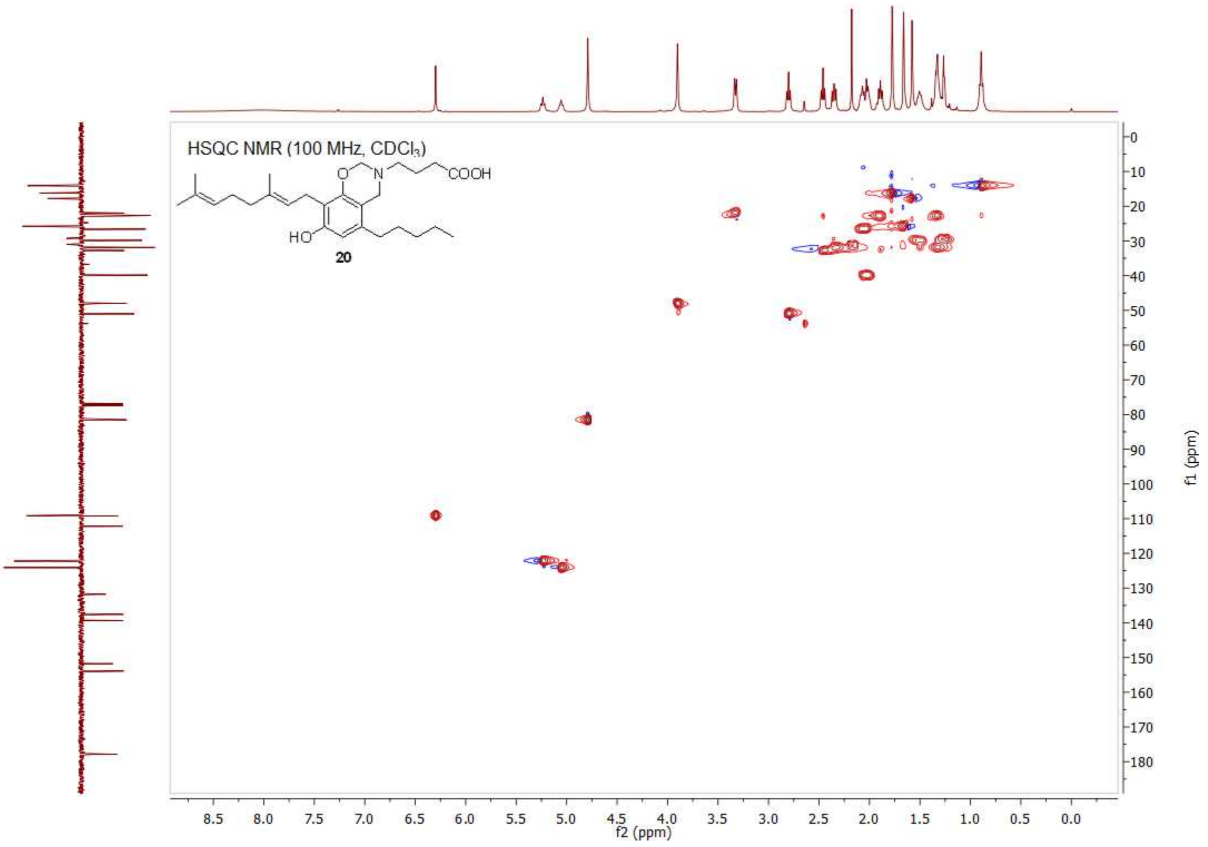
HSQC NMR (100 MHz, CDCl3)
COOH
HO
20
f2 (ppm)
f1 (ppm)

NMR spectra of compound 21a

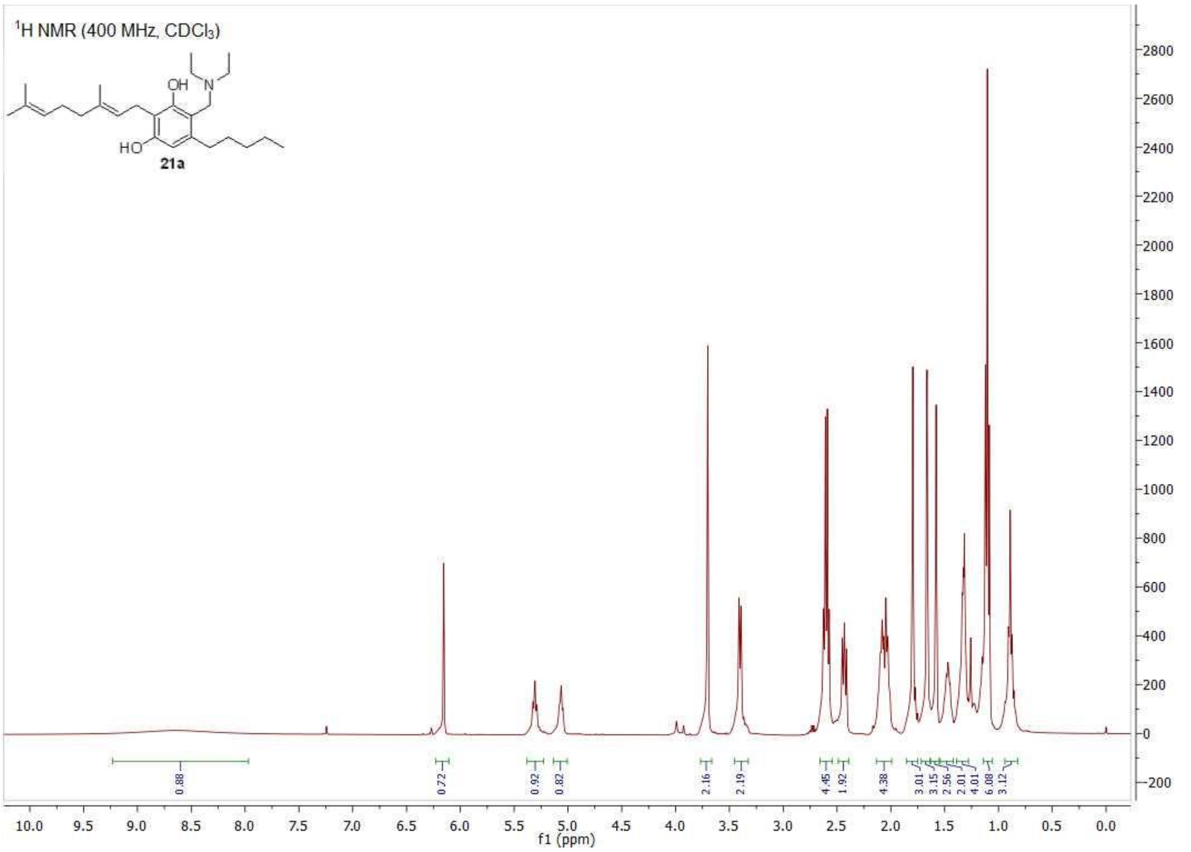

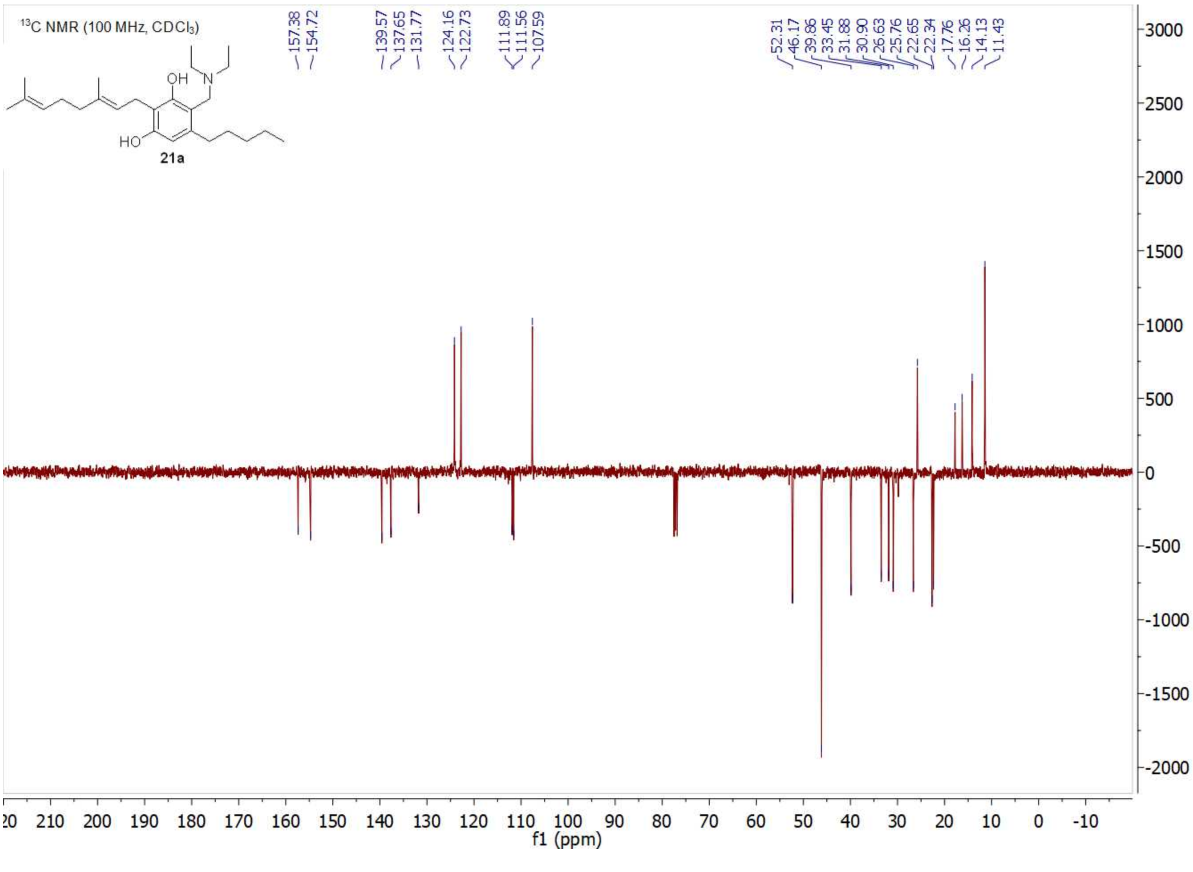

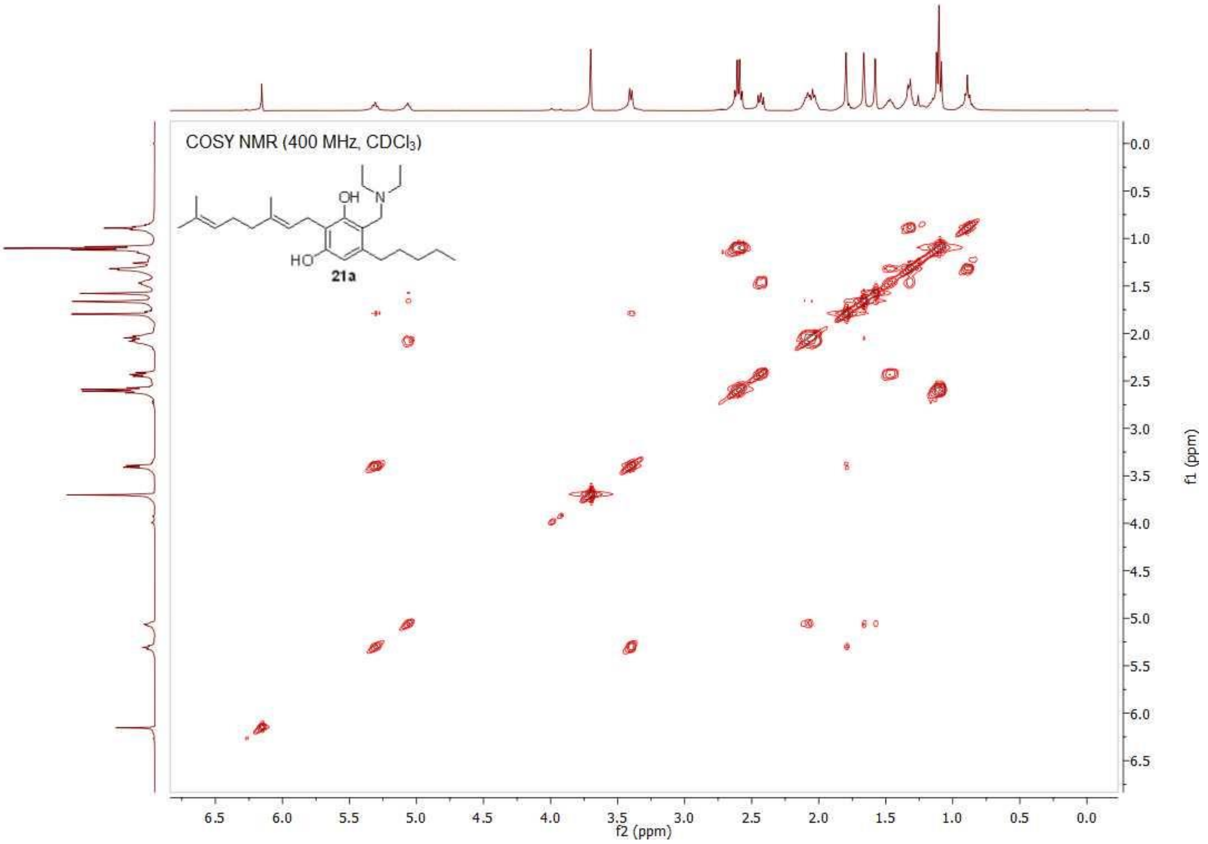
COSY NMR (400 MHz, CDCl3)
OH
N
HO
21a
f2 (ppm)
f1 (ppm)

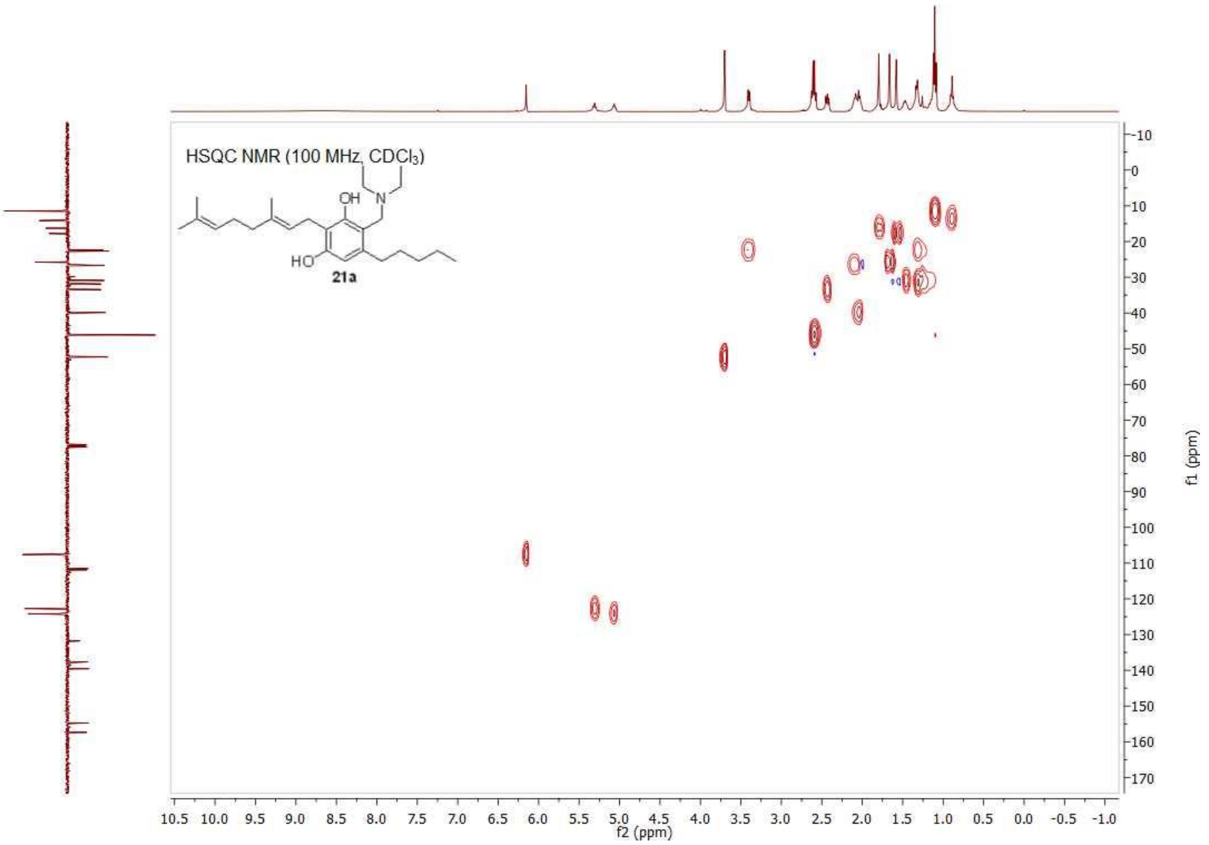
HSQC NMR (100 MHz, CDCl3)
OH
N
HO
21a
f2 (ppm)
f1 (ppm)

NMR spectra of compound 21b

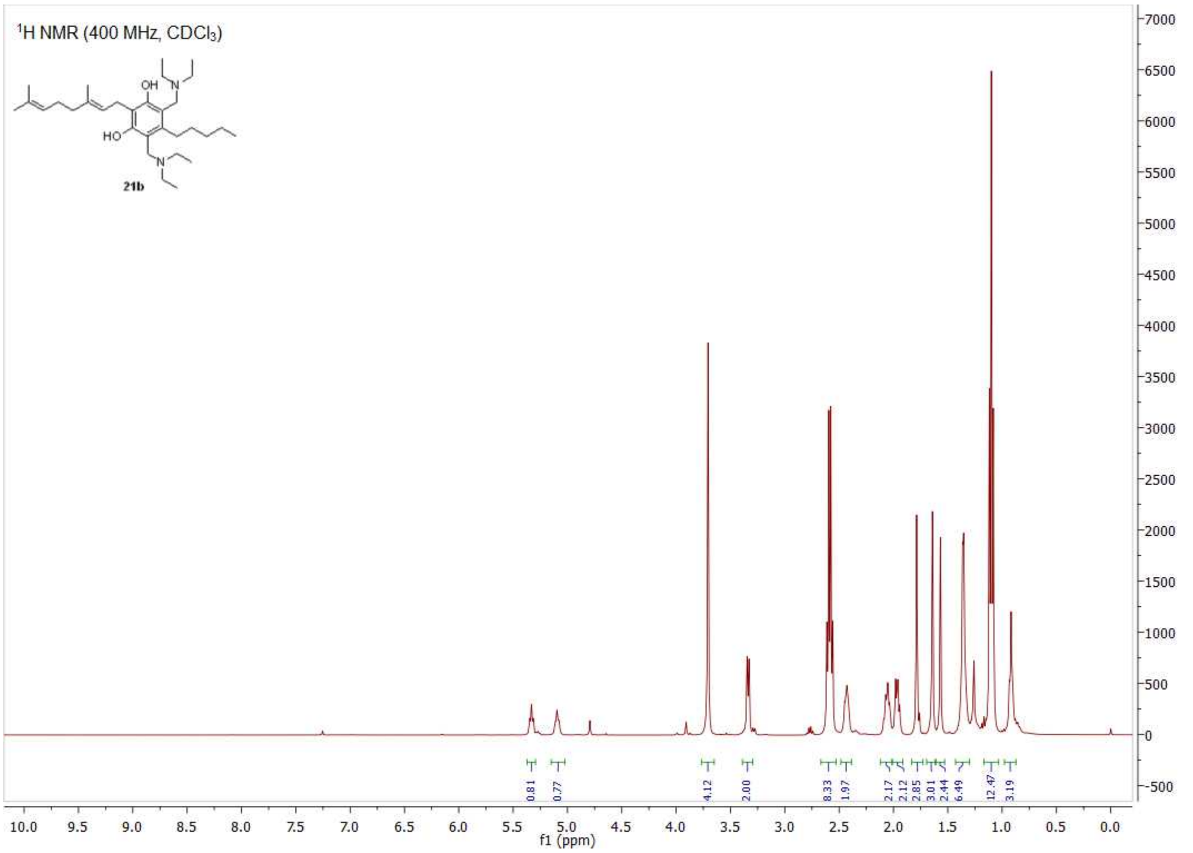

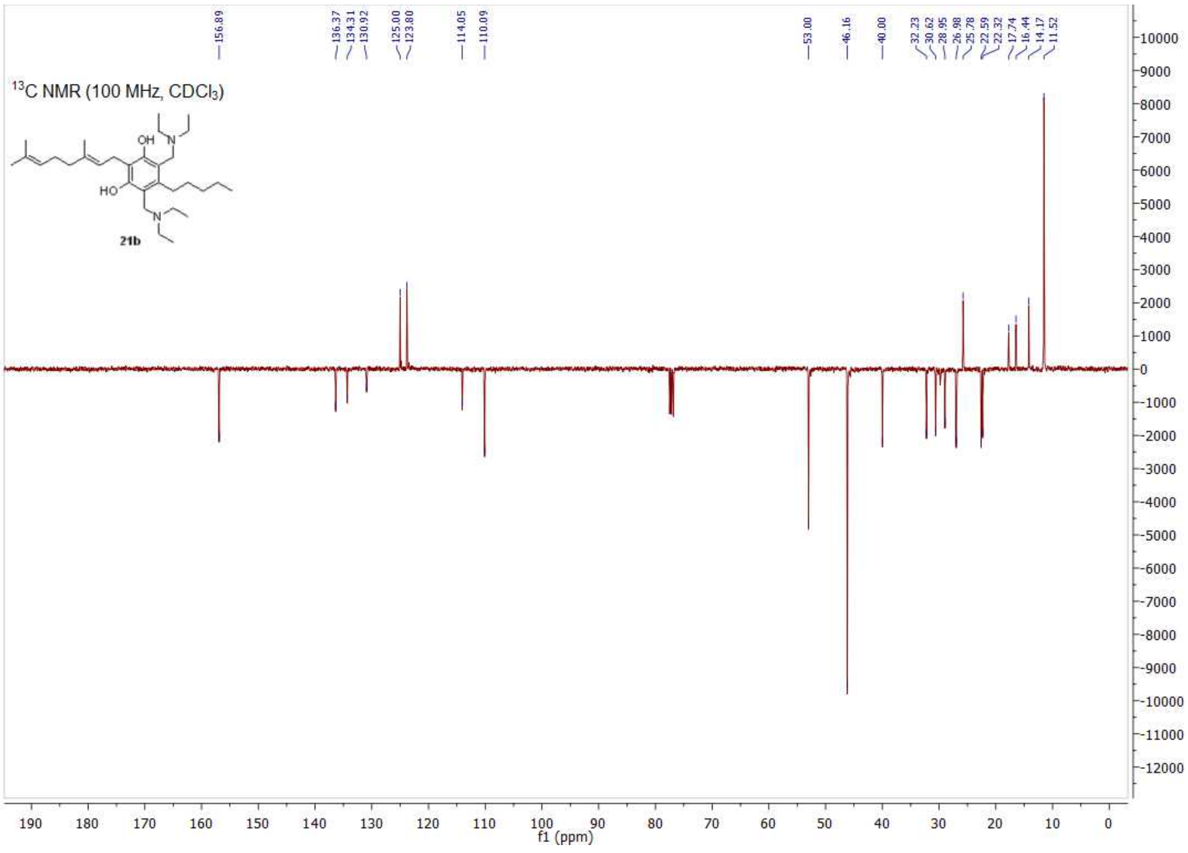

Supplement: Supplementary file 1 — Supplementary Information. [file 41598_2023_45565_MOESM1_ESM.pdf]
